# Evaluation of variation in preclinical electroencephalographic (EEG) spectral power across multiple laboratories and experiments: An EQIPD study

**DOI:** 10.1371/journal.pone.0309521

**Published:** 2024-10-29

**Authors:** Tim P. Ahuis, Magdalena K. Smyk, Clément Laloux, Katharina Aulehner, Jack Bray, Ann-Marie Waldron, Nina Miljanovic, Isabel Seiffert, Dekun Song, Bruno Boulanger, Mathias Jucker, Heidrun Potschka, Bettina Platt, Gernot Riedel, Patrizia Voehringer, Janet R. Nicholson, Wilhelmus H. I. M. Drinkenburg, Martien J. H. Kas, Steven C. Leiser

**Affiliations:** 1 Groningen Institute for Evolutionary Life Sciences (GELIFES), Neurobiology, University of Groningen, Groningen, The Netherlands; 2 Department of CNS Diseases Research, Boehringer Ingelheim Pharma GmbH & Co. KG, Biberach, Germany; 3 Department of Neuroscience, Janssen Research & Development, A Division of Janssen Pharmaceutica NV, Beerse, Belgium; 4 Pharmalex, Mont-Saint-Guibert, Belgium; 5 Institute of Pharmacology, Toxicology, and Pharmacy, Ludwig-Maximilians- Universität (LMU), Munich, Germany; 6 Institute of Medical Sciences, University of Aberdeen, Foresterhill, Aberdeen, Scotland, United Kingdom; 7 Translational EEG, PsychoGenics Inc., Paramus, New Jersey, United States of America; 8 Department of Cellular Neurology, Hertie Institute for Clinical Brain Research, University of Tübingen, Tübingen, Germany; Institute for Research in Fundamental Sciences, ISLAMIC REPUBLIC OF IRAN

## Abstract

The European Quality In Preclinical Data (EQIPD) consortium was born from the fact that publications report challenges with the robustness, rigor, and/or validity of research data, which may impact decisions about whether to proceed with further preclinical testing or to advance to clinical testing, as well as draw conclusions on the predictability of preclinical models. To address this, a consortium including multiple research laboratories from academia and industry participated in a series of electroencephalography (EEG) experiments in mice aimed to detect sources of variance and to gauge how protocol harmonisation and data analytics impact such variance. Ultimately, the goal of this first ever between-laboratory comparison of EEG recordings and analyses was to validate the principles that supposedly increase data quality, robustness, and comparability. Experiments consisted of a *Localisation* phase, which aimed to identify the factors that influence between-laboratory variability, a *Harmonisation* phase to evaluate whether harmonisation of standardized protocols and centralised processing and data analysis reduced variance, and a *Ring-Testing* phase to verify the ability of the harmonised protocol to generate consistent findings. Indeed, between-laboratory variability reduced from Localisation to Harmonisation and this reduction remained during the Ring-Testing phase. Results obtained in this multicentre preclinical qEEG study also confirmed the complex nature of EEG experiments starting from the surgery and data collection through data pre-processing to data analysis that ultimately influenced the results and contributed to variance in findings across laboratories. Overall, harmonisation of protocols and centralized data analysis were crucial in reducing laboratory-to-laboratory variability. To this end, it is recommended that standardized guidelines be updated and followed for collection and analysis of preclinical EEG data.

## Introduction

Currently, a replicability crisis is ongoing in many scientific disciplines, including biomedical and preclinical research [1–7]. This crisis refers to the proportion of published discoveries that cannot be replicated in subsequent experiments, affecting the trustworthiness of science [8, 9]. For example, Bayer Healthcare Germany reported that in only about 25% of cases a sufficient validation of findings for project continuation is provided [10]. There is no consensus on the definition of replicability, however, it is often defined as a repetition of a confirmatory experiment in an almost identical way by an independent laboratory where results largely comply with the findings reported in the original experiment [8, 11]. Ultimately, as replicability is one of the fundamental mechanisms on which science relies to test if research findings are robust, it also determines the speed at which new discoveries are made.

Apart from slowing down knowledge gain, limited replicability between institutes and limited translatability to clinical trials have obvious financial and ethical consequences [11, 12]. Although the replicability crisis recently gained increased attention, illustrated by the number of publications mentioning the crisis, no overarching strategies have been proposed to overcome this problem [13–15]. To effectively increase replicability, it is important to define the factors that most influence data variability. Conclusively, increasing replicability is important for enhancing trustworthiness of results, accelerating knowledge gain, and more efficient use of resources.

Another confounding matter is the increased complexity of methodology of biological experiments, leading to higher costs, bigger data sets, and protocols that are more difficult to master [16]. More complex protocols also lead to more variables that can differ between institutes and increased technical variability, apart from just biological variability (for example regarding reverse transcription quantitative polymerase chain reaction, see [17]). This, together with bias in study designs, study conduct, reporting, and in interpretation of statistics decreases the chance to successfully replicate a finding [4, 18–20].

On this note, it has been convincingly shown in simulations based on a database of 8746 preclinical animal studies that multi-laboratory studies have highly increased chances of finding the actual effect size compared to single-laboratory studies while keeping the single-study sample size (and thus power) constant [21]. The probability of discovering the true effect size increased with the number of laboratories. In the same line, the probability of finding a false negative decreased with an increasing number of laboratories. Voelkl et al. [21] interprets this as being due to the decreased standardisation as a consequence of testing in different laboratories. Therefore, this study advocates for a more limited attempt to complete standardisation in research practices as some heterogenisation might increase the effectiveness of multicenter studies to find the true effect.

This report focuses on a multicenter effort that has been conducted as part of the European Union’s Innovative Medicines Initiative project (EU-IMI) called the European Quality in Preclinical Data consortium (EQIPD). It is dedicated to the identification of the factors in preclinical research that most impact data variability. The ultimate aim of the consortium was to improve replicability in preclinical research with an overarching goal to accelerate drug development. The investigations were done in three phases: Localisation (phase 1), Harmonisation (phase 2), and Ring-Testing (phase 3), and three experimental paradigms were used: open field test [14], Irwin test, and electroencephalography (EEG). The current report will focus on the results obtained in the EEG experiments. EEG was one of the metrics assessed by European Quality In Preclinical Data (EQIPD) because of its high utility in preclinical drug discovery and development and as putative translational biomarkers [22–24].

The use of electroencephalography (EEG) in research has grown exponentially in the last two decades with refinement in technologies, methodologies, and analytical tools emerging throughout this time frame. EEG has bloomed in preclinical and clinical research programs of academic research laboratories and non-profit, biotech, pharmaceutical, and commercial companies utilizing EEG to characterize disease and disease models, screening and development of novel therapeutics, safety assessments, and expanding our neurophysiological understanding of neural circuits [22–28]. The broad applications of EEG make it an ideal paradigm of study in the EQIPD framework. In rodent quantitative EEG (qEEG) studies, the main factors influencing data outcomes are thought to be (1) EEG signal quality (electrode and recording hardware types, surgical quality, electrode location, etc.), (2) EEG recording parameters (sampling rate, filters, gain, location of amplification), (3) EEG analyses, including choice of algorithms, Fast Fourier Transformation (FFT) parameters, filters, etc., (4) the definition and removal of artifacts and (5) manual review criteria for visual verification of sleep stages or epileptiform activity or automated sleep scoring or seizure detection approaches [29–31]. Signal quality can be influenced by broad aspects of an experiment ranging from surgical techniques and surgeon experience to the acquisition system, experimental room environment, and data analysis. As it is not possible to completely unify all parameters, accurate and extensive reporting on the methodology is necessary on at least the crucial details as defined above and preferably including their source codes [32].

## Materials and methods

The study was conducted in three phases: Localisation, Harmonisation and Ring-Testing. The following academic and industrial partners participated in the study (listed here alphabetically): Boehringer Ingelheim (Biberach an der Riss, Germany), Janssen Pharmaceutica (Beerse, Belgium), Ludwig-Maximilians-Universität München (LMU, Munich, Germany), PsychoGenics (Paramus, NJ, USA), University of Aberdeen (Aberdeen, Scotland), and University of Groningen (RUG, Groningen, The Netherlands). Each of the 6 partners was randomly assigned a value from 1 to 6 to blind the data and are referred to as Lab 1–6 in this manuscript. Lab 6 only participated in the Ring-Testing phase. The qEEG experiments were conducted on adult female mice in all phases. In the Localisation phase the goal was to identify the factors that most influence the between-laboratory variability of routine preclinical neuroscientific testing. Mouse qEEG experiments based on local institutional protocols were conducted and data was analysed locally. Based on outcomes, factors deemed highly influential to the between-laboratory variability were chosen for harmonisation in the second phase of experiments. Thus, to test the hypothesis whether harmonisation would reduce that between-laboratory variability, a partially standardized protocol was created across the participating research sites. In addition, centralised analysis was introduced from the Harmonisation phase onwards. In the third and final Ring-Testing phase, a compound was introduced into the qEEG experiment to verify the ability of the harmonised protocol to generate consistent findings. It was hypothesized that between-laboratory variability would reduce from Localisation to the Harmonisation phase, and this reduction would remain during the Ring-Testing phase. The main comparisons between phases were laboratory-to- laboratory variability differences between the Localisation and Harmonisation phase and the effect of local and central analysis in the Ring-Testing phase. Ultimately, the goal was to validate the principles supposedly increasing data quality, robustness, and comparability.

### Animal research

All animal procedures were carried out following the regulations of Directive 2010/63/EU and adhered to the ARRIVE 2.0 Guidelines [33]. Ethical approval for the conduct of these experiments were obtained locally by all participating research sites. As animal research was conducted across multiple laboratories, specific animal care and handling procedures and anaesthesia are specified in the supporting information S1 Table (Localisation Phase), S2 Table (Harmonisation Phase), and S3 Table (Ring-Testing Phase). To maintain the blinding of data reported the following also addresses the specific ethical approvals across the sites. Work was conducted under the Ethical Committee on Animal Experiments (ECD) of Janssen Pharmaceutica NV. Additionally, procedures were approved by local ethical review, a UK Home Office project licence and complied with the EU directive 63/2010E and the UK Animal (Scientific Procedures) Act 1986 and work was reviewed by the Ethical Review Committee (ERC19-20:06) under the University of Aberdeen’s Animal Welfare and Ethical Review Body (AWERB) and approved by a Home Office Project Licence (PP2213334). Additionally, animal experiments were conducted and reported in accordance with German law for animal protection and with the European Directive 2010/63/EU and animal experiments were approved by the government of Upper Bavaria (Munich, Germany, license number: ROB-55.2-2532.Vet_02-18-45) and/or by the ethics committee (Regierungspräsidium Tübingen, 72072 Tübingen, Germany) and experimental procedures were carried out in compliance with the ARRIVE (Animal Research: Reporting of In Vivo Experiments) guidelines and the Basel declaration (http://www.basel.declaration.org) including the 3R principle. Additionally, experiments were conducted according to established protocols and Standard Operation Procedures approved by PsychoGenics’ IACUC in accordance with US law and under the supervision of the Attending Veterinarian, Program of Veterinary Care (PVC) team, and the IACUC committee of PsychoGenics.

Animals were group-housed before surgery but were mostly single-housed after surgery to avoid injury to the implanted animals. Food and water were provided ad libitum. Animals were housed in conventional or IVC housing with 12h:12h light cycle and temperature range of 20–24°C with humidity around 55% but ranging 30–70%. Environmental enrichment was provided (animal houses, nesting material, wood block, nylon bone, or paper roll) and regular handling of animals was performed frequently by tail handling or scruffing. All animals were monitored regularly, including body weights, and only animals that were healthy-looking and displaying normal behaviours such as eating, grooming, exploring, and nesting were used in experiments. Euthanasia methods varied by laboratory and were in compliance with local policies and procedures. Euthanasia was performed by CO2, or animals were anaesthetised using an overdose of sodium pentobarbital and intracardiac perfusion performed with saline and paraformaldehyde followed by decapitation and removal of brain tissue and storage, or decapitation after transcardial perfusion and cervical dislocation. Additionally, mice received metamizol (100 mg/kg in 10 ml/kg, p.o.) 30 minutes before euthanasia with pentobarbital (600 mg/kg in 10 ml/kg, i.p.), then transcardially perfused with 4°C PBS (phosphate-buffered saline) for five minutes. For histology, brains were then removed and split into hemispheres. The left hemisphere was snap frozen on liquid nitrogen. The right hemisphere was placed in 4% PFA (paraformaldehyde) for two days, transferred to 30% sucrose in PBS until it sank (all at 4°C) and then frozen with Tissue-Tek by placing it in 2-methybutane on liquid nitrogen. All samples were stored at -80°C and then shipped for centralized histological analysis. Tail samples were collected for genotype confirmation with PCR.

### Phase 1. Localisation

Participating sites were allowed to follow their own in-house protocol to conduct a 48-hour qEEG recording in mice. Only a few harmonised factors were required at this stage namely animal strain, sex, source, age, group size, light schedule, ad libitum availability of food and water, Bregma coordinates for the epidural screw recording electrodes, length of the recording, surgery recovery time (>7 days) and start time of the recording (Zeitgeber time). For an overview of the experimental factors, see S1 Table. Note, some laboratories performed 72-hour recordings, however, only 48 hours were analysed. Furthermore, a protocol for data analysis was agreed upon in advance so that all data would be delivered in the same format and would adhere to basic pre-processing steps.

A commonly used mouse model for Alzheimer’s Disease (AD), the transgenic (TG) Tg4510 mice and their wild type (WT) controls (n = 12 for each group) were used in the study. Mice were either bred internally from breeding pairs obtained through The Jackson Laboratory (Bar Harbor, US; stock number 024854) in the site’s own vivarium, at a commercial breeding facility, or were obtained from another partner. TG mice were Hemizygous for Tg(Camk2a-tTA)1Mmay, Hemizygous for Fgf14Tg(tetO-MAPT*P301L)4510Kha and the WT mice were Hemizygous for Tg(Camk2a-tTA)1Mmay, Noncarrier, unless otherwise stated. The TG mice aggressively accumulate tau protein by human tauP301L overexpression that forms into cortical tangles and eventually produces strong neurodegeneration in the forebrain, losing up to 40% of the total brain weight, starting at 5.5 months of age [34–37]. These brain changes are very much like what happens in the human AD brain, except for the absence of Aβ pathology. Tau hyperphosphorylation and aggregation are the major hallmarks in this mouse model. The tetracycline-controlled transactivator (tTA) control mice do not overexpress tau protein as the tetracycline operon (tetO) mutation is not present and they only contain the tetracycline activator through which the expression of tetO can be regulated in the full transgenic (TG) mice.

At 21 weeks of age mice underwent surgical implantation of epidural electrodes. It was required that at least one epidural electrode was placed above the hippocampus at the following stereotactic coordinates: AP = -2 mm, ML = +/-1.5 mm with skull flat and Bregma 0–0. An epidural reference electrode was placed above the cerebellum (AP = -5.7–6.2 mm, ML = 0 mm). Every institute conducted a 48-hour EEG recording in a 12:12 light-dark cycle. Some partners recorded for 72 hours, however, only the first 48 hours were considered for further analysis. Recorded signals were subjected to FFT (Fast-Fourier-Transformation), the power calculated for 10 second epochs for the following frequency bands: Delta (1–4 Hz), Theta-1 (4–6 Hz), Theta-2 (6–8 Hz), Alpha-1 (8–11 Hz), Alpha-2 (11–14 Hz), Beta-1 (14–18 Hz), Beta-2 (18–32 Hz), and Gamma-1 (32–48). Power spectral means and standard error of the mean (SEM) calculated in 30-minute time bins were uploaded to the EQIPD database. Within 3 days after the recording, animals were transcardially perfused with cold phosphate-buffered saline (PBS) or saline at 4 degrees Celsius, brains were removed, and hemispheres separated. One hemisphere was immersed in 4% paraformaldehyde (PFA) for 2 days and then transferred to PBS; the other hemisphere was snap-frozen on either dry ice or with liquid nitrogen. Samples were labelled and sent for histological examination to M.J. (University of Tübingen).

### Phase 2. Harmonisation

Based on the input provided by other EQIPD work packages (e.g. work package 2, WP2 on literature studies and historical data collection, and WP3 on guiding principles to improve data robustness [38] a priority list was created with experimental environmental factors that were deemed important in producing variability. All partners participating in this phase indicated which factors could be standardised given the limitations of their institution. Factors to which all partners could harmonise were then selected and became part of the harmonised protocol. Animal strain, sex and age (at the time of surgery and recordings) of the animals were harmonised. Considering animal husbandry, group-housing, light-dark cycle, feeding schedule, handling frequency and handling methods were agreed and harmonised. Also, surgery and experimental performance such as type of surgery, electrodes and their placement, duration of the recovery period, habituation and actual recordings, and general requirements regarding data analysis were harmonised as much as possible across participating sites (see S2 Table).

Similar to Localisation, the Harmonisation phase experiments were conducted on female Tg4510 mice and their WT controls (n = 12 for each group) bred either inside the institutions’ vivarium, at a breeding facility or were obtained from another partner. Animals were group-housed (4 per cage before the surgery, 1–3 mice per cage after the surgery, genotype matching) in standard laboratory conditions with 12:12 light-dark cycle. Ad libitum food and water and cage enrichment (the type of enrichment not strictly specified) were provided both, before and during experimental recordings. At 20–21 weeks of age mice underwent stereotactic surgery during which an epidural screw electrode was implanted above the hippocampus at the following coordinates: AP = -2 mm, ML = -1.5 mm with skull flat and Bregma 0–0. An epidural screw reference electrode was placed above the cerebellum (approximate coordinates from Bregma were -5.7 mm posterior and midline). Mice were allowed to recover from the surgery for 14 days (instead of 7 days during localisation). During this time, animals were handled twice a week by the tail with gloved hands by the handler blinded from the experimental groups.

At 22–23 weeks of age mice were subjected to 48-hour continuous EEG recordings preceded by a 48-hour habituation period to the recording environment. Recording started at the onset of the 4th hour of the light cycle (Zeitgeber time ZT4) and continued for 48 hours ending at ZT4 on day 3. The study design was randomized using an R-script provided by Anton Bespalov that divided groups evenly based on exact age, and the experimenter was blinded for experimental groups throughout the study, which was not a requirement in the localisation phase.

In general, the EEG analysis at the different sites followed the historical analysis with power spectral density plots consisting of standardized subdivisions. Recorded signals were subjected to FFT, the power calculated for 10 second epochs for the following frequency bands: Delta (1–4 Hz), Theta-1 (4–6 Hz), Theta-2 (6–8 Hz), Alpha-1 (8–11 Hz), Alpha-2 (11–14 Hz), Beta-1 (14–18 Hz), Beta-2 (18–32 Hz), and Gamma-1 (32–48), and averaged for each 1-hour time bin for the 48-hours recording and represented as a mean and SEM for Tg4510 and WT mice. Some partners provided sleep scoring which consisted of the following stages: active wake, quiet wake, NREM sleep, and paradoxical or REM sleep and were defined using classical criteria [24, 39, 40]. Results (power spectral means and SEM calculated in 1 hour time bins) were uploaded to the EQIPD database for centralised analysis. Data were processed locally by the partners and were also subjected to a centralised analysis performed by S.C.L. (PsychoGenics, USA).

### Phase 3. Ring-Testing

In the final phase, a pharmacological compound (MK-801) was selected for testing at the partner institutions to further assess intra- and inter-laboratory variability and test the performance of the harmonised protocol in a blinded-compound testing context. Again, all partners participating in this phase indicated which factors could be standardised given the limitations of their institution (see S3 Table). MK-801 (dizocilpine) was selected as the compound to test because the behaviour and neural oscillation alterations induced by different doses of MK-801 have been well characterised since its discovery in 1982 (e.g. [41–43]). Furthermore, both local and central analyses were conducted in this phase so that we could separate the influence of this from the harmonised protocol.

Female, 12–14 weeks old C57BL/6J mice (n = 12) were used in the experiment. Vendor diversity was allowed since it was not possible for the participating partners to obtain animals from the same source but the importance of selecting and characterising control subjects regarding background, sex, and supplier to ensure proper experimental outcomes in biomedical research cannot be stressed enough [44]. Variables regarding housing, handling, surgery, recording sites, recovery period were as specified for the harmonisation stage. Mice underwent stereotactic surgery of epidural electrodes at the following locations: above the hippocampus (AP = -2 mm, ML = -1.5 mm with skull flat and Bregma 0–0) for the recording electrode, and above the cerebellum for the reference (AP = -5.7 mm, ML = 0 mm). After the recovery period, animals entered the cross-over designed testing phase. When not recorded in their home cages, mice were habituated to the recording environment for at least 30 min. Next, one hour of a baseline was recorded after which the reference compound, MK-801 at the doses of 0.05, 0.2 and 0.8 mg/kg dissolved in saline, was injected i.p. between the second and fourth hour of the light phase (Lab 1 and Lab 3 used only the 0.2 mg/kg dose). Only one laboratory (Lab 6) incorporated the 0.8 mg/kg dose. Upon administration of this high dose a robust decrease in body weight was observed and thus all other partners were notified to not test this dose. The recordings were continued for 4 hours. Mice were allowed at least 2 days washout period between dosing. Every dosing day was preceded by a baseline day during which signals were recorded for the same number of hours, however, no injection was performed.

### Data analysis

**Fig 1:**
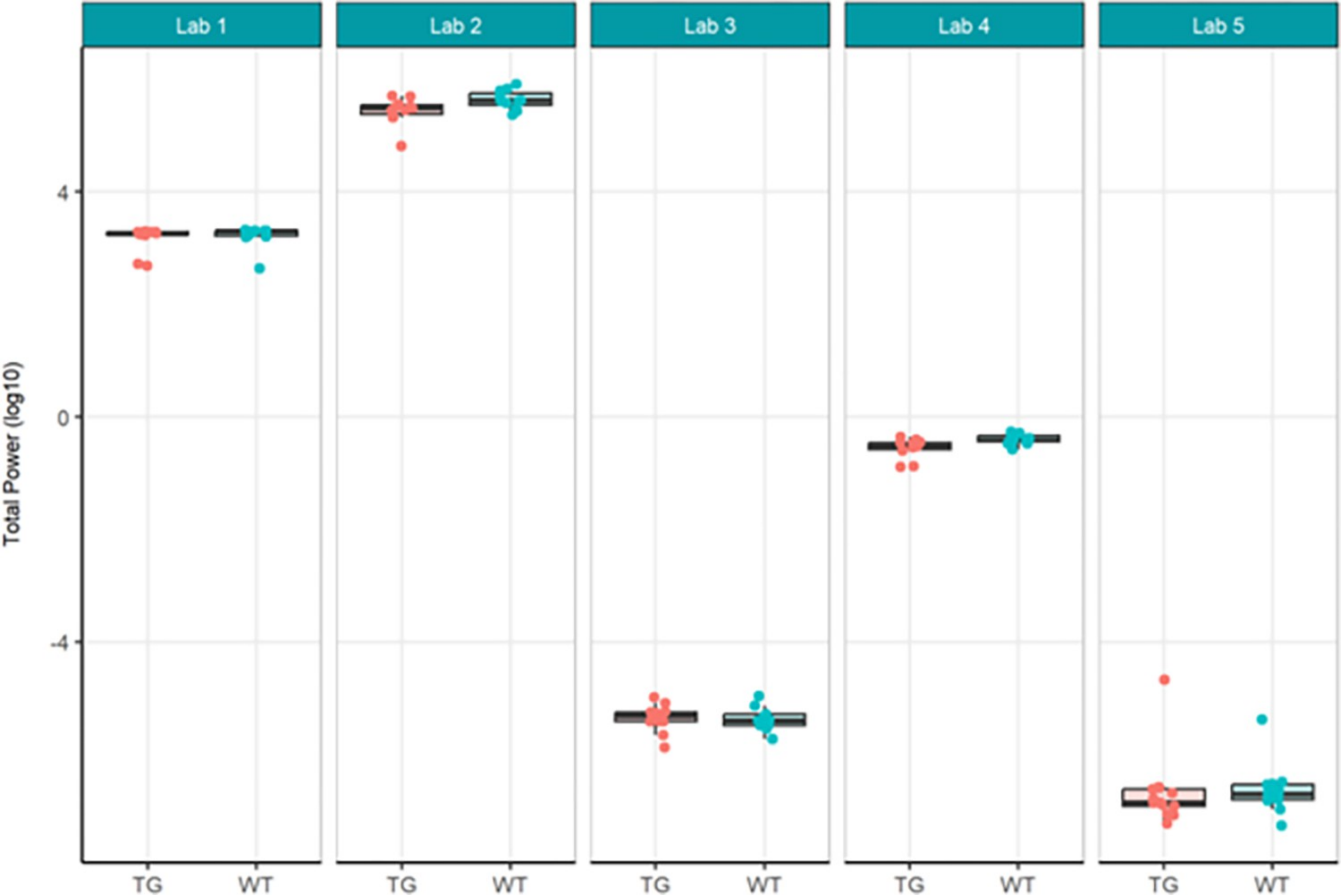
Localisation phase total power analysed locally by the partners. Tukey boxplots and individual data points for the log_10_ total power values per genotype group for each participating laboratory during phase 1. Lab 2 and Lab 4 found significant reduction in total power in Tg4510 mice compared to WT controls (see S4 Table).

The primary objective of these experiments was to evaluate reproducibility across laboratories. In Localisation Phase, Total Power was evaluated initially to understand the feasibility of directly comparing EEG across laboratories given no prior harmonisation. As may be expected given the instrumentation of different recording equipment, the Total Power was vastly different (see Fig 1). For this reason, relative power (see Fig 3), which normalised each recording to the total power, was chosen to facilitate comparison across laboratories. Although all frequency bands were generated not all are included herein. Only Theta power was chosen as the main comparator when assessing whether there was a difference between genotypes, because of prior unpublished data as well as growing data in the literature that theta may represent hyperexcitability seen in animal models of and patients with Alzheimer’s disease (not reviewed here) [22, 33, 35]. The outcomes drawn from theta power can be understood to be similar for all other frequency bands generated. For Harmonisation Phase the same comparators, Total Power (see Fig 6) and relative theta power (see Fig 8) were used to evaluate whether the harmonisation procedures had a direct impact on the reproducibility of results across labs. In the final Ring-Testing Phase, again all frequency bands were evaluated yet the within and between laboratory comparisons were restricted to gamma power because that has been most abundantly reported in the literature and can be viewed as a reliable change following MK-801 treatment. The centralised analysis of all frequency bands is provided as Supplementary material. (S1 Fig) provides estimated means (bars) and individual data points for absolute power, relative power, or percent change from baseline calculated using absolute power across all frequency bands for each treatment group (vehicle, MK-801 at 0.05 and 0.2 mg/kg) when tested during the Ring-Testing Phase by Laboratory analysed centrally. These data were not further compared statistically.

#### Data analysis: Within- and between-laboratory analyses

Data analysis consisted of two types of analyses: within-laboratory and between-laboratory analyses. The within-laboratory analyses focused on the genotype effect (Tg4510 vs WT mice), on the total power and relative theta power of the EEG signal, which were the outcome measures agreed upon in phase 1 and phase 2, and the dose-dependent effect of MK-801 on relative gamma power and percent change of gamma power, which were the outcome measures agreed upon for phase 3.

The within-laboratory analysis investigates how conclusions vary if each laboratory would do the statistical analysis internally (if not analysed centrally). The between-laboratory analyses focused on the explained variance due to the contributor (ContributorID), treatment effect variability between laboratories (ContributorID:TestgroupID), and the biological variability (residual), and thus investigated what part of the data variability was related to laboratory-to-laboratory differences. Levels of the ContributorID, interaction and residual were estimated in the Localisation phase. Then, it was expected that increased harmonisation would lead to a lower total variability of ContributorID and the interaction term, while the residual biological variance would remain similar. In the Ring-Testing phase the goal was to see if harmonisation held up in blinded compound testing and to investigate the effect of adding central analysis on the between-laboratory variability. Within-laboratory analyses were performed locally by the partners in the first phase. In the second and third phase of experiments, analysis was centralised to reduce variability induced by differences in data processing.

#### Data analysis: Central analysis of the EEG data

The first step in centralised analysis was to set each recording file obtained from separate laboratories to contain the same features. First, Spike2 (Cambridge Electronic Design Limited, UK) was used to delete any unnecessary data (additional EEG channels, electromyography (EMG), and/or activity data) to produce a single channel file. Spike2 was then used to interpolate the EEG signal to 100 Hz, remove direct current (DC) shift, and apply a low pass filter. A finite impulse response, low pass filter was set to 49 Hz to eliminate potential electrical noise from both EU (50 Hz) and US (60 Hz) sources. Next, large-amplitude artifacts, commonly associated with movement, were removed with a custom script in Spike2 that used semi-automated, root mean square (RMS)-based amplitude threshold-crossing artifact rejection. Visual confirmation was employed to ensure that the algorithm set the threshold appropriately and allowed manual correction to ensure only artifacts were removed. For example, with this RMS-based approach a file that contains no or very few large-amplitude artifacts may yield a threshold that is in fact too low and would remove too much real signal. Likewise, if the file contained many high-amplitude artifacts the threshold could be too high and thus not remove sufficient noise. The manual confirmation ensures uniform application of artifact rejection to each unique recording. All “cleaned” or processed files were then converted into EDF file format. Although other software may be able to convert various EEG file formats into EDF, Spike2 was used in this study to convert the files. These EDF files were imported into NeuroExplorer (NEX; Nex Technologies, CO, USA). Within NEX, the time of dosing was entered for any file that needed such delineation. For example, some data were provided as separate files for pre- or post-dose recording, while other files included both pre- and post-dose recordings in the same file. In this latter case, the timestamp of dosing was used to extract specific times that represented pre- and post-dosing. Power spectral density (1–48 Hz) was calculated for the specified time window using NEX with the following settings: number of frequency values, 512; window overlap, 50%; windowing function, Hamming; no normalisation (raw power spectral density, PSD); no log scale; no smoothing or multitaper. NEX employed a Welsh periodogram that was calculated by computing the discrete Fourier transform, and then computing the squared magnitude of the result. The individual periodograms were then time-averaged, which reduced the variance of the individual power measurements and yielded an array of power measurements versus frequency “bin”. The power spectrum was normalized so that the sum of all the spectrum values was equal to the mean squared value of the rate histogram (NEX). The raw (or absolute power) spectral densities were exported to Excel in 481 frequency bins between 1.074 to 47.95 Hz (approximately 0.1 Hz bins). In Excel, relative power for each 1 Hz bin was calculated by dividing the raw power in that 1 Hz bin by the sum of power (total power) from 1 to 48 Hz. Next, both raw and relative power were summed into the following frequency bands: Delta (1–3.9 Hz), Theta-1 (4–5.9 Hz), Theta-2 (6–7.9 Hz), Alpha-1 (8–10.9 Hz), Alpha-2 (11–13.9 Hz), Beta-1 (14–17.9 Hz), Beta-2 (18–31.9 Hz), and Gamma-1 (32–48 Hz). Percent change from baseline was calculated within subject (for each recording independently) on each of these frequency bands as: (post-dose power–pre-dose power)/(pre-dose power)*100%. Average percent change from baseline was calculated per laboratory, treatment group, and frequency band.

In the Ring-Testing phase, relative gamma power (32–48 Hz) was calculated from 30–60 minutes post dose by dividing the sum of power in gamma (32–48 Hz) by the sum (total) power in all frequencies (0.5 to 48 Hz) from 30–60 minutes post dose. This produced a single value per animal which was subsequently averaged for the experimental group (mean ± SEM). Additionally, the percent change in gamma power (32–48 Hz) from 30–60 minutes post dose (t1) was calculated using the 30–60 minutes before drug treatment as the baseline (t0). The percent change from baseline was calculated for each recording using the formula: [(Gamma_t1_ –Gamma_t0_) / Gamma_t0_] *100%. Absolute or raw power was used to calculate the percent change, except where mentioned to compare the differences between calculating percent change using raw versus relative power. The percent change from baseline was subsequently averaged for the experimental group (mean ± SEM).

#### Data analysis: Statistics

Before the analysis a log_10_ transformation was performed to different outcome variables (total power, relative theta power, and relative gamma power) because data are naturally bounded between 0 and + infinity. The log_10_ transformation changes the bound and sets it between -infinity and + infinity which is more aligned with the assumptions of linear modelling. Moreover, the model accounts for those natural bounds when estimating the effects. The statistical model for the localised versus harmonised and the ring-testing non-centralised versus centralised analysis were the same. The goal was to assess how the laboratory-to-laboratory variation was reduced by harmonizing the processes.

To study the laboratory-to-laboratory variation by stage and protocol, two types of models were used. The first one explored the differences in dosing effects by laboratory. This illustrated a situation where each laboratory would perform the comparisons internally and aimed to highlight the variability of estimated differences between each lab. It was expected that the Harmonisation protocol provided more consistent results than the Localisation protocol. A simple linear regression was fitted to the different responses by laboratory with TestGroup as unique fixed effect:

$$Y_{id}=\beta_{0}+\beta_{d}\times {TestGroup}_{d}+\varepsilon_{id}$$ (1)

where *Y*_*id*_ is the response *i* for TestGroup *d*,

*β*_0_ is the intercept of the model (the expected *Y*_*id*_ for TestGroup *d* of reference),

*β*_*d*_ is the effect of TestGroup *d* on *Y*_*id*_ (the expected change in *Y*_*id*_ when TestGroup *d* is considered),

*ε*_*id*_ is the random error (or biological variance in this specific case) associated with *Y*_*id*_: $\varepsilon_{id}∼N(0,\sigma_{\varepsilon }^{2})$.

The TestGroup effects and their contrasts were estimated using the R package emmeans.

The second model explores the differences in dosing effects over all laboratories accounting for the laboratory-to-laboratory variability. It aimed to directly measure the variance associated to differences between laboratories and assess the percentage of total variance it represented. It was expected that the Harmonisation protocol provided lower laboratory-to-laboratory variance than the Localisation protocol, while having similar biological variances. A linear mixed model was fitted to the response with TestGroup as unique fixed effect and laboratory as well as interaction between laboratory and TestGroup as random effects:

$$Y_{idl}=\beta_{0}+\beta_{d}\times {TestGroup}_{d}+b_{l}+d_{dl}+\varepsilon_{idl}$$ (2)

where *Y*_*idl*_ is the response *i* for TestGroup *d* and Lab *l*,

*β*_0_ is the intercept of the model (the expected *Y*_*idl*_ for TestGroup *d* of reference),

*β*_*d*_ is the effect of TestGroup *d* on *Y*_*idl*_ (the expected change in *Y*_*idl*_ when TestGroup *d* is considered),

*b*_*l*_ = the random intercept of laboratory *l*: $b_{l}∼N(0,\sigma_{b}^{2})$,

*d*_*dl*_ = the random intercept of TestGroup *d* and laboratory *l*: $d_{dl}∼N(0,\sigma_{d}^{2})$ and,

*ε*_*idl*_ is the random error (or biological variance in this specific case) associated with *Y*_*idl*_: $\varepsilon_{idl}∼N(0,\sigma_{\varepsilon }^{2})$.

$\sigma_{b}^{2},\;\sigma_{d}^{2}$ and $\sigma_{\varepsilon }^{2}$ are referred as the variance components in this model. In linear mixed model, variance is decomposed in several terms of interest to understand which ones are the main source of variability in the data. In this specific case, $\sigma_{b}^{2}$ is the laboratory-to-laboratory variability, $\sigma_{d}^{2}$ is the variability in differences between TestGroup observed between laboratories and $\sigma_{\varepsilon }^{2}$ is biological variability. The between-laboratory variability assessed in this paper is the sum of $\sigma_{b}^{2}$ and $\sigma_{d}^{2}$. The model was fitted with R package lmer. The TestGroup effects and their contrasts were estimated using the R package emmeans.

## Results and discussion

### Phase 1: Localisation

In the Localisation phase, all partners involved selected using the transgenic (TG) Tg4510 mouse model for Alzheimer’s Disease (AD) and their wildtype (WT) controls. Each laboratory performed a qEEG study on 12 female rTg4510 mice (TG) and 12 female tTA wildtype counterparts based on local standard operating procedures (SOP) for study design, execution, recording equipment and analysis (see S1 Table). The main endpoints for analyses were total power and relative theta power of the EEG signal to evaluate the existence of a genotypic effect as previous literature implicated theta power changes [45, 46] or showed reduced theta power [47] in the Tg4510 mice compared to WT controls. One of the first indicators of a large between-laboratory variability was the strikingly different units of power obtained from the local analyses of total and relative power. Logarithmic transformation of the data was required to present values per laboratory on the same scale (Fig 1).

**Fig 2:**
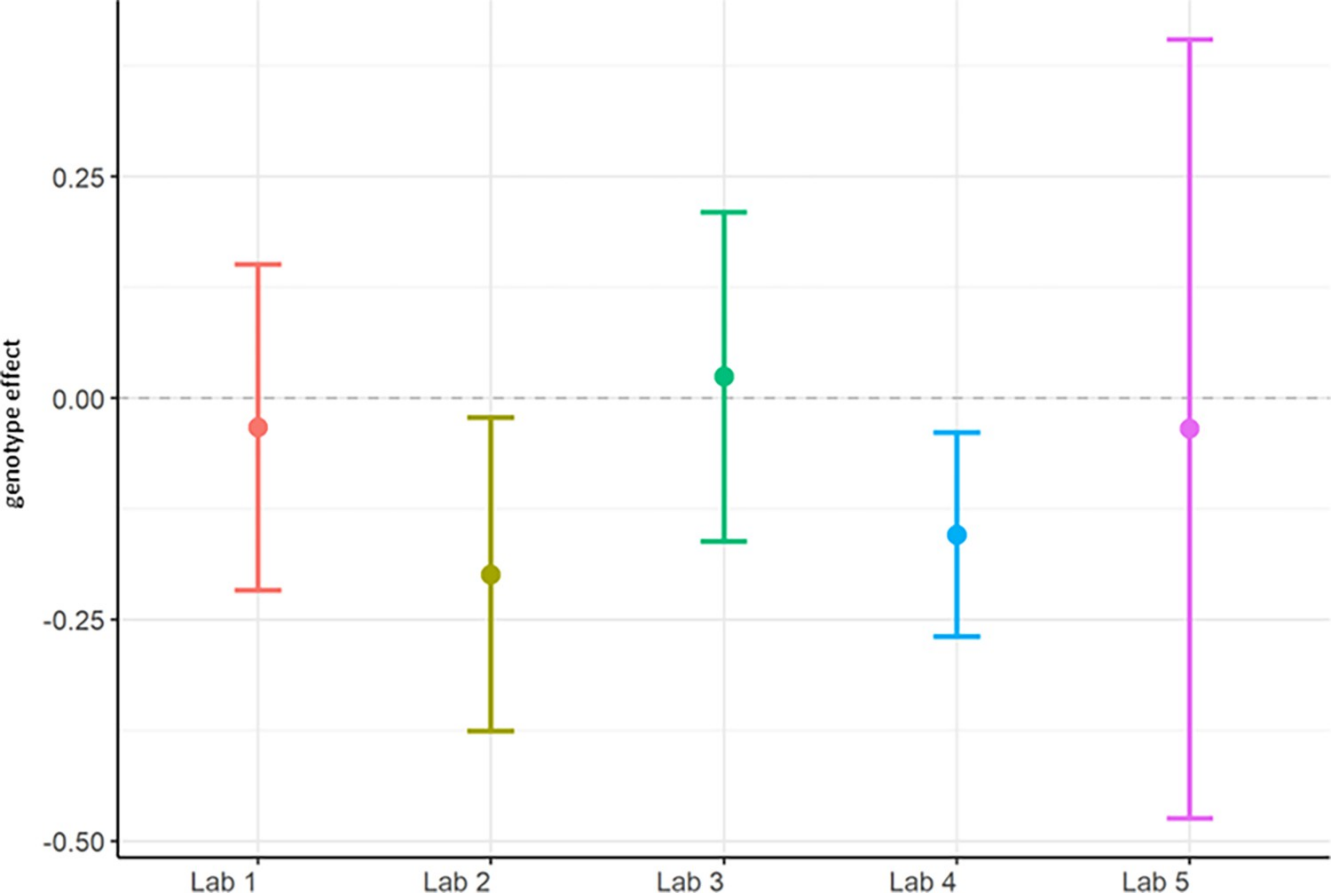
Localisation phase total power analysed locally by the partners. The figure shows estimated means of TG-WT contrasts (genotype effect) with each the lower confidence limit (CL) and higher CL on log_10_ total power data.

The results of total power showed that Lab 2 and Lab 4 found a significant reduction in total power in the TG group compared to WT (Fig 2), consistent with literature [47], although the other partners could not replicate this finding. The across-laboratories analysis (Table 1) showed a variance of 27.8751 (99.67%) for total power that could be explained by the differing conditions per contributor. The TestgroupID:ContributorID interaction was estimated as 0, although it should be noted that the huge variability between laboratories can cause an inability to estimate the variance reliably [48, 49]. The residual variance was only 0.0925 (0.33%), indicating a small amount of variance was due to biological variability. It can be concluded therefore that most (99.67%) of the variability is attributable to the specific conditions of the contributor, their local protocol, EEG data quality, manner of analysis, etc., which will be explored in more detail within this paper.

**Table 1:** *Harmonisation phase total power analysed locally by the partners*. Table displaying the results from the across-laboratories analysis for total power in phase 1 (Localisation) and phase 2 (Harmonisation) displaying the laboratory-to-laboratory variability (ContributorID), genotype effect variability between laboratories (TestgroupID:ContributorID), and residual variability.

Effect	Localisation	Harmonisation
ContributorID	27.8751 (99.67%)	0.0607 (36.23%)
TestgroupID:ContributorID	0 (0%)	0.0029 (1.71%)
Residual	0.0925 (0.33%)	0.1039 (62.05%)
Total	27.9676 (100%)	0.1674 (100.00%)

**Fig 3:**
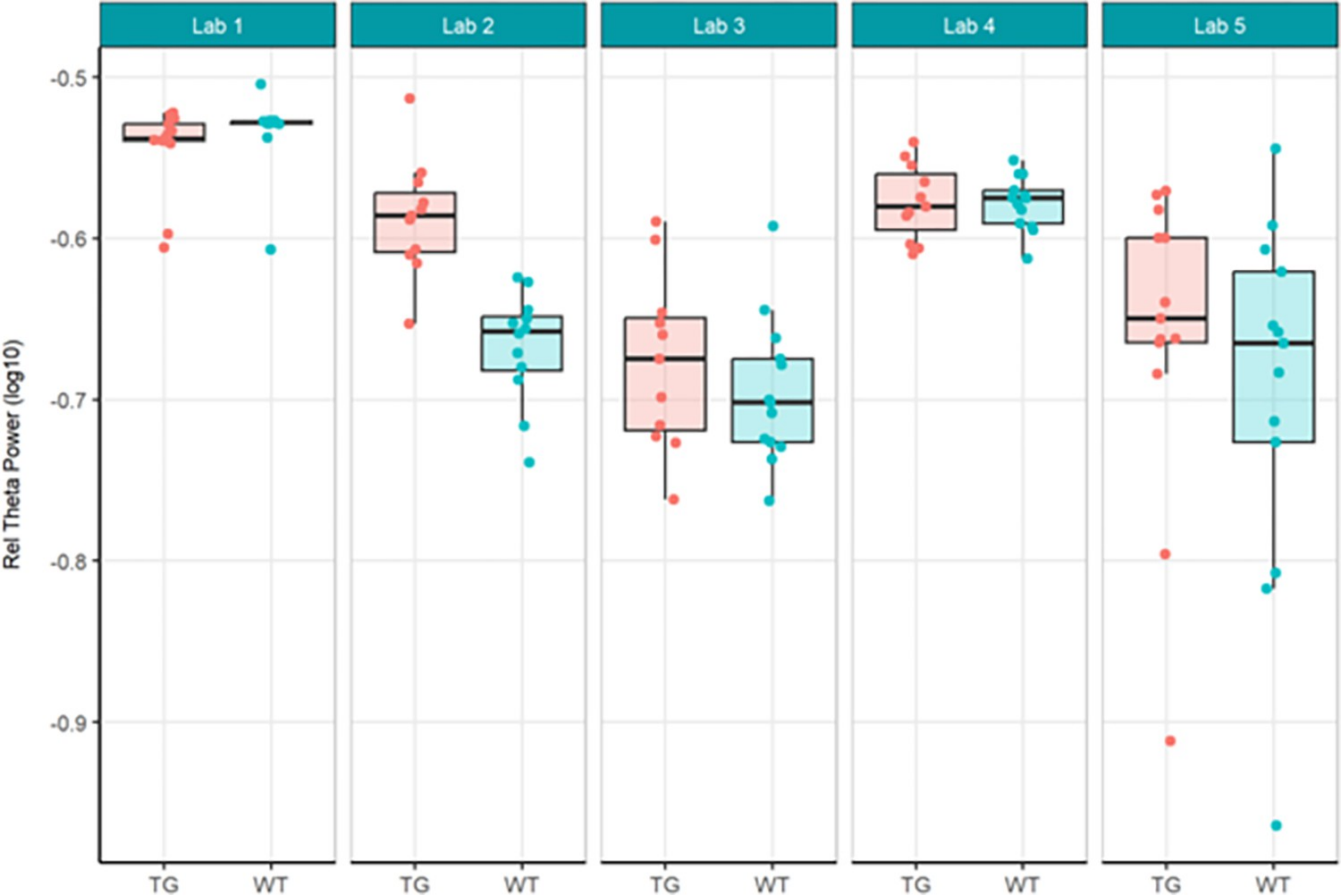
Localisation phase relative theta power analysed locally by the partners. Tukey boxplots and individual data points for the log_10_ relative theta power values (obtained as a percentage of total power for individual subjects) per genotype group and every participating laboratory during phase 1 data collections. Only Lab 2 found a significant increase in relative theta power in Tg4510 mice compared to WT controls (see S5 Table). The boxplot displays individual data points, as well as the median, the first (Q1) and third (Q3) quartiles and the whiskers are based on the interquartile range (IQR; Q3 –Q1) where they are not higher than Q3 + 1.5 * IQR and lower than Q1–1.5 * IQR.

**Fig 4:**
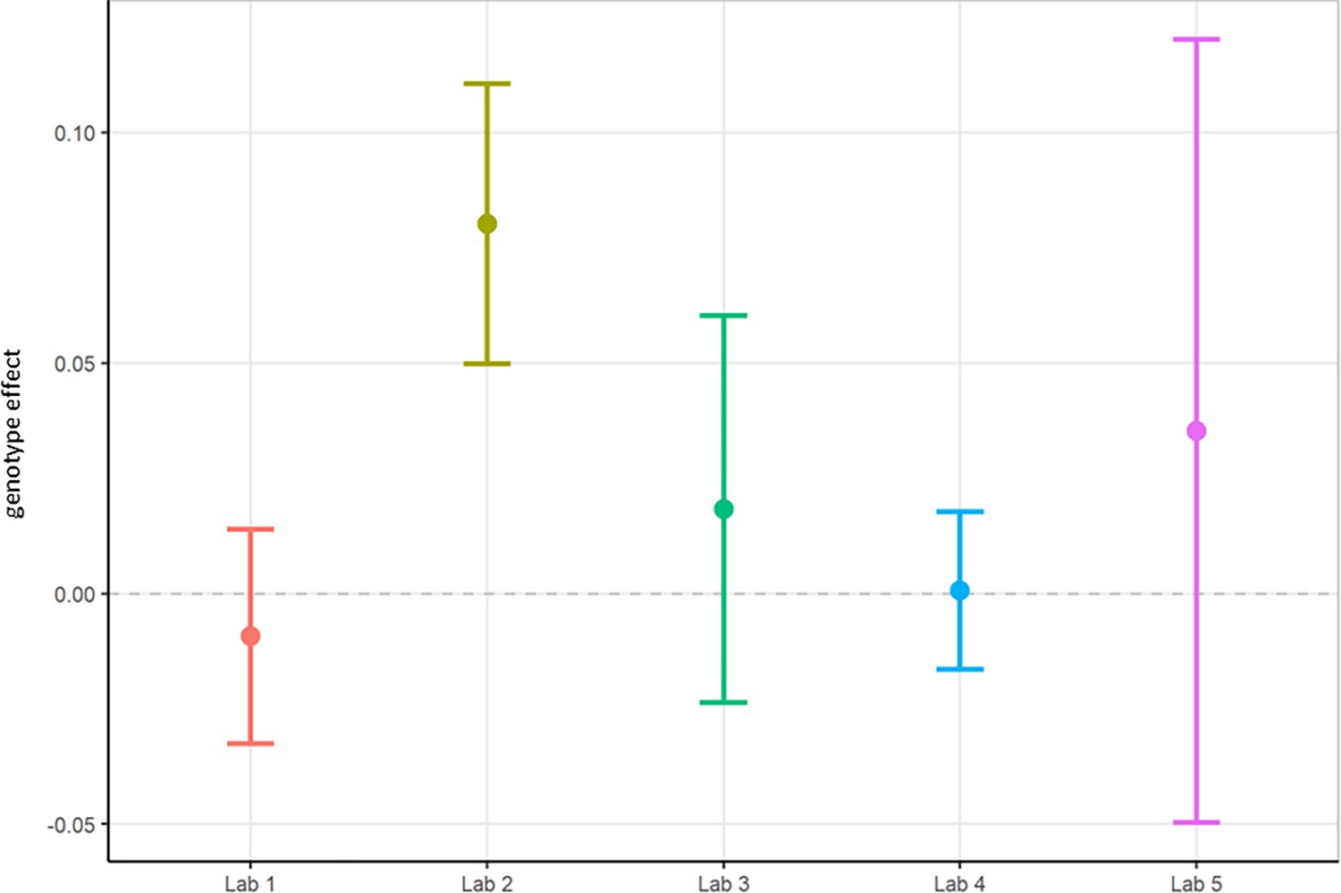
Localisation phase relative theta power analysed locally by the partners. The figure shows estimated means of TG-WT contrasts (genotype effect) with each the lower confidence limit (CL) and higher CL on log_10_ relative theta power data.

Next, relative theta power was examined from the same study. Theta power was chosen as it has been implicated in other AD models [47, 50–52]. There were still large variations in data between institutes, albeit less than for total power. Again, the differences in relative theta power led to difficulties with comparisons. Only Lab 2 obtained a statistically significant increase in relative theta power values between TG and WT mice (Figs 3 and 4), and this was not replicated by other laboratories. The across-laboratories analysis (Table 2) showed a variance of 0.0037 (50.42%) in relative theta power that was attributable to the specific conditions of the contributors. A variance of 0.0003 (4.3%) was attributable to the test group and contributor interaction, while 0.0033 (45.28%) was attributable to biological variability.

**Table 2:** *Harmonisation phase relative theta power analysed centrally*. Table displaying the results from the across-laboratories analysis for relative theta power in phase 1 (Localisation) and phase 2 (Harmonisation) displaying the laboratory-to-laboratory variability (ContributorID), treatment effect variability between laboratories (TestgroupID:ContributorID), and residual variability.

Effect	Localisation	Harmonisation
ContributorID	0.0037 (50.42%)	0.0072 (54.71%)
TestgroupID:ContributorID	0.0003 (4.30%)	0.0000 (0.00%)
Residual	0.0033 (45.28%)	0.0059 (45.29%)
Total	0.0074 (100.00%)	0.0131 (100.00%)

In summary, relative theta power (Figs 3 and 4; S5 Table) was less variable than total power (Figs 1 and 2; S4 Table) since relative power calculation accounts for power differences across recordings. Based on these data obtained during Localisation, large variations in EEG signals were evident between partners when adhering to only their local SOP with minimal alignment across laboratories. Therefore, it is understood that without harmonization across-laboratory comparisons may lack replicability.

**Fig 5:**
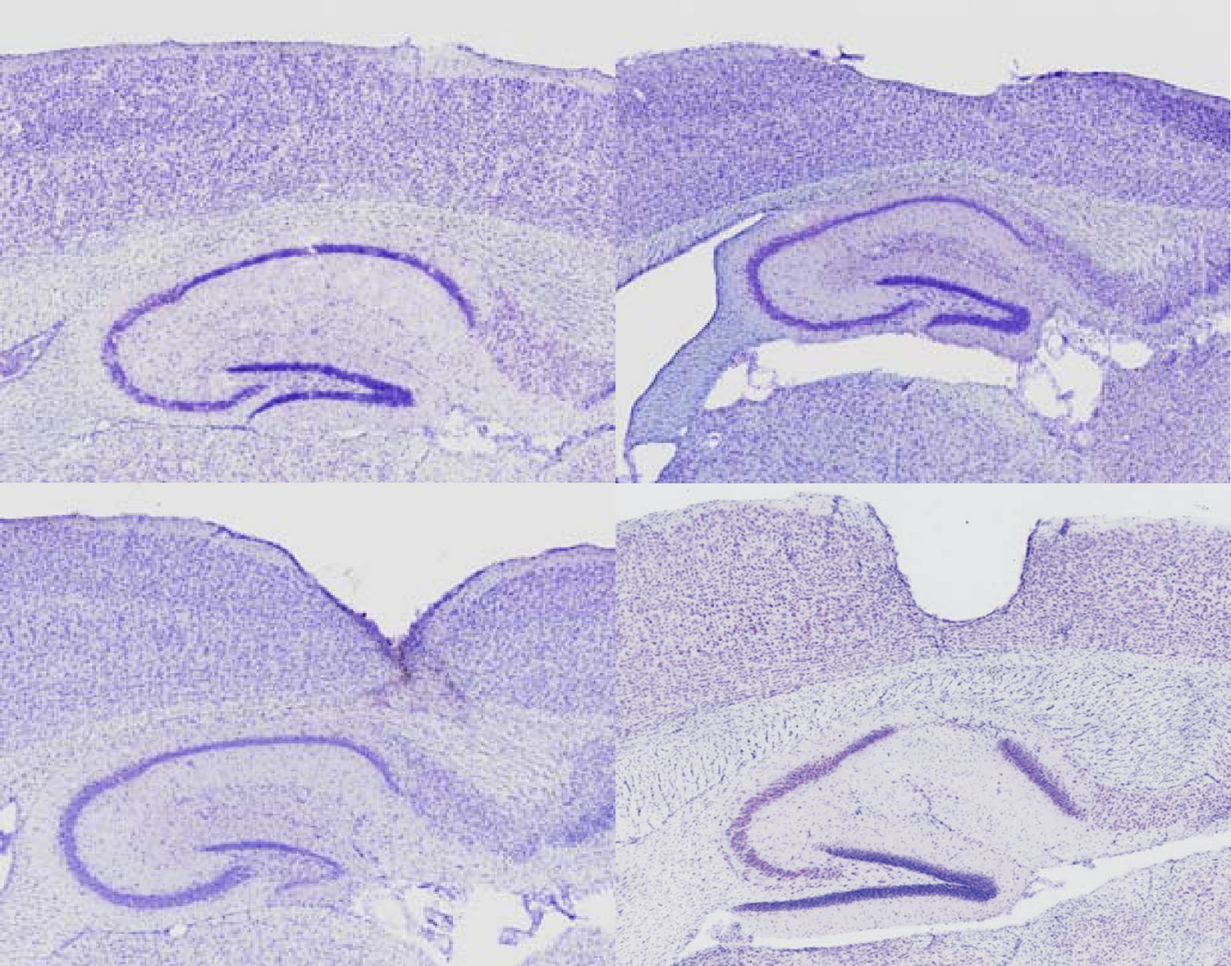
Localisation phase histology. Variability in electrode placement depth and underlying cortical damage that occurred in few animals across multiple laboratories. Increasing damage from top left (good), top right (fair), bottom left (poor), to bottom right (worst), where surgery-induced lesions occurred not only in cortex but also in underlying CA1 due to screw electrode being inserted too deeply. Note, the best EEG implants only contact dura and do not make any damage.

One possible explanation for the high across-laboratory variance in EEG power was hypothesized to be due to the source: the EEG recording itself and the quality of the signal. Firstly, EEG quality is heavily influenced by surgical procedures. That is, if damage occurs to the cortex the signal could be dampened or inexplicit and if the electrical connections are not precise then noise and artifacts can ruin the real EEG signal. Indeed, after the histological investigation of the hemispheres supplied by the partners, high variability in electrode depths and associated lesions were observed. While each laboratory did not deviate substantially on anterior-posterior or medial-lateral coordinates, some laboratories had little to no lesions, while others inserted the electrodes too deeply through the skull, which resulted in lesions in the cortex and in few cases even induced lesions in CA1 (Fig 5). Damage to tissue inherently changes the signal and these differences were present both within laboratory and across laboratories, thereby likely accounting for some of the variance in EEG power.

### Phase 2: Harmonisation

In phase 2, factors deemed important for contributing to between-laboratory variance in a qEEG paradigm were harmonised across partners, results can be seen in S2 Table. The experiment again consisted of a qEEG study in 12 female rTg4510 mice (TG) and 12 female tTA counterparts (WT) and the main endpoints for analyses were total power and relative theta power of the EEG signal to evaluate the existence of a genotypic effect.

**Fig 6:**
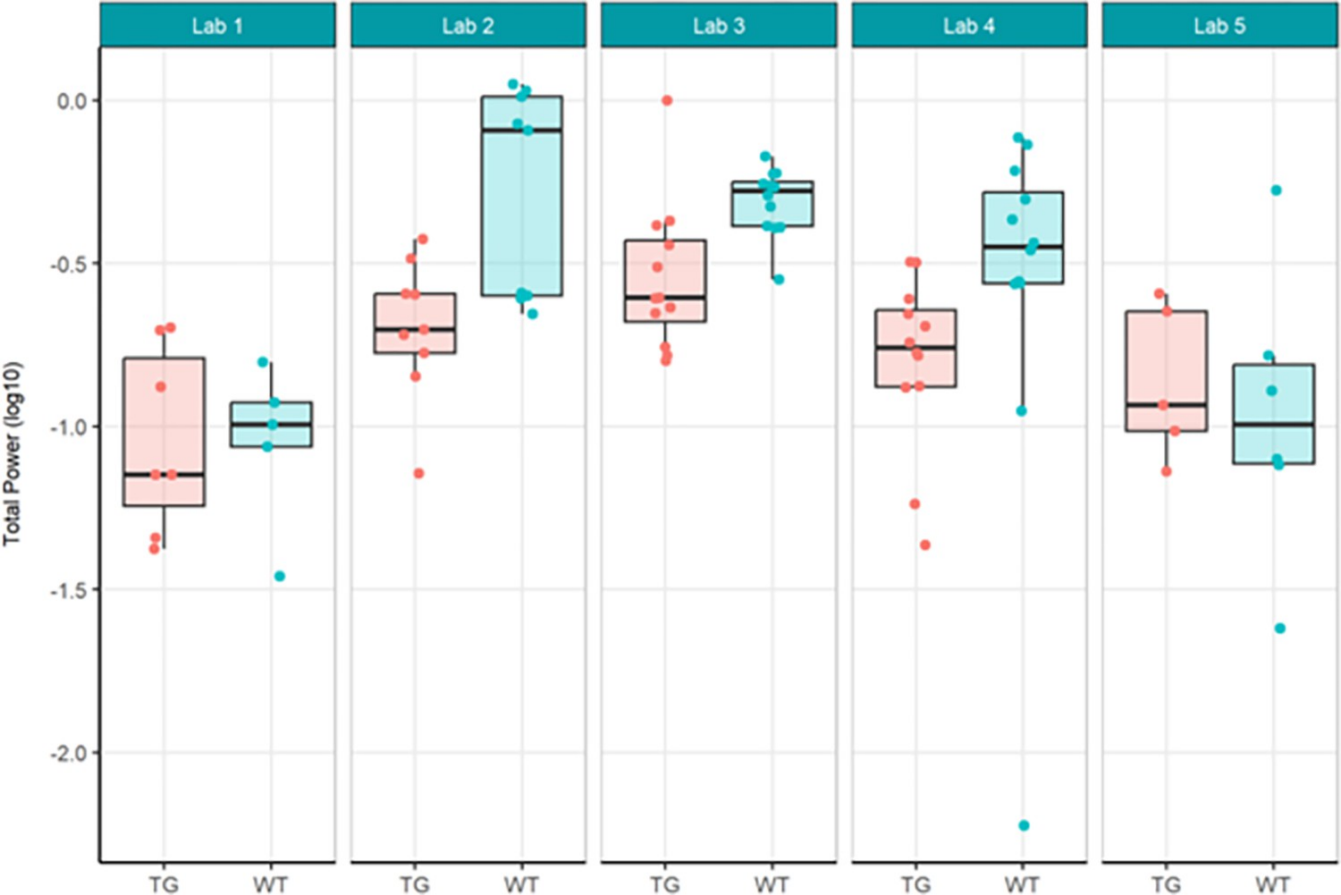
Harmonisation phase total power analysed locally by the partners. Tukey boxplots and individual data points for the log_10_ total power values per genotype group and every participating laboratory during phase 2 data collections. Lab 2 and Lab 3 found a significant decrease in total power in Tg4510 mice compared to WT controls (see S6 Table). The boxplot displays individual data points, as well as the median, the first (Q1) and third (Q3) quartiles and the whiskers are based on the interquartile range (IQR; Q3 –Q1) where they are not higher than Q3 + 1.5 * IQR and lower than Q1–1.5 * IQR.

**Fig 7:**
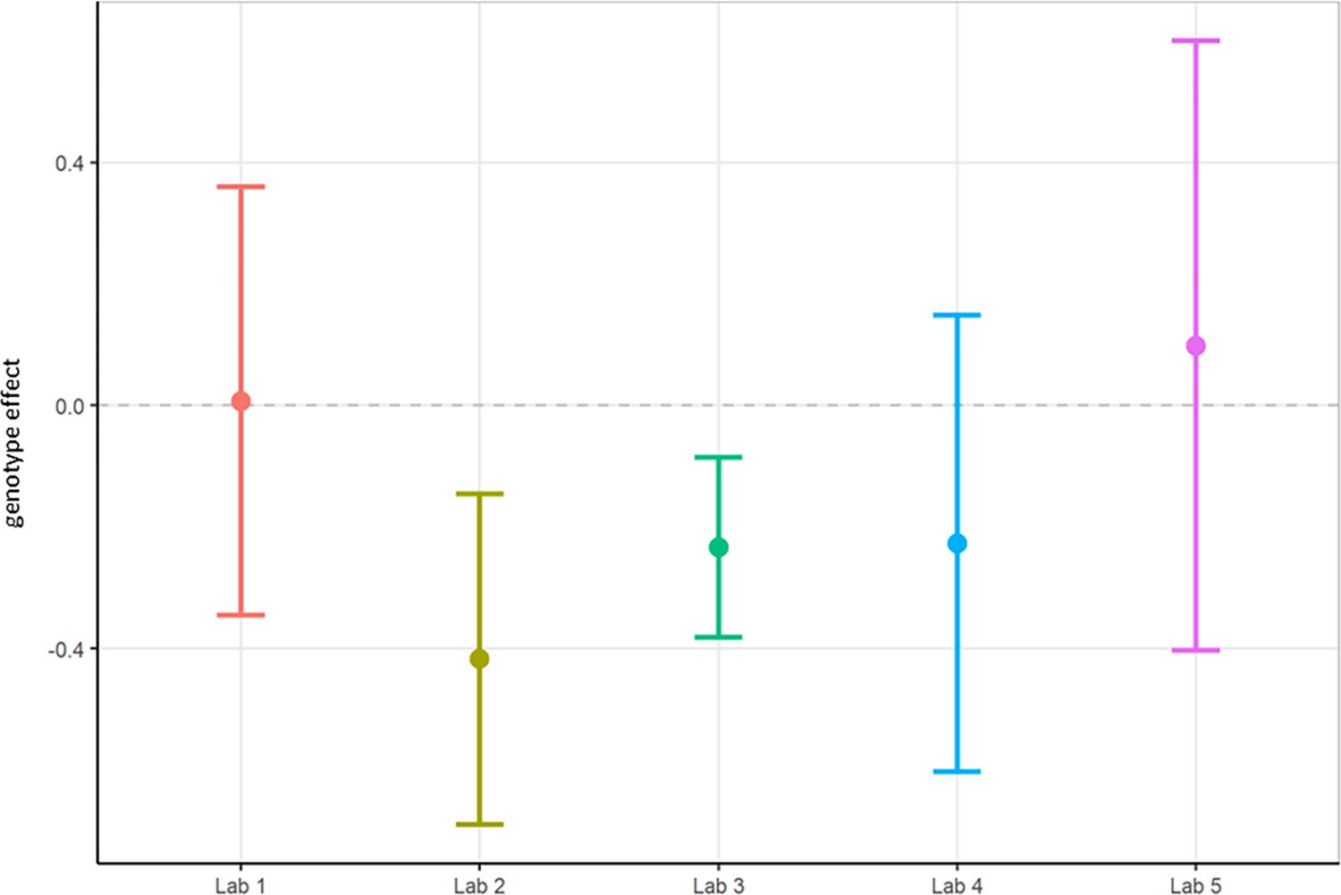
Harmonisation phase total power analysed locally by the partners. The figure shows estimated means of the genotypic TG-WT contrasts (“treatment effect”) for each the lower confidence limit (CL) and higher CL on log_10_ total power data.

First, total power was once again evaluated using local analysis on the data collected under the harmonised protocol (Figs 6 and 7; S6 Table). Statistical results between laboratories differed for total power, where Lab 2 and Lab 3 obtained a significant difference showing TG had reduced power compared to WT while the other laboratories did not replicate this genotype effect. The rate of successful replication of genotypic differences in total power was similar to the results obtained in phase 1, where also 2 out of 5 laboratories picked up on the expected total power decrease in the TG mice. Thus, despite the harmonisation efforts, outcomes could still not be aligned across all laboratories. Yet, the across-laboratories analysis comparing total power analysed locally under phase 1 (Figs 1 and 2) and the total power analysed locally collected under the harmonised protocol of phase 2 (Figs 6 and 7) showed a variance reduction that could be explained by the differing conditions per contributor from 27.8751 to 0.0607 (Table 1). Moreover, the TestgroupID:ContributorID interaction was estimated as 0.0029 (1.71%), which was very similar to the Localisation phase (0%). Finally, the residual variance changed minimally from approximately 0.0925 to 0.1039 but with the percentage increase from 0.33% to 62.05%, which was attributable to the large decrease of variance due to the contributor. Overall, this aligned with expectations. Residual variance was expected to remain the same across stage because the biological error was not supposed to be affected by the change in protocol (the experiments being the same, it should lead to the same biological error). However, the change in protocol had to coincide with a reduction in laboratory variability, and as observed, the harmonisation of experimental conditions and protocols did reduce the variance across laboratories. Nonetheless, we could not reach an alignment of the laboratories on the statistical conclusions in the differences between WT and TG.

**Fig 8:**
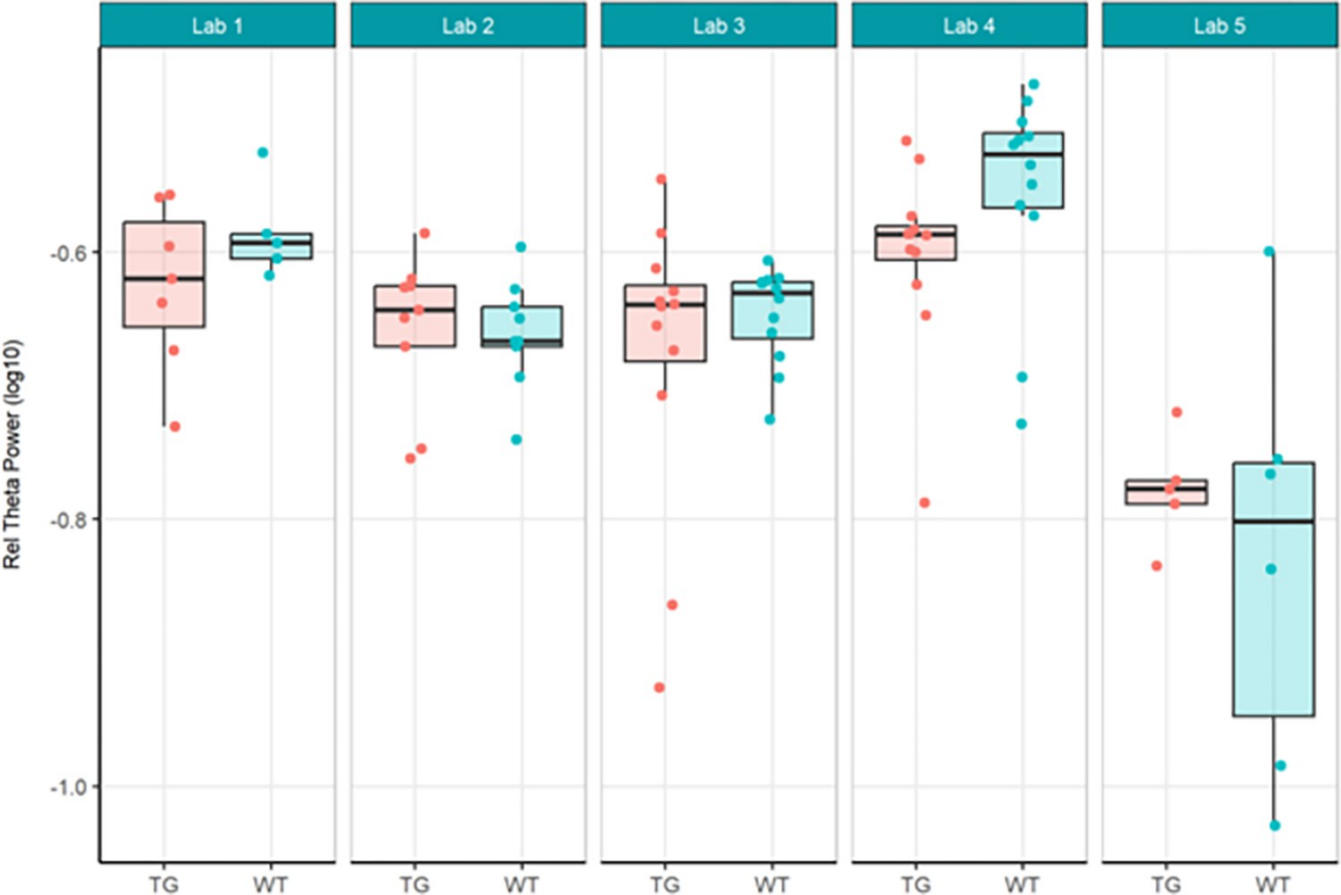
Harmonisation phase relative theta power analysed centrally. Tukey box-plots and individual data points for the log_10_ relative theta power values (obtained as a percentage of total power for individual subjects) per genotype group and every participating laboratory during phase 2 data collections. No laboratory found a significant difference in relative power for Tg4510 mice compared to WT controls. The boxplot displays individual data points, as well as the median, the first (Q1) and third (Q3) quartiles and the whiskers are based on the interquartile range (IQR; Q3 –Q1) where they are not higher than Q3 + 1.5 * IQR and lower than Q1–1.5 * IQR.

**Fig 9:**
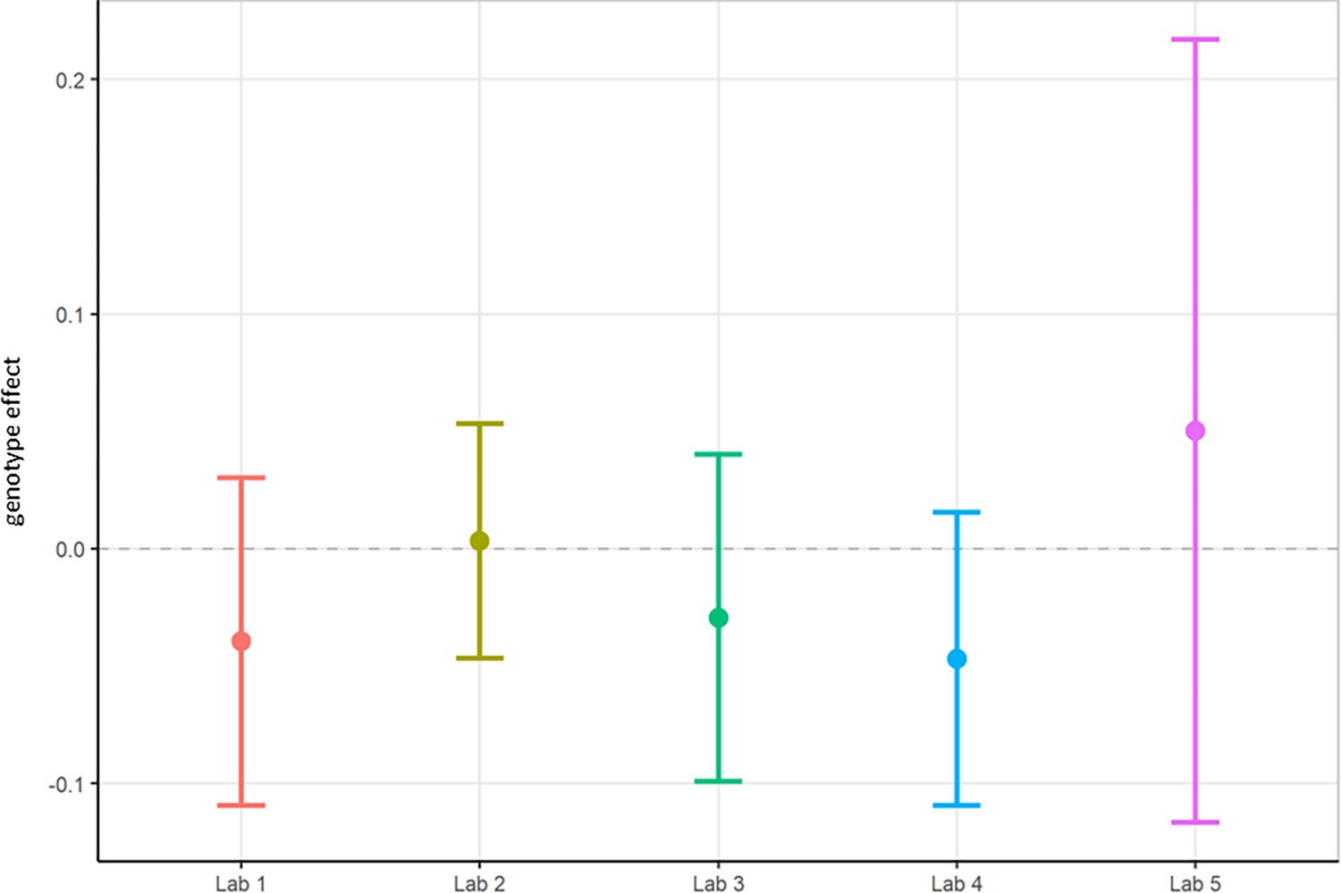
Harmonisation phase relative theta power analysed centrally. The figure shows estimated means of TG-WT contrasts with for each the lower confidence limit (CL) and higher CL on log_10_ relative theta power data.

**Fig 10:**
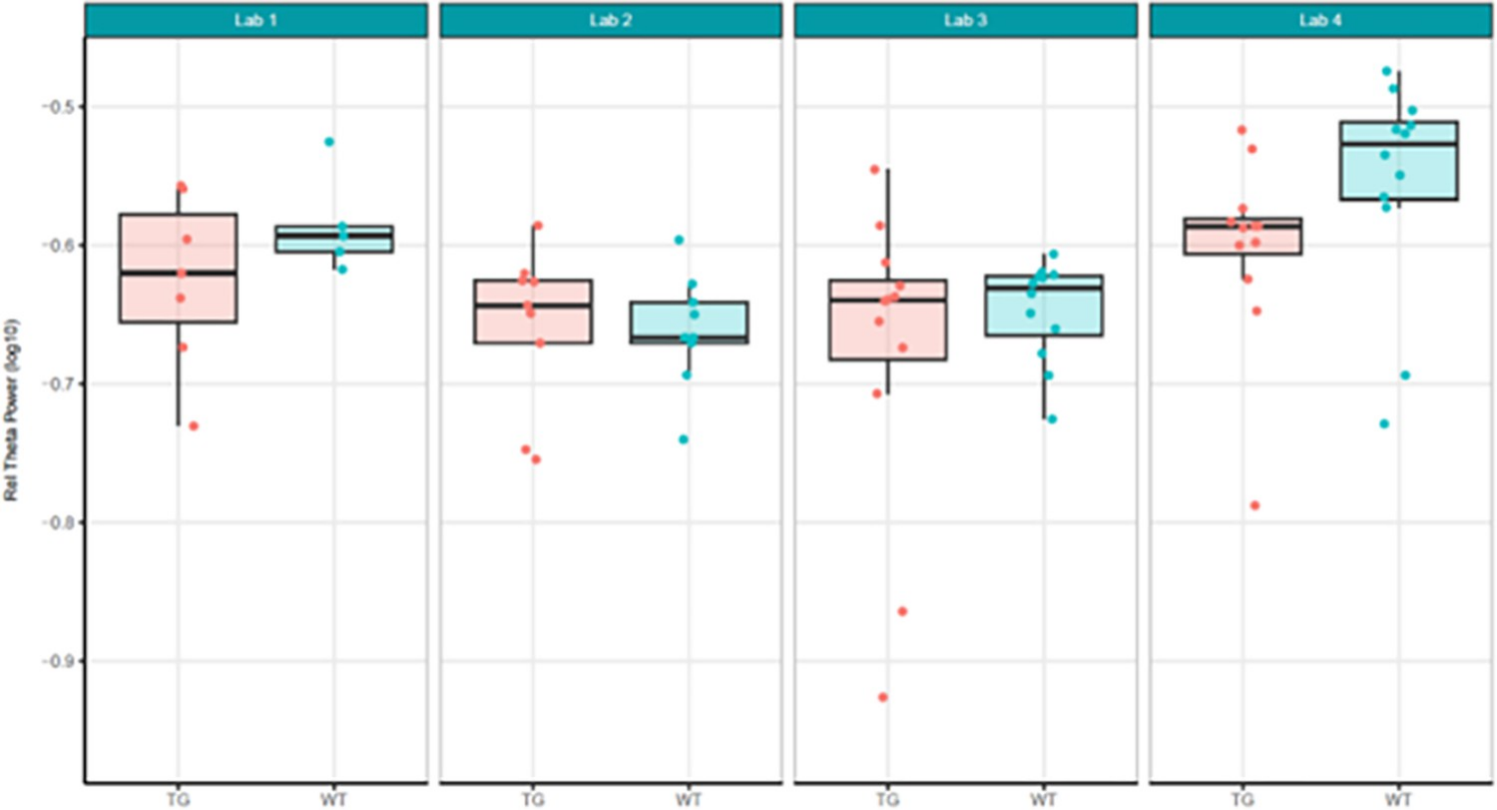
Harmonisation phase relative theta power analysed centrally with Lab 5 removed. Tukey box-plots and individual data points for the log_10_ relative theta power values (obtained as a percentage of total power for individual subjects) per genotype group and every participating laboratory during phase 2 data collections. The boxplot displays individual data points, as well as the median, the first (Q1) and third (Q3) quartiles and the whiskers are based on the interquartile range (IQR; Q3 –Q1) where they are not higher than Q3 + 1.5 * IQR and lower than Q1–1.5 * IQR.

**Fig 11:**
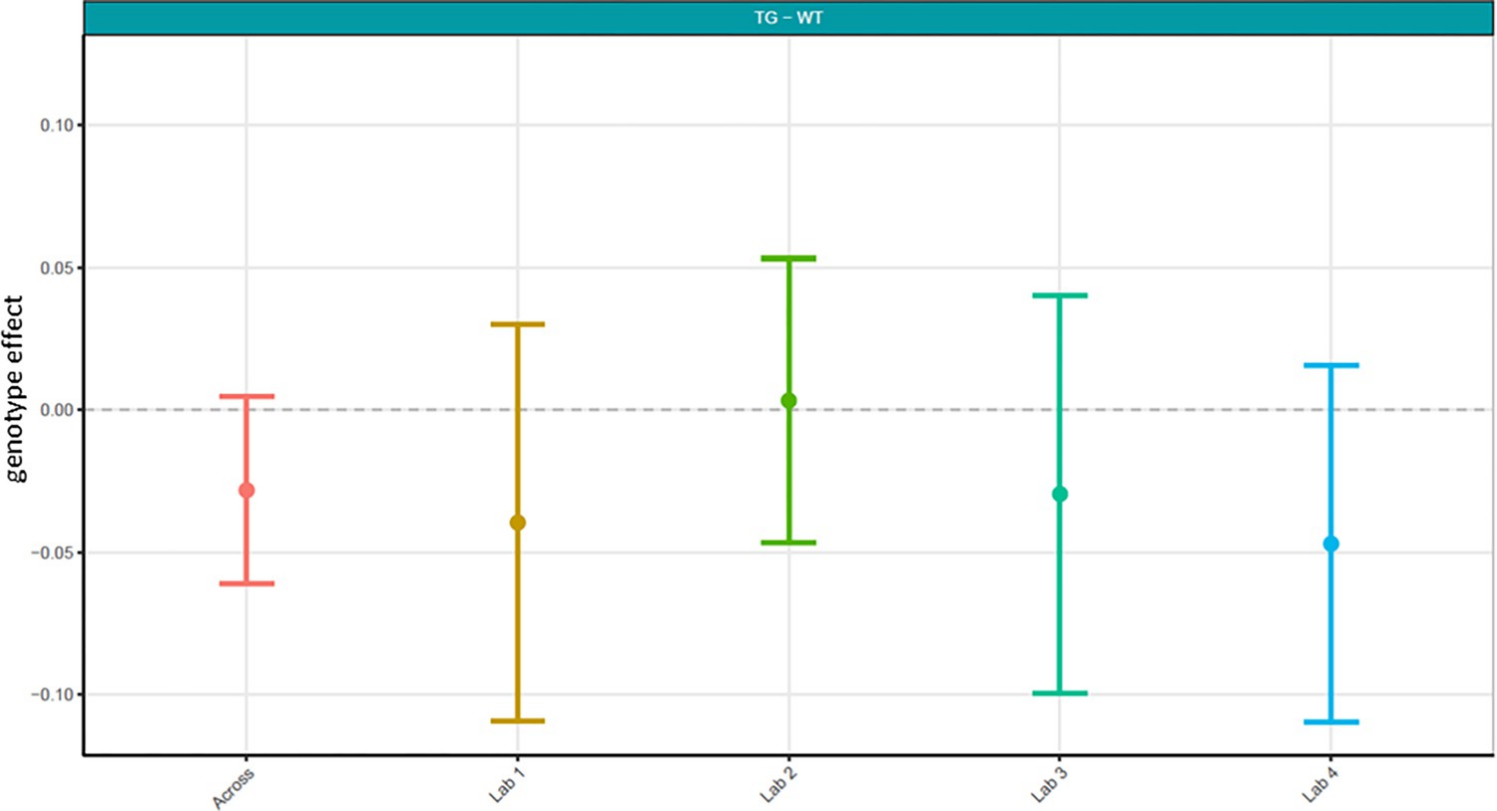
Harmonisation phase relative theta power analysed centrally with Lab 5 removed. The figure shows estimated means of TG-WT contrasts for each the lower confidence limit (CL) and higher CL on log_10_ relative theta power data for harmonisation phase 2 without Lab 5.

Next, because data differed considerably in phase 1, it was decided to not only have local analyses, but also to conduct a centralised analysis that ensured all phase 2 data was handled and processed identically. Here, we focused on relative theta power and compared phase 1 relative theta power analysed locally (Figs 3 and 4; S5 Table) with relative theta power collected under phase 2’s harmonised protocol analysed centrally (Figs 8 and 9; S7 Table). The centralised analysis of the data yielded results for the TG-WT comparisons that were more comparable across laboratories. The laboratories agreed that no statistical significance could be detected between the two test groups. Recall that in phase 1, one out of five laboratories found a difference between TG and WT. The across-laboratories analysis showed that ContributorID variance for relative theta power increased from 0.0037 (50.42%) in the Localisation phase to 0.0072 (54.17%) in the Harmonisation phase (Table 2). The TestgroupID:ContributorID interaction was estimated as 0, close to the value obtained in the Localisation phase (4.30%). The residual variance was similar from 0.0033 (45.28%) to 0.0059 (45.29%). Based on these facts, despite yielding more congruent statistical findings across laboratories, we conclude that harmonisation efforts and central analysis did not reach the anticipated reduction in laboratory-to-laboratory variance for relative theta power. However, after inspection of the data, it was clearly identified that this increase in laboratory-to-laboratory variance was due to Lab 5, which obtained results incongruent with the four other laboratories. Upon investigation into the potential source of variance within Lab 5, it was discovered that Lab 5 had used four different surgeons to comply with the timelines of the protocol that likely resulted in poorer EEG signal quality. Such a deviation from the harmonised protocol could certainly explain the variability seen in the data. Therefore, Lab 5 data set was excluded, and the analysis rerun to evaluate the impact on the results. It is evident when comparing results with Lab 5 (Figs 8 and 9) to without Lab 5 (Figs 10 and 11) that Lab 5 data were skewing the result (Table 3). The total variance decreased from 0.0131 to 0.0062. The residual variance changed from 0.0059 (45.29%) to 0.0047 (75.69%) without Lab 5, which demonstrated an improvement compared to that obtained in Localisation (0.0033 or 45.28%; Table 2). The variability due to the contributor went down from 0.0072 (54.71%) to 0.0015 (24.31%) without Lab 5 (Table 3). Comparing this variability to that obtained during Localisation (54.71%, Table 2), it is evident that for the Labs 1, 2, 3, and 4, (where no apparent divergence occurred), harmonisation efforts and central analysis helped considerably to standardize results for relative theta power.

**Table 3:** *Harmonisation phase relative theta power analysed centrally with Lab 5 data removed*. Table displaying the results from the between-laboratory analysis for phase 2 (Harmonisation) with and without Lab 5, displaying the laboratory-to-laboratory (ContributorID), treatment effect (TestgroupID:ContributorID), and residual variability.

Effect	Harmonisation with Lab 5 Variance Estimate (%)	Harmonisation without Lab 5 Variance Estimate (%)
ContributorID	0.0072 (54.71%)	0.0015 (24.31%)
TestgroupID:ContributorID	0.0000 (0.00%)	0.0000 (0.00%)
Residual	0.0059 (45.29%)	0.0047 (75.69%)
Total	0.0131 (100.00%)	0.0062 (100.00%)

In summary, relative theta power had significantly less variability (Tables 2 and 3) and statistical comparisons of TG-WT matched following not only harmonisation of the EEG protocol, but also centralised analysis (Figs 10 and 11) compared to localised protocols with local analyses (Figs 3 and 4).

### Phase 3: Ring-Testing

In phase 3 (Ring-Testing), blinded compound testing was introduced to the paradigm, and the data was both locally and centrally analysed to investigate the effects of central analysis further. An additional laboratory (Lab 6) contributed to this phase to test whether harmonisation (see S3 Table) would hold up when introducing a novel participant. These experiments were performed in C57BL/6J mice as the focus of interest shifted from a genotypic effect to a pharmacological effect on EEG (pharmaco-EEG). MK-801, a non-competitive NMDA receptor antagonist, was chosen for these pharmaco-EEG experiments as its drug effects in rodents have been well characterized as producing increased synchronization in gamma oscillations in a pharmacodynamic manner [53, 54]. Relative power was once again assessed. Additionally, gamma power was rendered as a percent change from within subject baseline to evaluate the influence of this processing on variance.

**Fig 12:**
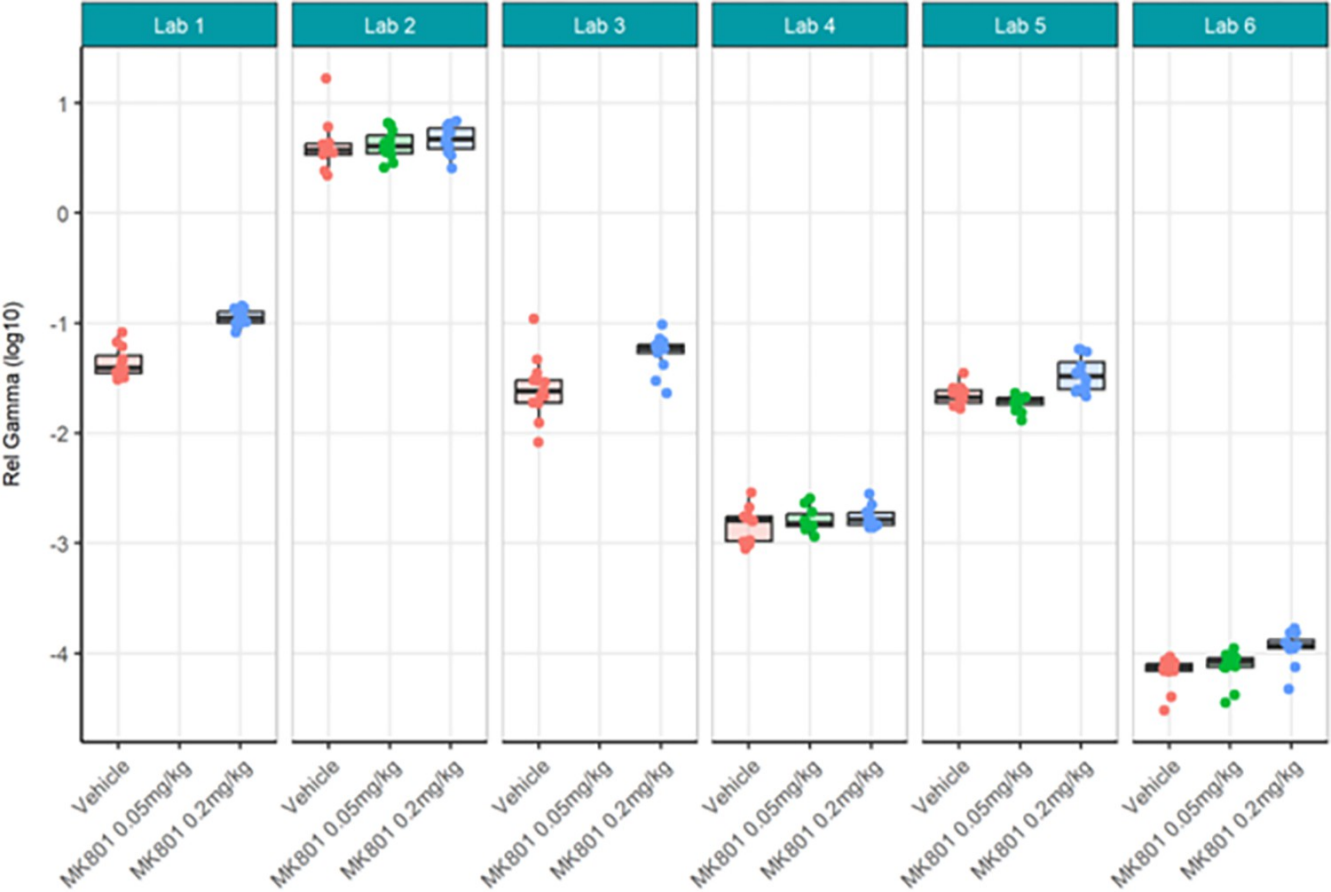
Ring-Testing phase relative gamma power analysed locally by the partners. Tukey boxplots and individual data points for the log_10_ relative gamma power per genotype and every participating laboratory during phase 3 data collections. Four of the 6 laboratories (Lab 1, Lab 3, Lab 5, and Lab 6) found a significant increase in relative gamma power following 0.2 mg/kg MK-801 compared to vehicle (see S8 Table). The boxplot displays individual data points, as well as the median, the first (Q1) and third (Q3) quartiles and the whiskers are based on the interquartile range (IQR; Q3 –Q1) where they are not higher than Q3 + 1.5 * IQR and lower than Q1–1.5 * IQR.

**Fig 13:**
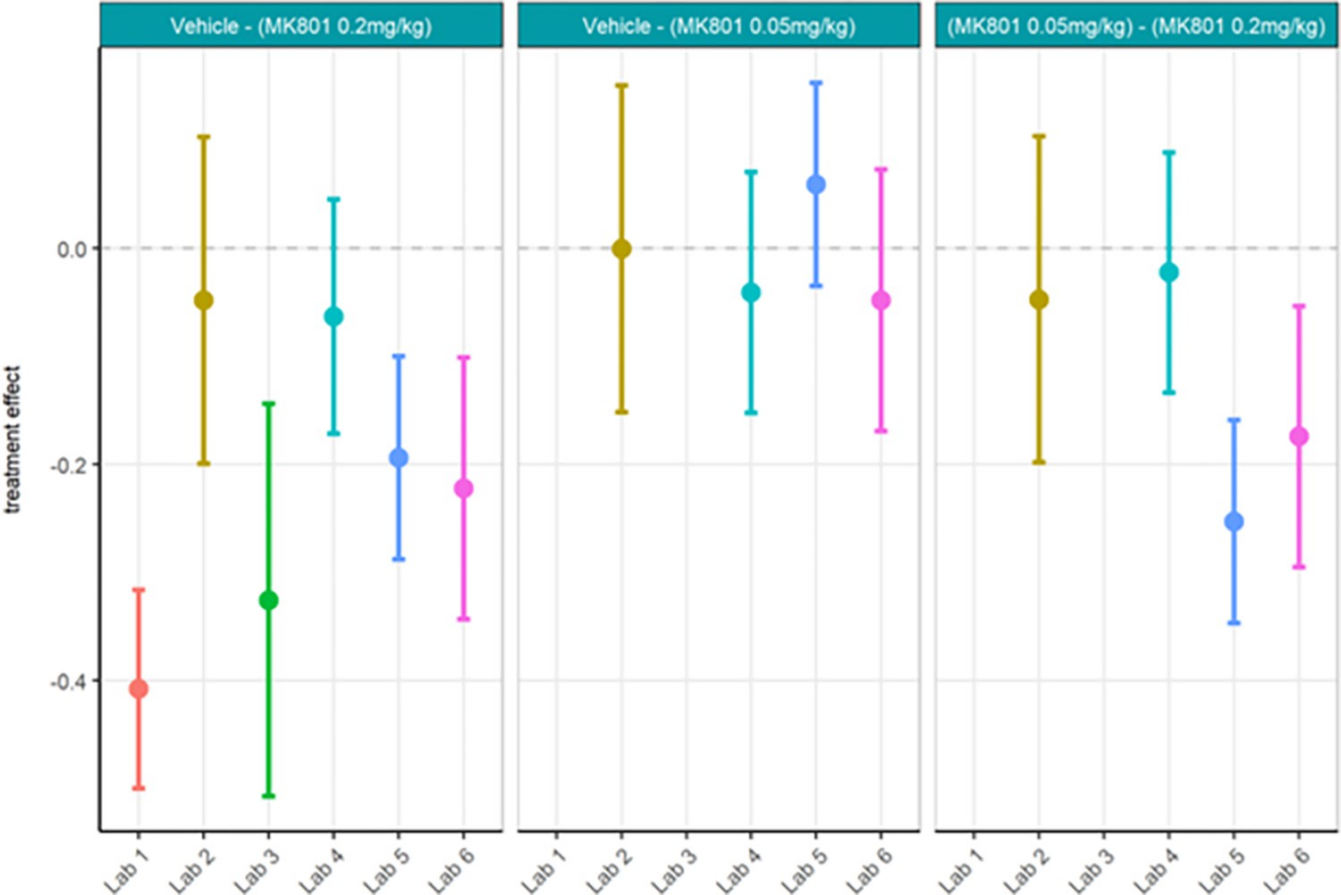
Ring-Testing phase relative gamma power analysed locally by the partners. The figure shows estimated means of treatment contrasts for each the lower confidence limit (CL) and higher CL on locally analysed log10 relative gamma power data.

**Fig 14:**
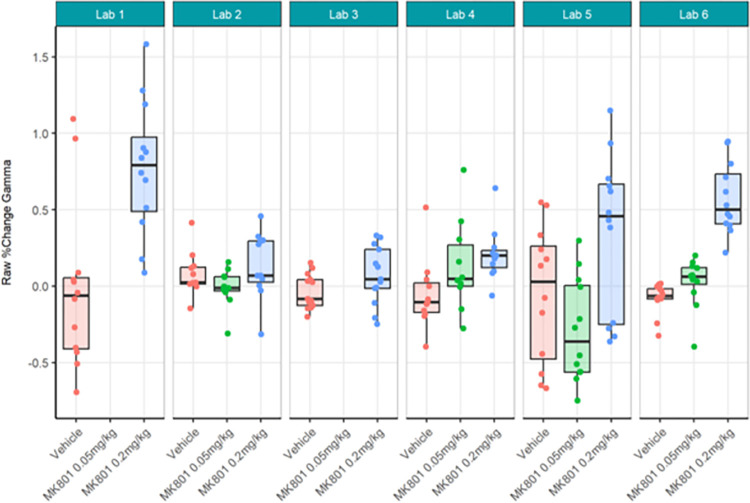
Ring-Testing phase gamma power as percent change from baseline using raw power analysed locally by the partners. The figure shows Tukey box-plots and individual data points for the raw gamma power percent change values per compound and every participating laboratory during phase 3 data collections. Four of the 6 laboratories (Lab 1, Lab 4, Lab 5, and Lab 6) found a significant increase in gamma power percent change following 0.2 mg/kg MK-801 compared to vehicle (see S9 Table). The boxplot displays individual data points, as well as the median, the first (Q1) and third (Q3) quartiles and the whiskers are based on the interquartile range (IQR; Q3 –Q1) where they are not higher than Q3 + 1.5 * IQR and lower than Q1–1.5 * IQR.

**Fig 15:**
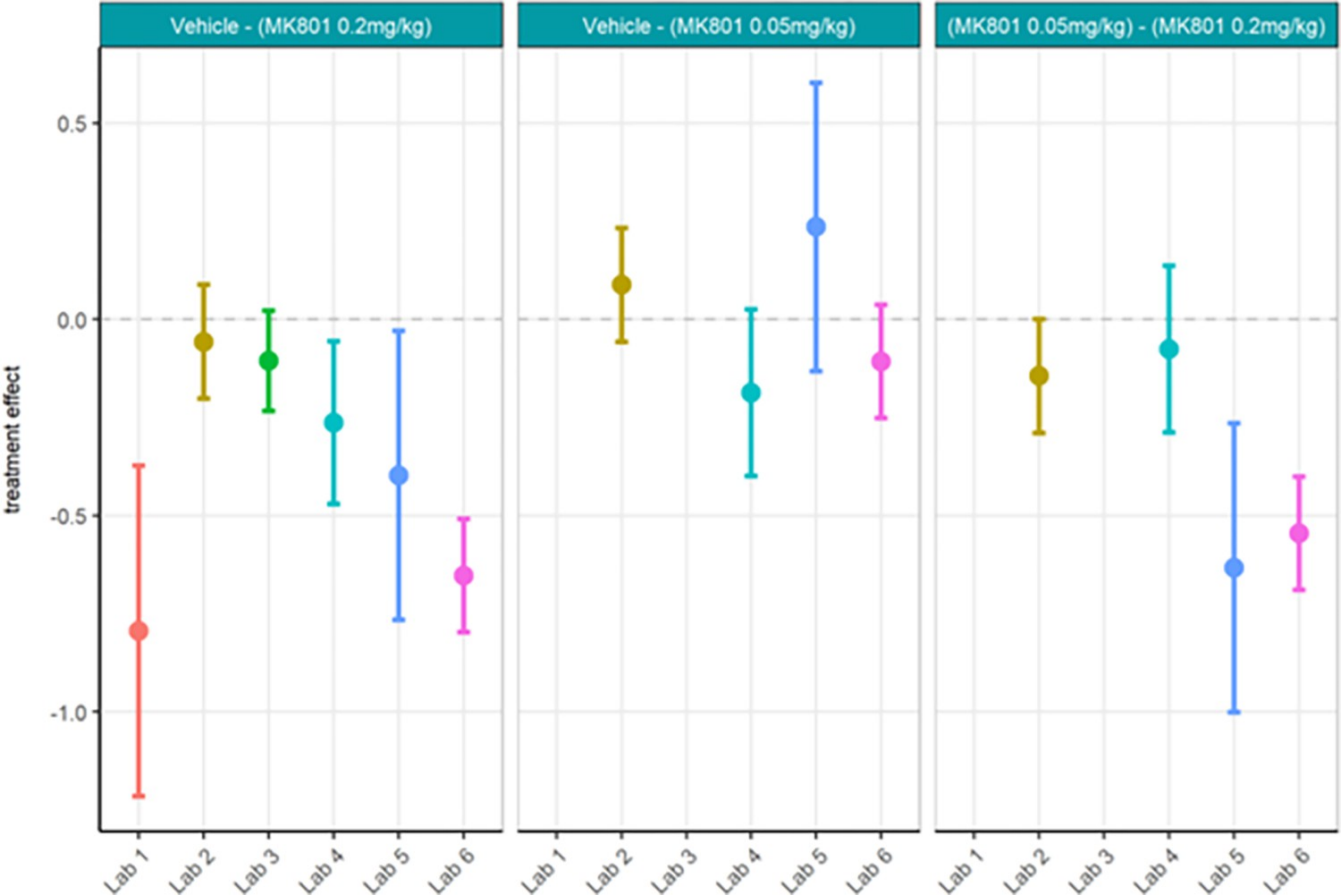
Ring-Testing phase gamma power as percent change from baseline using raw power analysed locally by the partners. The figure shows estimated means of pharmacological contrasts with for each the lower confidence limit (CL) and higher CL on raw gamma power percent change data.

First, the locally analysed results for the compound comparisons on relative gamma power were somewhat comparable across laboratories despite a wide range of actual values of relative gamma power (Figs 12 and 13; S8 Table) and considerably high contributor variance across laboratories (Table 4). Four out of six laboratories (Lab 1, 3, 5, and 6) agreed that a statistically significant relative gamma power increase could be shown between the vehicle and high dose (0.2 mg/kg) group. None of the laboratories found a difference between the vehicle and low dose (0.05 mg/kg) group. Two (Lab 5 and Lab 6) out of four laboratories found a relative gamma power increase between the high dose and low dose group. Interestingly, when comparing the locally analysed results obtained from gamma power as percent change from baseline using raw power (Figs 14 and 15; S9 Table), four (Labs 1, 4, 5, and 6) out of six laboratories detected a statistically significant relative gamma power increase between vehicle and the high dose group. Note, however, that only Labs 1, 5, and 6 were consistent in finding significant differences between these groups comparing relative power with percent change. Lab 3 identified a significant effect between vehicle and high dose when evaluating relative gamma power, but this effect was lost for raw gamma power percent change, while Lab 4 did not have a significant effect between vehicle and high dose when evaluating relative gamma power, but this effect was observed for raw gamma power percent change. Consistently, none of the laboratories found a difference between the vehicle and low dose group and two (Labs 5 and 6) out of four laboratories found an increase in gamma power between the high dose and low dose group. The changes in gamma power following MK-801 were more comparable across laboratories when evaluating percent change of raw gamma power, as evidenced in the low contributor variance (Table 5). Overall, both relative gamma power and raw gamma power percent change lead to largely comparable results across laboratories when analysed locally. Visibly the calculation method of the endpoint measure influenced the obtained statistical result.

**Table 4:** *Ring-testing phase relative gamma power analysed locally and centrally*. Table displaying the results from the across-laboratories analysis for relative gamma power in phase 3 (ring-testing) displaying the laboratory-to-laboratory variability (ContributorID), treatment effect variability between Labs (TestgroupID:ContributorID), and residual variability for both the locally and centrally analysed data.

Effect	Local relative gamma	Central relative gamma
ContributorID	2.5261 (98.88%)	0.0376 (51.00%)
TestgroupID:ContributorID	0.0056 (0.22%)	0.0074 (9.99%)
Residual	0.0231 (0.90%)	0.0287 (39.01%)
Total	2.5548 (100.00%)	0.0737 (100.00%)

**Table 5:** *Ring-testing phase gamma power as percent change from baseline using raw power analysed locally and centrally*. Table displaying the results from the across-laboratories analysis for raw gamma power percent change in phase 3 showing the variance of the locally analysed and centrally analysed data, displaying the laboratory-to-laboratory variability (ContributorID), treatment effect variability between Labs (TestgroupID:ContributorID), and residual variability.

Effect	Local	Central
ContributorID	0.0000 (0.00%)	0.0000 (0.00%)
TestgroupID:ContributorID	0.0296 (24.10%)	0.0199 (32.52%)
Residual	0.0933 (75.90%)	0.0412 (67.48%)
Total	0.1230 (100.00%)	0.0611 (100.00%)

**Fig 16:**
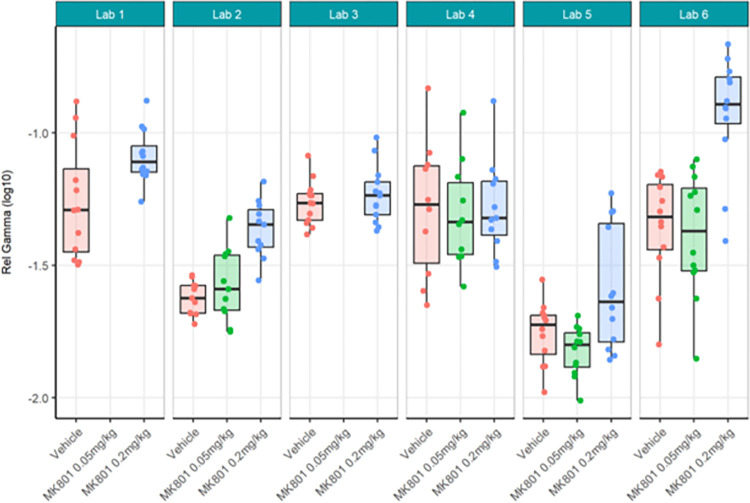
Ring-Testing phase relative gamma power analysed centrally. Tukey box-plots and individual data points for the log_10_ relative gamma power values per compound and every participating laboratory during phase 3 data collections. All displayed data were analysed centrally. Four of the 6 laboratories (Lab 1, Lab 2, Lab 5, and Lab 6) found a significant increase in relative gamma power following 0.2 mg/kg MK-801 compared to vehicle (see S10 Table). The boxplot displays individual data points, as well as the median, the first (Q1) and third (Q3) quartiles and the whiskers are based on the interquartile range (IQR; Q3 –Q1) where they are not higher than Q3 + 1.5 * IQR and lower than Q1–1.5 * IQR.

**Fig 17:**
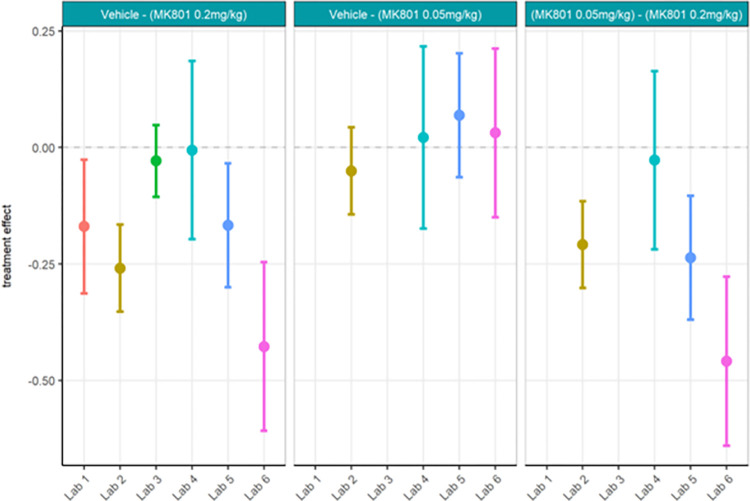
Ring-Testing phase relative gamma power analysed centrally. The figure shows estimated means of pharmacological contrasts for each the lower confidence limit (CL) and higher CL on log_10_ relative gamma power data.

Next, centralised data processing was conducted to compare with the localised analyses. Relative gamma power calculated centrally (Figs 16 and 17; S10 Table) yielded the same statistical findings as localised analysis only for Labs 1, 5 and 6. Lab 3 showed a significant difference between MK-801 (0.2 mg/kg) and vehicle in localised but not centralised analysis while Lab 2 showed a significant difference in centralised but not localised analysis. The across-laboratories analyses of the locally versus centrally analysed relative gamma power (Table 4) showed that the variance due to the contributor went from 2.5261 (98.88%) in the local analysis to 0.0376 (51%) in the central analysis. The TestgroupID:ContributorID interaction stayed similar with 0.0056 (0.22%) in the local analysis and 0.0074 (9.99%) in the central analysis, while the residual variance increased from 0.0231 (0.90%) in the local analysis to 0.0287 (39.01%) in the central analysis. We thus infer that central analysis of relative gamma provided more comparable results across laboratories, especially when considering the total variance decreased from 2.5548 to 0.0737. Thus, for relative gamma power, the centralised analysis of the data had a high impact on the comparability of endpoints in terms of variability. Regarding the statistical conclusions, no reliable improvement from local to central data analysis was observed. It surmises that confidence in the result would be obtained only if both local and central analyses yielded the significant difference while discarding incongruent findings.

**Fig 18:**
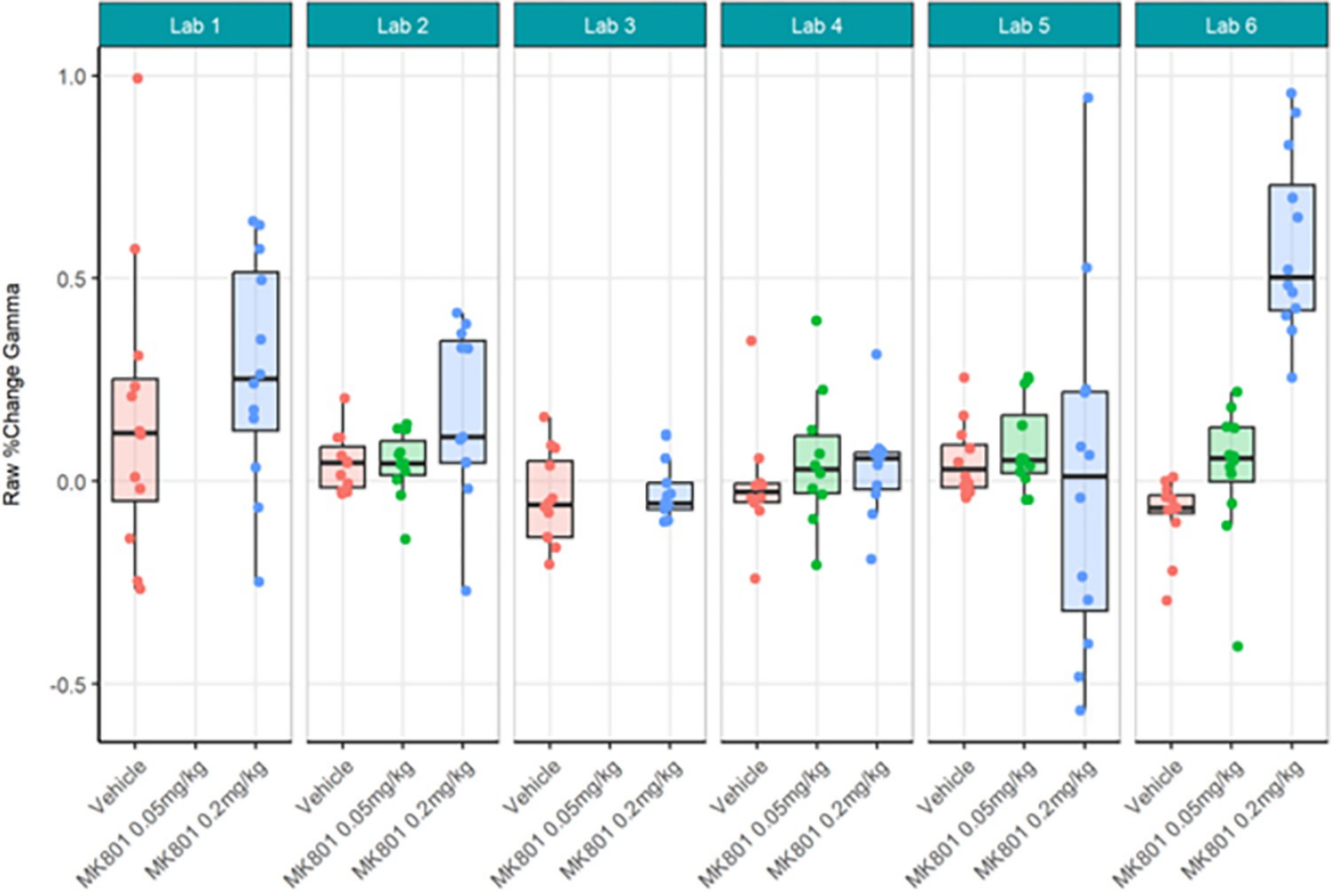
Ring-Testing phase gamma power as percent change from baseline using raw power analysed centrally. Tukey box-plots and individual data points for the raw gamma power percent change values per compound and every participating laboratory during phase 3 data collections. One of the 6 laboratories (Lab 6; Lab 2, p = 0.051) found a significant increase in relative gamma power following 0.2 mg/kg MK-801 compared to vehicle (see S11 Table). The boxplot displays individual data points, as well as the median, the first (Q1) and third (Q3) quartiles and the whiskers are based on the interquartile range (IQR; Q3 –Q1) where they are not higher than Q3 + 1.5 * IQR and lower than Q1–1.5 * IQR.

**Fig 19:**
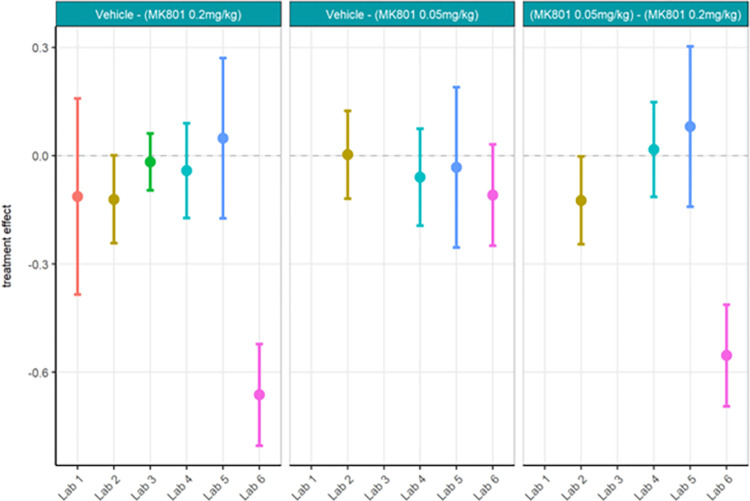
Ring-Testing phase gamma power as percent change from baseline using raw power analysed centrally. The figure shows estimated means of pharmacological contrasts with for each the lower confidence limit (CL) and higher CL on log_10_ raw gamma power percent change data.

Strikingly, gamma power as calculated centrally as percent change from baseline using raw power (Figs 18 and 19; S11 Table) yielded quite different statistical findings to localised analyses. Only Lab 6 had significance across analyses. Labs 1, 4, and 5 showed a significant difference between MK-801 (0.2 mg/kg) and vehicle in localised but not centralised analyses while Lab 2, again, showed a significant difference in centralised but not localised analyses. These findings contrast with the observed similarities between the data (Figs 14 and 18) and may in part be due to the nature of the statistical comparisons (simple linear regression fitted to the different responses by laboratory with Test Group as unique fixed effect). Meanwhile, the across-laboratories analyses showed that the variance was similar for the local versus central analyses of gamma power as percent change from baseline (Table 5) suggesting that this endpoint may not benefit much from centralized analyses. In fact, this suggests that when normalizing each recording by itself using a percent change from baseline, the variance is inherently reduced across all recordings and therefore across laboratories.

It was expected that the Ring-Testing phase would provide lower laboratory-to-laboratory variance (ContributorID, TestgroupID:Contributor ID) than the Localisation phase as a harmonised protocol (developed for the Harmonisation phase) and centralised analysis were used, while having similar biological variances (Residual). This was indeed the case when comparing relative gamma power as the centralised analysis greatly reduced across-laboratory variance and likely correctly identified the significant differences between MK-801 (0.2 mg/kg) and vehicle conditions. Interestingly, as discussed above, the percent change from baseline endpoint did not benefit greatly from centralised analysis likely because it normalises each recording to itself. That significant findings for comparisons between MK-801 (0.2 mg/kg) and vehicle groups differed across all four analyses on the same dataset strongly emphasises that specific individual analysis steps implemented locally can greatly influence the result.

## Conclusions

The concerns about replicability issues require detailed investigation of its contributing factors. Lack of replicability leads to inefficient use of resources and has financial and ethical implications, especially in the case of *in vivo* research [11, 12]. Ultimately, low replicability rates slow down knowledge gain, and therefore impact the speed of drug development. This incentivized the set-up of the EQIPD consortium which aimed to identify causes of replicability failures in preclinical research and to develop harmonisation strategies for early drug discovery research. The present study was carried out in three phases: Localisation, Harmonisation, and Ring-Testing. Each phase was executed as a part of Work Package 4 (WP4) of EQIPD, which aimed at validation of the principles, strategies, and research models developed to improve robustness and data quality in research and preclinical studies with an initial focus on neuroscience and safety. The goal of the Localisation phase was to identify factors that influence between-laboratory variability of routine preclinical EEG experiments. Based on the outcomes, factors deemed most important were chosen for harmonisation in the second phase of experiments to test the hypothesis that between-laboratory variability would be reduced because of such unification of procedures. Thus, in the Harmonisation phase a partially standardised protocol was created, more study-related factors (excluding hardware) were unified, and the data analysis was centralised. In the third and final Ring-Testing phase, a reference compound was introduced into the qEEG set-up to verify the ability of the harmonised protocol to generate consistent results in a blinded context and to further compare the effects of centralised analysis to local analyses. Our main findings show that in the Localisation phase, in which participating sites administered their own laboratory-specific protocols, large across-laboratory differences in the outcome measures were observed. Due to the harmonisation efforts and centralised analysis, findings obtained in the Harmonisation phase became more aligned as the total across-laboratory variance significantly decreased for the investigated parameters. Moreover, the Ring-Test phase results showed further reductions in between-laboratory variability.

We deduce from these findings that a critically important step for reproducibility of EEG recordings is to align study design and data analysis including pre-processing steps such as artifact rejection, FFT smoothing algorithm, and other data-processing and normalization factors. Recently, a replicability study showed that the choice of analysis software can influence the obtained raw voltage values, which is also a constant in the central analysis [55] (for video analysis, see [56]). Therefore, we recommend centralising analyses in any multicenter study to achieve better comparability between laboratories.

### Localisation phase

Data collected during the Localisation phase revealed a large between-laboratory variability in outcome measures almost completely due to participant-specific parameters of conducting and analysing their qEEG studies. Results provided by the sites differed so much that raw data presentation and further interpretation were not possible without log-scale transformation. Further, not all outcomes agreed on the statistical findings for TG-WT in the endpoints (total power, relative power of theta band) evaluated. In search for the sources of this variability, participants reported and analysed experimental conditions regarding the following domains: animals and their husbandry, facility, housing and recording conditions, surgery, EEG recording parameters, hardware and data analysis and software (see S1 Table) with no definite result.

One of the most important aspects of successful qEEG experiments is the stereotaxic surgery of electrode implantation [57]. We believe this to be the leading cause for the high between-laboratory variability observed since recommendations for preclinical surgical preparation were very general leaving considerable freedom for onsite adaptation. Given how many surgery-related factors may contribute to the quality of the signal and further impact on data analysis [58] it was expected that data from Localisation would be highly variable because each laboratory determined independently how to run the study (e.g. housing of animals, care and husbandry provided, surgical and post-operative procedures). Although participating sites used the same type of anaesthesia (inhalation isoflurane), differences were noted for usage of local anaesthetic, analgesic, supplementary fluid administration, and the duration of the surgery per animal and across surgeons. This should be scrutinized as inadequate procedures are persistent issues associated with experimental intracranial surgery underscoring the need for extensive training of those working with laboratory rodents subjected to craniotomies [59]. Moreover, since the EQIPD consortium gave an invaluable opportunity to young researchers, doctoral students and post-doctorates, to familiarize with academic and industrial research environments, experimental work was executed mostly by the researchers at an early stage of their scientific career. Many of these junior partners required extensive training on surgical techniques with those who participated in all phases of the project showing great improvement. We feel strongly that this was a confounding factor influencing the between-laboratory variability. To illustrate the point, after completion of the Localisation phase mice brains were subjected to histopathological examination (by M.J.). In some of the brain samples, coming from different laboratories, lesions in the cortex underlying the electrode positions were detected. Such tissue damage can result from incorrect screw insertion (possibly due to improper screw length or type, wrong drill size) that exceeds the skull depth and presses into the dura mater. This kind of damage indicates an inexperienced surgeon. If the dura mater and the cortex are injured, scar tissue (caused by neuroinflammation and gliosis) form around the electrode and may alter electrode impedance and EEG quality and have an impact on subsequent recordings even after extended recovery periods [60–62]. In all other studies one must exclude these animals from the analyses. They were deliberately kept in here to explore the variance that improper electrode positioning may cause.

Notwithstanding tissue distortion, other variables such as short-circuiting of and/or fragile connections of current paths due to surgical procedures or electrode and/or headmount design may account for increased variance (see [63]). Importantly, the EEG performed in rodents reported in the literature is often more aligned with electrocorticography (ECoG) where electrodes are placed directly on the exposed surface of the brain. EcoG has higher temporal and spatial resolution than scalp EEG as it does not suffer from the attenuation of signal by skull and scalp and has a significantly better signal-to-noise ratio than scalp EEG. This is the main reason why in rodent EEG studies screws are positioned into or through the skull. The best EEG (EcoG) electrode implants only contact dura and do not make any damage. In fact, it is better to not damage the brain or induce any bleeding when inserting an electrode of any type as it is sufficient to collect quality data by insertion into the skull alone. This avoids gliosis from occurring around the electrode or within the damaged tissue that would dampen the recording (reduce signal-to-noise) by increasing the overall impedance [64].

Equipment and data acquisition as well as data analysis also contribute to between-laboratory variability. Availability of various recording systems (e.g., tethered or wireless / telemetry, see [65]), analysis software (dedicated to EEG analysis ready-to-use with limited parameters or fully programmable, custom made) and the very general recommendations leave considerable freedom in obtaining and analysing preclinical EEG recordings. Although participating sites were asked to provide as much information about data analysis as possible, some of the commercially available hardware and software provide insufficient information about their intrinsic mode of operation, and very often users are simply not aware of e.g., the build-in filters. Once recorded, the quality of the signal should be physiologically assessed and if satisfactory, non-brain-related artifacts should be removed in a pre-processing step. There are multiple methods for the pre-processing of the data [66]. Also, the choice of software can change the absolute voltage measured [55] and this can impact the comparability of results across laboratories. Artifacts may be filtered out or simply removed manually, data then interpolated or the whole epochs removed, which happened to the datasets provided by some participants. Indeed, this influenced the size of the resulting data pool and may obviously lead to variable results. Another source of variability lies in the processing of the cleaned EEG signal. Laboratories were asked to provide the power for the different frequency bands, which is usually done by means of FFT. However, a choice of a particular windowing technique in the FFT procedure may influence the results as well [67]. The full ramification of signal processing and its effect on spectral content is outside the scope of this paper but readers are encouraged to review the significant works of Dr. Erol Başar, reviewed by [68], as well as fundamentals of EEG signal processing [69].

Of course, other factors could be contributing to the variance in findings, including the mouse model itself. Further considerations to be aware when embarking on research using a heritable mouse model include the health status of the mouse colony and presence of previous illness or infection, husbandry practices, the environment in which the mice are maintained, and the breeding history of the colony to ensure adequate maintenance of the colony to avoid genetic drift or inbreeding depression [70].

### Harmonisation phase

As shown in Localisation, participating sites were asked to provide information regarding animals and their husbandry, facility, housing and recording conditions, surgery, EEG recording parameters, hardware and data analysis and software (see S2 Table). Based on the outcome of tabulating the various laboratory-specific conditions across these parameters, a harmonised protocol was created that standardised, where feasible, crucial elements of the qEEG experiment. Despite this effort, still some degree of inconsistencies was noted in all the domains in Harmonisation and further in Ring-Testing. In general, efforts made to unify the procedures resulted, as a priori hypothesised, in reduced variability across participating sites for all EEG endpoints evaluated. However, it should be emphasised that the positive results regarding relative theta were obtained only after excluding one laboratory from the analyses. This laboratory showed suspiciously large variability in the data set. A decision for treating the data with caution was made only after critical analysis of the possible underpinnings, such as multiple surgeons performing stereotaxic surgery, issues with recovery from anaesthesia, and signal quality issues. These are valid reasons known to cause considerable variability and deemed legitimate for removal of this dataset in a separate, exploratory analysis. Nevertheless, analyses with and without this data set are reported to highlight and again emphasize the message that only high-quality EEG signals should be properly evaluated. The importance of the quality of surgery was also underlined since it was clearly demonstrated that the more surgeons were involved, the higher the variability of the results. Notwithstanding that specific theta power-related variability might be related to the fact that the theta power in rodents is higher during wakefulness and REM sleep [71] and sleep stages were not controlled for in these experiments. Although sleep-specific qEEG was deemed outside the scope of these studies, it should be noted that effective translation of pharmaco-EEG relies on defining the different substages of sleep in a specific manner and evaluating the spectral changes within those bands [24–26, 39, 40] and differences in sleep patterns across the participating laboratories may be an additional source of variability for the theta outcome, which was not herein controlled for.

Movement artifacts, occurring almost exclusively in the wake state, are usually the first phenomena filtered or excluded during the data pre-processing. As shown previously, this stage of data analysis is highly variable (e.g., milliseconds-lasting artifacts can be removed and the data extrapolated or, the whole epochs of time during the artifacts can be excluded). Compromised surgery quality or improper tethering or animals’ chewing of tethers would likely result in more artifacts, which in turn may lead to more variability in the data set. Simply put, it is bad practice to analyse bad quality data and experienced electrophysiologists understand this and will report data missing and why it is missing. Finally, we cannot emphasize enough how this exercise points to scientific integrity as a very important aspect of work published. Honesty in admitting a potential failure and highlighting fundamental challenges in conducting the study saved an overall result and it can be conveyed here as it happened for inclusivity.

The expectation was that alignment across laboratory findings would have improved after harmonisation and centralised analysis, but in fact it is difficult to draw conclusions on the total power decrease seen in the Tg4510 model in 2 of the 5 laboratories. The alignment on results did improve for relative theta power, showing no effect across all laboratories. In general, results from the Harmonisation phase showed that minimal harmonisation and centralized data analysis was sufficient to increase replicability without the need for rigorous standardisation on equipment or housing conditions, yet with only modest results. To further investigate how much influence centralizing the analysis has on the comparability between laboratories, we did a local and central analysis during the Ring-Testing phase.

### Ring-Testing phase

In the Ring-Testing phase the robustness of the harmonised protocol was tested in a blinded compound (MK-801) context and the importance of central analysis was further verified in comparison with the local analysis done by the partners. Similar harmonisation steps were taken where possible (see S3 Table). Overall, we conclude that centralised data analysis accounted for the highest decrease in between-laboratory variability, especially for the endpoint relative gamma power. To demonstrate how analyses can greatly influence study variation, we point out that the total variance went from 2.5548 for relative gamma power (local analysis) to 0.1230 for gamma power as a percent change from baseline (local analysis). The centralised analysis of gamma power as a percent change from baseline decreased the variance even further to 0.0611. This reduction in variance provides compelling evidence that the normalisation applied by taking a percent change compensates greatly for variability between laboratories. As a final observation on how harmonisation efforts reduce variance, we focused on comparing the ContributorID variance for Localisation’s total power analysed locally, which was 99.67%, to that of Ring-Testing’s percent change in gamma power analysed locally, which was 0.00%. But although we attained a clear reduction in variance by implementing best practices aligned across participants, replicability of drug effects remains an unresolved issue.

In summary, results obtained in this multicentre preclinical qEEG study confirmed the complex nature of this type of experiment starting from the surgery and data collection through data pre-processing to data analysis that ultimately influenced the results. Harmonisation of protocols and centralized data analysis, seem to be crucial in reducing laboratory-to-laboratory variability. We recommend reporting in any manuscript detailed documentation about the methodology (animals, surgery, electrode types, recording equipment models and settings, data analysis software and settings), to encourage harmonisation where possible, but also so that if replication fails, a greater understanding of why may be ascertained. Extensive in-depth training for new researchers in the field of EEG should not only include stereotaxic surgeries but extend to a fundamental understanding of neurophysiological or biosignal generation, electronics, and signal processing including the ability to visually discriminate artifacts from high quality raw signals. Guidelines for preclinical EEG should be updated and adhered to, aligned with previous intentions [24–26, 72, 73]. Regarding data itself, it would be ideal to make raw data available for public use, particularly as new analytical capabilities, including machine learning [74] and automated artificial intelligence [75] emerge. Finally, a comparison of this paper is made to the complexity and lack of standardization that has hampered utility of event-related potentials and qEEG biomarkers in clinical drug development [76] where standardisation of instrumentation and methods has improved test-retest reliability [28]. We highlight the need for the field of preclinical and by extension clinical EEG to better align on outcome measures used in these studies as between-laboratory variability within this manuscript is likely a proxy for similar studies in the literature.

## Supporting information

S1 Table Experimental parameters across laboratories during the Localisation phase. The table provides information regarding animals and the facility, husbandry and housing, surgical procedures and care, EEG recording conditions and parameters, hardware and software, data analysis methods and experimental procedures.
(PDF)

S2 Table Experimental parameters across laboratories during the Harmonisation phase. The table provides information regarding animals and the facility, husbandry and housing, surgical procedures and care, EEG recording conditions and parameters, hardware and software, data analysis methods and experimental procedures.
(PDF)

S3 Table Experimental parameters across laboratories during the Ring Testing phase. The table provides information regarding animals and the facility, husbandry and housing, surgical procedures and care, EEG recording conditions and parameters, hardware and software, data analysis methods and experimental procedures.
(PDF)

S4 Table Localisation phase total power analysed locally by the partners. The table shows estimated means, standard error, lower confidence limit (CL), and upper confidence limit (CL) of WT and TG groups, as well as their contrasts (TG-WT). The p-value was derived from the statistical models run per laboratory on log10 total power data. Note that p-values are not provided for individual means as this was not of interest in this study.
(PDF)

S5 Table Localisation phase relative theta power analysed locally by the partners. The table shows estimated means, standard error, lower confidence limit (CL), and upper confidence limit (CL) of WT and TG groups, as well as their contrasts (TG-WT). The p-value was derived from the statistical models run per laboratory on log10 relative theta power data. Note that p-values are not provided for individual means as this was not of interest in this study.
(PDF)

S6 Table Harmonisation phase total power analysed locally by the partners. The table shows estimated means, standard error, lower confidence limit (CL), and upper confidence limit (CL) of WT and TG groups, as well as their contrasts (TG-WT). The p-value was derived from the statistical models run per laboratory on log_10_ total power data. Note that p-values are not provided for individual means as this was not of interest in this study.
(PDF)

S7 Table Harmonisation phase relative theta power analysed centrally. The table shows estimated means of WT and TG groups as well as their contrast, with each the standard error, lower confidence limit (CL), upper confidence limit (CL), and p-value derived from the statistical models run per laboratory on log_10_ relative theta power data. Note that p-values are not provided for individual means as not of interest in this study.
(PDF)

S8 Table Ring-Testing phase relative gamma power analysed locally by the partners. The table shows estimated means, standard error, lower confidence limit (CL), and upper confidence limit (CL) of pharmacological interventions and their contrasts. The p-value was derived from the statistical models run per laboratory on log_10_ relative gamma power data. Note that p-values are not provided for individual means as this was not of interest in this study.
(PDF)

S9 Table Ring-Testing phase gamma power as percent change from baseline using raw power analysed locally by the partners. The table shows estimated means, standard error, lower confidence limit (CL), and upper confidence limit (CL) of pharmacological interventions and their contrasts. The p-value was derived from the statistical models run per laboratory on log_10_ raw gamma power percent change data. Note that p-values are not provided for individual means as this was not of interest in this study.
(PDF)

S10 Table Ring-Testing phase relative gamma power analysed centrally. The table shows estimated means, standard error, lower confidence limit (CL), and upper confidence limit (CL) of pharmacological interventions and their contrasts. The p-value was derived from the statistical models run per laboratory on log_10_ relative gamma power data. Note that p-values are not provided for individual means as this was not of interest in this study.
(PDF)

S11 Table Ring-Testing phase gamma power as percent change from baseline using raw power analysed centrally. The table shows estimated means, standard error, lower confidence limit (CL), and upper confidence limit (CL) of pharmacological interventions and their contrasts. The p-value was derived from the statistical models run per laboratory on raw gamma power percent change data. Note that p-values are not provided for individual means as this was not of interest in this study.
(PDF)

S1 Fig Ring-Testing phase power across all frequency bands as absolute (raw), relative, and percent change from baseline using raw power analysed centrally. The figures show estimated means (bars) and individual data points for absolute power, relative power, or percent change from baseline calculated using absolute power across all frequency bands for each treatment group (vehicle, MK-801 at 0.05 and 0.2 mg/kg) when tested during the Ring-Testing Phase by Laboratory. All data were analysed centrally. No statistical comparisons were performed on these data.
(PDF)

## References

[1] Begley CG, Ellis LM. Drug development: Raise standards for preclinical cancer research. Nature. 2012 Mar 28;483(7391):531–3. doi: 10.1038/483531a . 22460880

[2] Begley CG, Ioannidis JP. Reproducibility in science: improving the standard for basic and preclinical research. Circ Res. 2015 Jan 2;116(1):116–26. doi: 10.1161/CIRCRESAHA.114.303819 . 25552691

[3] Freedman LP, Cockburn IM, Simcoe TS. The Economics of Reproducibility in Preclinical Research. PloS Biol. 2015 Jun 9;13(6):e1002165. doi: 10.1371/journal.pbio.1002165 Erratum in: Biol PloS. 2018 Apr 10;16(4):e1002626. ; PMCID: PMC4461318. 26057340 PMC4461318

[4] Goodman SN, Fanelli D, Ioannidis JP. What does research reproducibility mean? Sci Transl Med. 2016 Jun 1;8(341):341ps12. doi: 10.1126/scitranslmed.aaf5027 . 27252173

[5] Hutchinson L, Kirk R. High drug attrition rates—where are we going wrong? Nat Rev Clin Oncol. 2011 Mar 30;8(4):189–90. doi: 10.1038/nrclinonc.2011.34 . 21448176

[6] Voehringer P, Nicholson JR. Minimum Information in In Vivo Research. Handb Exp Pharmacol. 2020;257:197–222. doi: 10.1007/164_2019_285 . 31541320

[7] Colquhoun D. An investigation of the false discovery rate and the misinterpretation of p-values. R Soc Open Sci. 2014 Nov 19;1(3):140216. doi: 10.1098/rsos.140216 ; PMCID: PMC4448847. 26064558 PMC4448847

[8] Kafkafi N, Agassi J, Chesler EJ, Crabbe JC, Crusio WE, Eilam D, et al. Reproducibility and replicability of rodent phenotyping in preclinical studies. Neurosci Biobehav Rev. 2018 Apr;87:218–232. doi: 10.1016/j.neubiorev.2018.01.003 Epub 2018 Jan 31. ; PMCID: PMC6071910. 29357292 PMC6071910

[9] Kerr NL. HARKing: hypothesizing after the results are known. Pers Soc Psychol Rev. 1998;2(3):196–217. doi: 10.1207/s15327957pspr0203_4 . 15647155

[10] Prinz F, Schlange T, Asadullah K. Believe it or not: how much can we rely on published data on potential drug targets? Nat Rev Drug Discov. 2011 Aug 31;10(9):712. doi: 10.1038/nrd3439-c1 . 21892149

[11] Freedman LP, Gibson MC, Ethier SP, Soule HR, Neve RM, Reid YA. Reproducibility: changing the policies and culture of cell line authentication. Nat Methods. 2015 Jun;12(6):493–7. doi: 10.1038/nmeth.3403 . 26020501

[12] Steckler T. Editorial: preclinical data reproducibility for R&D—the challenge for neuroscience. Springerplus. 2015 Jan 13;4(1):1. doi: 10.1186/2193-1801-4-1 ; PMCID: PMC4320139. 25674489 PMC4320139

[13] Arroyo-Araujo M, Graf R, Maco M, van Dam E, Schenker E, Drinkenburg W, et al. Reproducibility via coordinated standardization: a multi-center study in a Shank2 genetic rat model for Autism Spectrum Disorders. Sci Rep. 2019 Aug 12;9(1):11602. doi: 10.1038/s41598-019-47981-0 ; PMCID: PMC6690904. 31406134 PMC6690904

[14] Arroyo-Araujo M, Voelkl B, Laloux C, Novak J, Koopmans B, Waldron AM, et al. Systematic assessment of the replicability and generalizability of preclinical findings: Impact of protocol harmonization across laboratory sites. PloS Biol. 2022 Nov 23;20(11):e3001886. doi: 10.1371/journal.pbio.3001886 ; PMCID: PMC9728859. 36417471 PMC9728859

[15] Bespalov A, Bernard R, Gilis A, Gerlach B, Guillén J, Castagné V, et al. Introduction to the EQIPD quality system. Elife. 2021 May 24;10:e63294. doi: 10.7554/eLife.63294 ; PMCID: PMC8184207. 34028353 PMC8184207

[16] Bissell M. Reproducibility: The risks of the replication drive. Nature. 2013 Nov 21;503(7476):333–4. doi: 10.1038/503333a . 24273798

[17] Bustin SA. The reproducibility of biomedical research: Sleepers awake! Biomol Detect Quantif. 2015 Jan 21;2:35–42. doi: 10.1016/j.bdq.2015.01.002 ; PMCID: PMC5121206. 27896142 PMC5121206

[18] Head ML, Holman L, Lanfear R, Kahn AT, Jennions MD. The extent and consequences of p-hacking in science. PloS Biol. 2015 Mar 13;13(3):e1002106. doi: 10.1371/journal.pbio.1002106 ; PMCID: PMC4359000. 25768323 PMC4359000

[19] Simonsohn U, Nelson LD, Simmons JP. P-Curve and Effect Size: Correcting for Publication Bias Using Only Significant Results. Perspect Psychol Sci. 2014 Nov;9(6):666–81. doi: 10.1177/1745691614553988 . 26186117

[20] Tsilidis KK, Panagiotou OA, Sena ES, Aretouli E, Evangelou E, Howells DW, et al. Evaluation of excess significance bias in animal studies of neurological diseases. PloS Biol. 2013 Jul;11(7):e1001609. doi: 10.1371/journal.pbio.1001609 Epub 2013 Jul 16. ; PMCID: PMC3712913. 23874156 PMC3712913

[21] Voelkl B, Vogt L, Sena ES, Würbel H. Reproducibility of preclinical animal research improves with heterogeneity of study samples. PloS Biol. 2018 Feb 22;16(2):e2003693. doi: 10.1371/journal.pbio.2003693 ; PMCID: PMC5823461. 29470495 PMC5823461

[22] Leiser SC, Dunlop J, Bowlby MR, Devilbiss DM. Aligning strategies for using EEG as a surrogate biomarker: a review of preclinical and clinical research. Biochem Pharmacol. 2011 Jun 15;81(12):1408–21. doi: 10.1016/j.bcp.2010.10.002 Epub 2010 Oct 19. . 20937262

[23] Wilson FJ, Leiser SC, Ivarsson M, Christensen SR, Bastlund JF. Can pharmaco-electroencephalography help improve survival of central nervous system drugs in early clinical development? Drug Discov Today. 2014 Mar;19(3):282–8. doi: 10.1016/j.drudis.2013.08.001 Epub 2013 Aug 14. . 23954252

[24] Drinkenburg WHIM, Ahnaou A, Ruigt GS. Pharmaco-EEG Studies in Animals: A History-Based Introduction to Contemporary Translational Applications. Neuropsychobiology. 2015;72(3–4):139–50. doi: 10.1159/000443175 Epub 2016 Feb 23. . 26901675

[25] Drinkenburg WHIM Ruigt GSF, Jobert M, editors. Essentials and Applications of EEG Research in Preclinical and Clinical Pharmacology. Unipublish Verlag für Studium & Praxis, Berlin, 2004; ISBN 3-938212-00-4 (9783938212004).

[26] Drinkenburg WHIM, Ruigt GS, Ahnaou A. Pharmaco-EEG Studies in Animals: An Overview of Contemporary Translational Applications. Neuropsychobiology. 2015;72(3–4):151–64. doi: 10.1159/000442210 Epub 2016 Feb 23. . 26901596

[27] Platt B, Welch A, Riedel G. FDG-PET imaging, EEG and sleep phenotypes as translational biomarkers for research in Alzheimer’s disease. Biochem Soc Trans. 2011 Aug;39(4):874–80. doi: 10.1042/BST0390874 . 21787316

[28] Cecchi M, Adachi M, Basile A, Buhl DL, Chadchankar H, Christensen S, et al. Validation of a suite of ERP and QEEG biomarkers in a pre-competitive, industry-led study in subjects with schizophrenia and healthy volunteers. Schizophr Res. 2023 Apr;254:178–189. doi: 10.1016/j.schres.2023.02.018 Epub 2023 Mar 13. . 36921403

[29] Schomer DL, Lopes da Silva FH, editors. Niedermeyer’s electroencephalography. Oxford Medicine Online. 2017; doi: 10.1093/med/9780190228484.001.0001

[30] Santana-Gomez C, Andrade P, Hudson MR, Paananen T, Ciszek R, Smith G, et al. Harmonization of pipeline for detection of HFOs in a rat model of post-traumatic epilepsy in preclinical multicenter study on post-traumatic epileptogenesis. Epilepsy Res. 2019 Oct;156:106110. doi: 10.1016/j.eplepsyres.2019.03.008 Epub 2019 Mar 15. PMCID: PMC6736751. 30981541 PMC6736751

[31] Ono T, Wagenaar J, Giorgi FS, Fabera P, Hanaya R, Jefferys J, et al. A companion to the preclinical common data elements and case report forms for rodent EEG studies. A report of the TASK3 EEG Working Group of the ILAE/AES Joint Translational Task Force. Epilepsia Open. 2018 Sep 24;3(Suppl Suppl 1):90–103. doi: 10.1002/epi4.12260 ; PMCID: PMC6210053. 30450486 PMC6210053

[32] Hernan AE, Schevon CA, Worrell GA, Galanopoulou AS, Kahane P, de Curtis M, et al. Methodological standards and functional correlates of depth in vivo electrophysiological recordings in control rodents. A TASK1-WG3 report of the AES/ILAE Translational Task Force of the ILAE. Epilepsia. 2017 Nov;58 Suppl 4(Suppl 4):28–39. doi: 10.1111/epi.13905 ; PMCID: PMC5679263. 29105069 PMC5679263

[33] Scott L, Kiss T, Kawabe TT, Hajós M. Neuronal network activity in the hippocampus of tau transgenic (Tg4510) mice. Neurobiol Aging. 2016 Jan;37:66–73. doi: 10.1016/j.neurobiolaging.2015.10.002 Epub 2015 Oct 14. . 26610388

[34] Booth CA, Witton J, Nowacki J, Tsaneva-Atanasova K, Jones MW, Randall AD, et al. Altered Intrinsic Pyramidal Neuron Properties and Pathway-Specific Synaptic Dysfunction Underlie Aberrant Hippocampal Network Function in a Mouse Model of Tauopathy. J Neurosci. 2016 Jan 13;36(2):350–63. doi: 10.1523/JNEUROSCI.2151-15.2016 ; PMCID: PMC4710765. 26758828 PMC4710765

[35] Holton CM, Hanley N, Shanks E, Oxley P, McCarthy A, Eastwood BJ, et al. Longitudinal changes in EEG power, sleep cycles and behaviour in a tau model of neurodegeneration. Alzheimers Res Ther. 2020 Jul 15;12(1):84. doi: 10.1186/s13195-020-00651-0 ; PMCID: PMC7364634. 32669112 PMC7364634

[36] Usui T, Macleod MR, McCann SK, Senior AM, Nakagawa S. Meta-analysis of variation suggests that embracing variability improves both replicability and generalizability in preclinical research. PloS Biol. 2021 May 19;19(5):e3001009. doi: 10.1371/journal.pbio.3001009 ; PMCID: PMC8168858. 34010281 PMC8168858

[37] Pradhan S, Gautam K, Pant V. Variation in Laboratory Reports: Causes other than Laboratory Error. JNMA J Nepal Med Assoc. 2022 Feb 15;60(246):222–224. doi: 10.31729/jnma.6022 ; PMCID: PMC9200014. 35210649 PMC9200014

[38] Jyoti A, Plano A, Riedel G, Platt B. EEG, activity, and sleep architecture in a transgenic AβPPswe/PSEN1A246E Alzheimer’s disease mouse. J Alzheimers Dis. 2010;22(3):873–87. doi: 10.3233/JAD-2010-100879 . 20858963

[39] Mehak SF, Shivakumar AB, Kumari S, Muralidharan B, Gangadharan G. Theta and gamma oscillatory dynamics in mouse models of Alzheimer’s disease: A path to prospective therapeutic intervention. Neurosci Biobehav Rev. 2022 May;136:104628. doi: 10.1016/j.neubiorev.2022.104628 Epub 2022 Mar 22. . 35331816

[40] Tok S, Maurin H, Delay C, Crauwels D, Manyakov NV, Van Der Elst W, et al. Neurophysiological effects of human-derived pathological tau conformers in the APPKM670/671NL.PS1/L166P amyloid mouse model of Alzheimer’s disease. Sci Rep. 2022 May 11;12(1):7784. doi: 10.1038/s41598-022-11582-1 ; PMCID: PMC9094605. 35546164 PMC9094605

[41] Hakami T, Jones NC, Tolmacheva EA, Gaudias J, Chaumont J, Salzberg M, et al. NMDA receptor hypofunction leads to generalized and persistent aberrant gamma oscillations independent of hyperlocomotion and the state of consciousness. PloS One. 2009 Aug 25;4(8):e6755. doi: 10.1371/journal.pone.0006755 ; PMCID: PMC2727800. 19707548 PMC2727800

[42] Gonzalez-Burgos I, Bainier M, Gross S, Schoenenberger P, Ochoa JA, Valencia M, et al. Glutamatergic and GABAergic Receptor Modulation Present Unique Electrophysiological Fingerprints in a Concentration-Dependent and Region-Specific Manner. eNeuro. 2023 Apr 20;10(4):ENEURO.0406-22.2023. doi: 10.1523/ENEURO.0406-22.2023 ; PMCID: PMC10124153. 36931729 PMC10124153

[43] Allouch S, Kabbara A, Duprez J, Khalil M, Modolo J, Hassan M. Effect of channel density, inverse solutions and connectivity measures on EEG resting-state networks reconstruction: A simulation study. Neuroimage. 2023 May 1;271:120006. doi: 10.1016/j.neuroimage.2023.120006 Epub 2023 Mar 11. . 36914106

[44] Lim CJM, Platt B, Janhunen SK, Riedel G. Comparison of automated video tracking systems in the open field test: ANY-Maze versus EthoVision XT. J Neurosci Methods. 2023 Sep 1;397:109940. doi: 10.1016/j.jneumeth.2023.109940 Epub 2023 Aug 6. . 37544382

[45] Kelly A, Ballerini L, Lowery M, Biggs M. 7.32 engineering the neural interface. Comprehensive Biomaterials II. 2017;642–60. doi: 10.1016/b978-0-12-803581-8.09322-x

[46] Kadam SD D’Ambrosio, Duveau, Roucard C, Garcia-Cairasco N, Ikeda A, et al. Methodological standards and interpretation of video-electroencephalography in adult control rodents. A TASK1-WG1 report of the AES/ILAE Translational Task Force of the ILAE. Epilepsia. 2017 Nov;58 Suppl 4(Suppl 4):10–27. doi: 10.1111/epi.13903 ; PMCID: PMC5679281. 29105073 PMC5679281

[47] King H, Reiber M, Philippi V, Stirling H, Aulehner K, Bankstahl M, et al. Anesthesia and analgesia for experimental craniotomy in mice and rats: a systematic scoping review comparing the years 2009 and 2019. Front Neurosci. 2023 May 3;17:1143109. doi: 10.3389/fnins.2023.1143109 ; PMCID: PMC10188949. 37207181 PMC10188949

[48] Robel S, Buckingham SC, Boni JL, Campbell SL, Danbolt NC, Riedemann T, et al. Reactive astrogliosis causes the development of spontaneous seizures. J Neurosci. 2015 Feb 25;35(8):3330–45. doi: 10.1523/JNEUROSCI.1574-14.2015 ; PMCID: PMC4339349. 25716834 PMC4339349

[49] Hauglund NL, Kusk P, Kornum BR, Nedergaard M. Meningeal Lymphangiogenesis and Enhanced Glymphatic Activity in Mice with Chronically Implanted EEG Electrodes. J Neurosci. 2020 Mar 11;40(11):2371–2380. doi: 10.1523/JNEUROSCI.2223-19.2020 Epub 2020 Feb 11. ; PMCID: PMC7083292. 32047056 PMC7083292

[50] Evers J, Sridhar K, Liegey J, Brady J, Jahns H, Lowery M. Stimulation-induced changes at the electrode-tissue interface and their influence on deep brain stimulation. J Neural Eng. 2022 Jul 4;19(4). doi: 10.1088/1741-2552/ac7ad6 . 35728575

[51] Herreras O. Local Field Potentials: Myths and Misunderstandings. Front Neural Circuits. 2016 Dec 15;10:101. doi: 10.3389/fncir.2016.00101 ; PMCID: PMC5156830. 28018180 PMC5156830

[52] Otte E, Vlachos A, Asplund M. Engineering strategies towards overcoming bleeding and glial scar formation around neural probes. Cell Tissue Res. 2022 Mar;387(3):461–477. doi: 10.1007/s00441-021-03567-9 Epub 2022 Jan 14. ; PMCID: PMC8975777. 35029757 PMC8975777

[53] Aulehner K, Bray J, Koska I, Pace C, Palme R, Kreuzer M, et al. The impact of tethered recording techniques on activity and sleep patterns in rats. Sci Rep. 2022 Feb 24;12(1):3179. doi: 10.1038/s41598-022-06307-3 ; PMCID: PMC8873297. 35210444 PMC8873297

[54] Chaddad A, Wu Y, Kateb R, Bouridane A. Electroencephalography Signal Processing: A Comprehensive Review and Analysis of Methods and Techniques. Sensors (Basel). 2023 Jul 16;23(14):6434. doi: 10.3390/s23146434 ; PMCID: PMC10385593. 37514728 PMC10385593

[55] Jwo D-J, Chang W-Y, Wu I-H. Windowing Techniques, the welch method for improvement of Power Spectrum Estimation. Computers, Materials & Continua. 2021; 67(3):3983–4003. doi: Doi:10.32604/cmc.2021.014752

[56] Gulín-González J, Qiang L, Yunwei C, Chiarenza GA, Li M, Valdés-Sosa P. Erol Başar and the scientific revolution in nonlinear brain dynamics: A selective review. Int J Psychophysiol. 2020 Dec;158:419–431. doi: 10.1016/j.ijpsycho.2020.08.011 Epub 2020 Oct 31. . 33137353

[57] Sanei S, Chambers JA. EEG Signal Processing. Germany: Wiley. 2013; ISBN:9781118691236.

[58] Harper A. Mouse models of neurological disorders—a comparison of heritable and acquired traits. Biochim Biophys Acta. 2010 Oct;1802(10):785–95. doi: 10.1016/j.bbadis.2010.05.009 Epub 2010 May 25. . 20510357

[59] Adlan LG, Csordás-Nagy M, Bodosi B, Kalmár G, Nyúl LG, Nagy A, et al. Sleep-Wake Rhythm and Oscillatory Pattern Analysis in a Multiple Hit Schizophrenia Rat Model (Wisket). Front Behav Neurosci. 2022 Jan 28;15:799271. doi: 10.3389/fnbeh.2021.799271 ; PMCID: PMC8831724. 35153694 PMC8831724

[60] Platt B, Riedel G. The cholinergic system, EEG and sleep. Behav Brain Res. 2011 Aug 10;221(2):499–504. doi: 10.1016/j.bbr.2011.01.017 Epub 2011 Jan 14. . 21238497

[61] Leiser SC, Iglesias-Bregna D, Westrich L, Pehrson AL, Sanchez C. Differentiated effects of the multimodal antidepressant vortioxetine on sleep architecture: Part 2, pharmacological interactions in rodents suggest a role of serotonin-3 receptor antagonism. J Psychopharmacol. 2015 Oct;29(10):1092–105. doi: 10.1177/0269881115592347 Epub 2015 Jul 14. ; PMCID: PMC4579402. 26174134 PMC4579402

[62] Jobert M, Wilson FJ, Ruigt GS, Brunovsky M, Prichep LS, Drinkenburg WH, et al. Guidelines for the recording and evaluation of pharmaco-EEG data in man: the International Pharmaco-EEG Society (IPEG). Neuropsychobiology. 2012;66(4):201–20. Epub 2012 Oct 12. doi: 10.1159/000343478 . 23075830

[63] Jobert M, Wilson FJ, Roth T, Ruigt GS, Anderer P, Drinkenburg WH, et al. Guidelines for the recording and evaluation of pharmaco-sleep studies in man: the International Pharmaco-EEG Society (IPEG). Neuropsychobiology. 2013;67(3):127–67. doi: 10.1159/000343449 Epub 2013 Mar 16. . 23548759

[64] Hosseini MP, Hosseini A, Ahi K. A Review on Machine Learning for EEG Signal Processing in Bioengineering. IEEE Rev Biomed Eng. 2021;14:204–218. doi: 10.1109/RBME.2020.2969915 Epub 2021 Jan 22. . 32011262

[65] Tveit J, Aurlien H, Plis S, Calhoun VD, Tatum WO, Schomer DL, et al. Automated Interpretation of Clinical Electroencephalograms Using Artificial Intelligence. JAMA Neurol. 2023 Aug 1;80(8):805–812. doi: 10.1001/jamaneurol.2023.1645 ; PMCID: PMC10282956. 37338864 PMC10282956

[66] O’Donnell P, Rosen L, Alexander R, Murthy V, Davies CH, Ratti E. Strategies to Address Challenges in Neuroscience Drug Discovery and Development. Int J Neuropsychopharmacol. 2019 Jul 1;22(7):445–448. doi: 10.1093/ijnp/pyz027 ; PMCID: PMC6600465. 31139821 PMC6600465

[67] Percie du Sert N, Hurst V, Ahluwalia A, Alam S, Avey MT, Baker M, et al. The ARRIVE guidelines 2.0: updated guidelines for reporting animal research. J Physiol. 2020 Sep;598(18):3793–3801. doi: 10.1113/JP280389 Epub 2020 Jul 14. ; PMCID: PMC7610696. 32666574 PMC7610696

[68] Jankowsky JL, Zheng H. Practical considerations for choosing a mouse model of Alzheimer’s disease. Mol Neurodegener. 2017 Dec 22;12(1):89. doi: 10.1186/s13024-017-0231-7 ; PMCID: PMC5741956. 29273078 PMC5741956

[69] Ramsden M, Kotilinek L, Forster C, Paulson J, McGowan E, SantaCruz K, et al. Age-dependent neurofibrillary tangle formation, neuron loss, and memory impairment in a mouse model of human tauopathy (P301L). J Neurosci. 2005 Nov 16;25(46):10637–47. doi: 10.1523/JNEUROSCI.3279-05.2005 ; PMCID: PMC6725849. 16291936 PMC6725849

[70] Santacruz K, Lewis J, Spires T, Paulson J, Kotilinek L, Ingelsson M, et al. Tau suppression in a neurodegenerative mouse model improves memory function. Science. 2005 Jul 15;309(5733):476–81. doi: 10.1126/science.1113694 ; PMCID: PMC1574647. 16020737 PMC1574647

[71] Spires TL, Orne JD, SantaCruz K, Pitstick R, Carlson GA, Ashe KH, et al. Region-specific dissociation of neuronal loss and neurofibrillary pathology in a mouse model of tauopathy. Am J Pathol. 2006 May;168(5):1598–607. doi: 10.2353/ajpath.2006.050840 ; PMCID: PMC1606598. 16651626 PMC1606598

[72] Vollert J, Macleod M, Dirnagl U, Kas MJ, Michel MC, Potschka H, et al. The EQIPD framework for rigor in the design, conduct, analysis and documentation of animal experiments. Nat Methods. 2022 Nov;19(11):1334–1337. doi: 10.1038/s41592-022-01615-y . 36064774

[73] Phillips KG, Cotel MC, McCarthy AP, Edgar DM, Tricklebank M, O’Neill MJ, et al. Differential effects of NMDA antagonists on high frequency and gamma EEG oscillations in a neurodevelopmental model of schizophrenia. Neuropharmacology. 2012 Mar;62(3):1359–70. doi: 10.1016/j.neuropharm.2011.04.006 Epub 2011 Apr 19. . 21521646

[74] Lee J, Hudson MR, O’Brien TJ, Nithianantharajah J, Jones NC. Local NMDA receptor hypofunction evokes generalized effects on gamma and high-frequency oscillations and behavior. Neuroscience. 2017 Sep 1;358:124–136. doi: 10.1016/j.neuroscience.2017.06.039 Epub 2017 Jul 1. . 28676240

[75] Cui K, Yu Z, Xu L, Jiang W, Wang L, Wang X, et al. Behavioral features and disorganization of oscillatory activity in C57BL/6J mice after acute low dose MK-801 administration. Front Neurosci. 2022 Sep 14;16:1001869. doi: 10.3389/fnins.2022.1001869 ; PMCID: PMC9515662. 36188453 PMC9515662

[76] Sil A, Souza Matos M, Delibegovic M, Platt B. How stra(i)nge are your controls? A comparative analysis of metabolic phenotypes in commonly used C57BL/6 substrains. PloS One. 2023 Aug 2;18(8):e0289472. doi: 10.1371/journal.pone.0289472 ; PMCID: PMC10395817. 37531359 PMC10395817

