# Supplementary material for: Evaluation of variation in preclinical electroencephalographic (EEG) spectral power across multiple laboratories and experiments: An EQIPD study
Source: PLoS One. 2024 Oct 29;19(10):e0309521. doi: 10.1371/journal.pone.0309521 (PMC11521305; doi:10.1371/journal.pone.0309521)
Supplement: S1 Table — The table provides information regarding animals and the facility, husbandry and housing, surgical procedures and care, EEG recording conditions and parameters, hardware and software, data analysis methods and experimental procedures. (PDF) [file pone.0309521.s001.pdf]

S1 Table: Localisation

EXPERIMENTAL FACTOR	RESEARCH PARTNERS					
	**Lab 1**	**Lab 2**	**Lab 3**	**Lab 4**	**Lab 5**	**Lab 6**
ANIMALS						
Species	Mouse	Mouse	Mouse	Mouse	Mouse	Participated only in Ring Testing Phase
Strain	rTg4510	rTg4510	rTg4510	rTg4510	rTg4510	
Sex	Female	Female	Female	Female	Female	
Age (at evaluation)	~22 weeks	20-21 weeks	~23 weeks	22-23 weeks	22 weeks	
Weight (at evaluation)	Average: 26.4 g; Range: 23.4 – 30.4 g	Average: 26.31 g,; Range: 20.9 – 41.5 g	Not available	21 – 34 g	Average: 27.7 g; Range: 21.1 – 32.2 g	
Source	Jackson Laboratories, Bar Harbor, USA	Jackson Laboratories, Bar Harbor, USA	Jackson Laboratories, Bar Harbor, USA	Jackson Laboratories, Bar Harbor, USA	In-house, breeding pair from Jackson Laboratories, Bar Harbor, USA	
SPF	Yes	Yes	Yes	Yes	No	
Paradigm naïve	Yes	Yes	Yes	Yes	Yes	
Drug naïve	Yes	Yes	Yes	Yes	Yes	
Previous procedures	None	None	None	None	None	
ANIMAL HUSBANDRY						
Single/social housing (number of animals/cage)	Group-housed (n=5/cage) before surgery, single after surgery	Group-housed (n=4 - 5/cage) before surgery, single after surgery	Group-housed (n=5 - 6/cage) before surgery, single after surgery	Group-housed (n=4/cage) before surgery, single after surgery	Group-housed (n=3/cage)	
Food	Standard Chow	Pellet (GLP Vitamin fortified, Provimi Kliba AG, Kaiseraugst, Switzerland)	Ssniff standard chow pelleted food	Standard laboratory chow SAFE	Altromin Chow	
Drink	Tap water	Tap water	Tap water	Tap water	Tap water	
Feeding schedule (restricted/ad libitum)	ad libitum	ad libitum	ad libitum	ad libitum	ad libitum	
Light-dark cycle (transition period)	12h:12h (7am:7pm)	12h:12h (6am:6pm), 30 min transitions	12h:12h (5:15am:5:15pm)	12h:12h (6am:6pm)	12h:12h (13:00-01:00)	
Light intensity	~500 lux	100-200 lux	<200 lux	n/a	50-100 lux	
Dark phase red light	No	No	No	No	Yes	
Temperature range	21 ± 2 °C	22 ± 2 °C	20 - 24 °C	20 - 23 °C	20 - 24 °C	
Humidity range	55 ± 5%	55 ± 10%	45 - 60%	30 - 70%	50 - 60%	

Ambient Noise level (quiet, radio 12/7, radio 24/7)	Radio 12/7 (quiet during recording)	Radio 12/7	Quiet	Quiet	Radio 24/7	
Home cage ventilation (IVC or conventional)	Conventional	IVC	Conventional	IVC	Conventional	
Cleaning frequency	Weekly	Weekly	Weekly	Weekly	Weekly	
Handling method	Tail handling	Tail handling	Tail handling	Scruff	Tail handling	
Handling frequency	Once per week	Once per Week; daily during experiment	Twice per week	Once per week	Once per week	
Environmental enrichment type	Tubes, chew sticks	Animal houses, nesting material, wood block	Animal houses, nesting material	Animal houses, nesting material, nylon bone	Animal houses, nesting material, paper roll	
SURGERY						
Survival rate of surgery	100 %	78 % (32/41)	100 %	100 %	100 %	
Anaesthesia used (inhalation dose)	Isoflurane (induced: 4%; maintained: 2%)	Isoflurane (1.5 - 4%)	Isoflurane (induced: 4%; maintained: 2%)	Isoflurane (induced: 4%; maintained: 2%)	Isoflurane (1 - 3%)	
Local anaesthesia used (dose)	Bupivacaine (0.1ml, 0.5% solution)	Lidocaine (0.1ml, 2% solution)	Bupivacaine (0.1ml, 0.5% solution)	None	Lidocaine (0.1ml, 20mg/ml)	
Local anaesthesia used (route of administration)	Topical	Topical	Subcutaneously	None	Subcutaneously	
Analgesia used (dose)	Carprofen (0.3ml) and buprenorphine (0.15ml), s.c.	Meloxicam (4 mg/kg), s.c.	Meloxicam (1 mg/kg), s.c.	Carprofen (5 mg/kg) and buprenorphine (0.05 mg/kg), s.c.	Flunixin meglumine (0.4ml/100g), s.c.	
Surgery duration per animal	~40 mins	2-3 hrs	~80 mins	30 mins	2 hrs	
Post-surgery recovery duration	2 weeks	1 - 2 weeks	2 weeks	2 weeks	2 weeks	
Surgical procedure used	Stereotaxic electrode implantation	Electrode placement by drilling in skull and s.c. placement of transmitter	Stereotaxic surface screw electrode implantation with s.c. placement of transmitter	Stereotaxic electrode implantation	Stereotaxic surgery with i.p. placement of transmitter	
Fluids given during surgery	Saline (1 ml)	No	No	Yes	Saline (0.1 ml/h)	
Body temp maintained during surgery	Yes	Yes	Yes	Yes	Yes	
EEG						
Number of recording electrodes	2	1	2	1	1	
Number of reference electrodes	2	1	2	1	1	
Screw size or array weight/size	Screw: 0.8mm diameter / 24.5mg	Screw: 2.16 mm head diameter, 1.19 mm shaft diameter, 1.60 mm shaft length	M 1.2 x 2 [mm]; 1.2 mm diameter / 30 mg	Screw: 0.25 cm length	DSI wire loop	

Cementing agent	CA glue, dental acrylic	PermaCem Automix	Dental acrylic (Paladur®)	Methyl methacrylate	UV activated dental cement (RelyX Unicem Apicap (shade: A1))	
Recording system (tethered/telemetry)	Wireless: stored on device	Telemetry: DSI (HD-X02)	Telemetry: DSI (HD-X02)	Tethered: Pinnacle Technology Inc. (8401-HS)	Telemetry: DSI (FT20)	
Recording software (version)	N/A (data recorded directly onto device)	Ponemah (DSI, v. 6.41)	Ponemah (DSI, v. 5.2.0)	Sirenia (Pinnacle Technology, v. 2)	Dataquest ART (DSI)	
Sampling Rate	200 Hz	500 Hz	1000 Hz (Batch1), 500 Hz (Batch2)	1 kHz	1000 Hz	
Sampling Filters	None	None	1.5 - 80 Hz	0.5 Hz - 1 kHz	None	
Sampling Gain	2 mV	Not specified by manufacturer	Not specified by manufacturer	10 x	Not specified by manufacturer	
STUDY						
Recording environment (home cage/cylinder)	Phenotyper homecages	Recording cage	New cage	Cylinder	Home cage	
Single/social housing (number of animals/cage) during recordings	Single	Single	Single	Single	Single	
Test duration	96 hrs	72 hrs	72 hrs	48 hrs	72 hrs	
Sample size rationale used	Power calculation	Based on historical data	Power calculation	Based on historical data	Based on historical data	
Randomisation to test group method	None	R-script by WP5	None	None	WP5 script by WP5	
Was experimenter blinded	No	No	No	No	Yes	
ANALYSIS						
Analysis software	Matlab	Neuroscore (DSI, v. 3.3.9317-1)	Neuroscore (DSI)	Neuroscore (DSI)	Neuroscore (DSI, v. 3.3.9317-1)	
Was a log transform used?	Yes	No	No	No	No	
Windowing method	Hanning	Hamming	Hamming	Hamming	Hamming	
Epoch size	4 sec	10 sec	5 min	10 sec	10 sec	
Filters used (incl. cut-off frequencies)	Bandpass (0.75 - 48 Hz)	None	None	Bandpass (0.5 Hz - 1 kHz)	Lowpass 250 Hz	

Describe briefly the performance of localised analysis in a few sentences (artifact removal, normalization, calculation of relative power etc.)	Data were bandpass filtered, epoched and artifacts rejected semi-automatically. Spectra calculated via autoregressive modelling. Absolute and relative band powers averaged over hourly bins. Epochs also sleep scored based off accelerometer movement (sleep/wake) and delta/theta ratio (NREM/REM).	Raw data was imported into NeuroScore and 72h of recording were selected per animal after visual inspection of the signal quality. Artifacts were removed manually based on amplitude +- exceeding 200 microvolts or flat/drifting signal and removed from power spectral analysis. The power spectra per 10s epoch were exported to Excel with total power and power values per frequency band. Relative power per 10 sec epoch was Normalized to total power and was aggregated in 1h bins.	For batch 1, data were reduced from 1000 Hz to 500 Hz. Absolute and relative band powers were averaged for each 1-hour time bin over the 72-hour period and compared between wildtype and transgenic mice. Artifact analysis was performed on signals with a 500 Hz sampling rate by applying an amplitude detector. All epochs with the signal´s amplitude over 1 mV were considered artifacts.	Manual artifact removal based on signal amplitude; Power spectral density plots consisting of standardized frequency band subdivisions were created for each 10 second bin and averaged for each 1 hour epoch for the recording and represented as a mean and SEM by group.	Manual artifact removal based on signal amplitude >200mV and signal drift or loss, data were filtered low-pass 250Hz and relative power was Normalized to total power. Power band values were exported directly from NeuroScore.	
Exclusion of animals: number and reason	n=5: EEG signal contaminated by artifacts	n= 5: low signal quality; n= 3 excessive artifacts; n= 1 low signal amplitude	n= 3: chewed tail; n= 4: excessive body weight loss; n= 1: poor wound healing	None	None	

S1 Table. Experimental parameters across laboratories during the Localisation Phase. The table provides information regarding animals and the facility, husbandry and housing, surgical procedures and care, EEG recording conditions and parameters, hardware and software, data analysis methods and experimental procedures.
