# Supplementary material for: Evaluation of variation in preclinical electroencephalographic (EEG) spectral power across multiple laboratories and experiments: An EQIPD study
Source: PLoS One. 2024 Oct 29;19(10):e0309521. doi: 10.1371/journal.pone.0309521 (PMC11521305; doi:10.1371/journal.pone.0309521)
Supplement: S3 Table — The table provides information regarding animals and the facility, husbandry and housing, surgical procedures and care, EEG recording conditions and parameters, hardware and software, data analysis methods and experimental procedures. (PDF) [file pone.0309521.s003.pdf]

S3 Table: Ring-Testing

EXPERIMENTAL FACTOR	RESEARCH PARTNERS					
	**Lab 1**	**Lab 2**	**Lab 3**	**Lab 4**	**Lab 5**	**Lab 6**
ANIMALS						
Species	Mouse	Mus musculus	Mouse	Mouse	Mouse	Mouse
Strain	C57BL/6J	C57BL/6JRj	C57BL/6J	C57BL/6J	C57BL/6J-CR	C57BL/6JRj
Sex	Female	Female	Female	Female	Female	Female
Age (at evaluation)	12-14 weeks	14 weeks	12-14 weeks	12-14 weeks	13-14 weeks	12-14 weeks
Weight (at evaluation)	Average: 24.0 g; Range: 22.1 - 25.7 g	Average: 23.3 g; Range: 22.2 - 24.95 g	Average: 24.9 g; Range: 24.0 - 25.9 g	Range: 18 - 23 g	Average: 21.75 g, Range: 19.3 - 25.6 g	Average: 22.54 g; Range: 20.6g - 25.96 g
Source	Charles River facility UK	Janvier Labs	Charles River Laboratories	Jackson Laboratories, Bar Harbor, USA	Charles River Laboratories	Janvier, France
SPF	Yes	Yes	Yes	Yes	Yes	Yes
Paradigm naive	Yes	Yes	Yes	Yes	Yes	Yes
Drug naive	Yes	Yes	Yes	Yes	Yes	Yes
Previous procedures	None	None	None	None	None	None
ANIMAL HUSBANDRY						
Single/social housing (number of animals/cage)	Group-housed (n=4/cage) before surgery, single after	Group-housed (n=4 - 5/cage) before surgery, single after	Group-housed (n=4/cage) before surgery, paired (n=2) after	Group-housed (n=4/cage) before surgery, single after	Group-housed (n=4/cage) before surgery, paired (n=2) after	Single housed
Food	Standard Chow	Pellet (GLP Vitamin fortified, Provimi Kliba AG, Kaiseraugst, Switzerland)	Ssniff standard chow	Standard laboratory chow SAFE	Altromin Chow	Standard laboratory chow SAFE
Drink	Tap water	Tap water	Tap water	Tap water	Tap water	Tap water
Feeding schedule (restricted/ad libitum)	ad libitum	ad libitum	ad libitum	ad libitum	ad libitum	ad libitum
Light-dark cycle	12h:12h (7am:7pm)	12h:12h (6am:6pm), 30 min transitions	12h:12h (5am:5pm)	12h:12h (6am:6pm)	12h:12h (09:00:21:00)	12h:12h (6am:6pm), gradual rise/fall
Light intensity (in lux)	~500 lux	100-200 lux	<200 lux	n/a	50 - 100 lux	1000 lux
Dark phase red light	No	No	No	No	Yes	Yes
Temperature range	21 ± 2 °C	22 ± 2 °C	20 - 24 °C	20 - 23 °C	20 - 24 °C	20 - 22 °C
Humidity range	55 ± 5%	55 ± 10%	45 - 60%	30 - 70%	50 - 60%	45 - 50%
Ambient Noise level (quiet, radio 12/7, radio 24/7)	Radio 12/7 (quiet during recording)	Radio 12/7	Quiet	Quiet	Radio 24/7	Radio 24/7

Home cage ventilation	Conventional	IVC	Conventional	IVC	Conventional	IVC
Cleaning frequency	Weekly	Weekly	Weekly	Weekly	Weekly	1/2 weeks
Handling method	Tail handling	Tail handling	Tail handling	Scruff or tail handling	Tail handling	Tail handling
Handling frequency	Once per week	Once per Week; daily during experiment	Twice per week	Once per week	Twice per week	Once per week
Environmental enrichment type	Tubes, chew sticks	Animal houses, nesting material, wood block	Animal houses, nesting material, chew sticks	Animal houses, nesting material, nylon bone	Animal houses, nesting material, paper roll	Animal houses, nesting material
SURGERY						
Survival rate of surgery	100 %	70.6 % (12/17)	100 %	100 %	100 %	100 %
Anaesthesia used (dose)	Isoflurane (induced: 4%; maintained: 2%)	Isoflurane (1.5 - 4%)	Isoflurane (induced: 4%; maintained: 1.5 - 2%)	Isoflurane (1.5 - 4%)	Isoflurane (induced: 5%; maintained: 1 - 2%)	Isoflurane (induced: 5%; maintained: 1 - 2%)
Local anaesthesia used (dose)	Bupivacaine (0.1ml, 0.5% solution)	Lidocaine (0.1ml, 2% solution)	Bupivacaine (0.1ml, 0.5% solution)	None	Lidocaine (0.1ml, 2% solution)	Xylocaine topical
Local anaesthesia used (route of administration)	Topical	Topical	Subcutaneously	None	Subcutaneously	Topical
Analgesia used (dose)	Carprofen (0.3ml) and buprenorphine (0.15ml), s.c.	Meloxicam (4 mg/kg), s.c.	Meloxicam (1 mg/kg), s.c.	Carprofen (5 mg/kg) and buprenorphine (0.05 mg/kg), s.c.	Carprofen (5 mg/kg), s.c.	Piritramide (0.25 mg/kg), s.c.
Surgery duration per animal	~40 mins	2-3 hrs	~80 mins	30 mins	1 - 2 hrs	~1 hr
Post-surgery recovery duration	2 weeks	10-21 days	2 weeks	2 weeks	2 weeks	≥10 days
Surgical procedure used	Stereotaxic electrode implantation	Electrode placement by drilling in skull and i.p. placement of transmitter	Stereotaxic surface screw electrode implantation with s.c. implantation	Stereotaxic electrode implantation	Stereotaxic electrode implantation	Stereotaxic electrode implantation
Fluids given during surgery	Saline (1 ml)	Saline (0.1 ml/h)	No	Yes	Yes	No
Body temp maintained during surgery	Yes	Yes	Yes	Yes	Yes	Yes
EEG						
Number of recording electrodes	3	1	2	1	2	2 epidural
Number of reference electrodes	2	1	2	1	1	1
Screw size or array weight/size	Screw: 0.8mm diameter / 24.5mg	Screw: 2.16 mm head diameter, 1.19 mm shaft diameter, 1.60 mm shaft length	M 1.2 x 2 [mm]; 1.2 mm diameter / 30 mg	Screw: 0.25 cm length	DSI wire loop	Screw: 1 mm diameter

Cementing agent	CA glue, dental acrylic	Permacem Automix	Dental acrylic (Paladur)	Methyl methacrylate	RelyXTM Unicem 2 Automix (3M ESPE, Germany)	RelyXTM Unicem 2 Automix (3M ESPE, Germany)
Recording system (tethered/telemetry, include vendor + device type)	Wireless: stored on device	Telemetry: DSI (HD-X02)	Telemetry: DSI (HD-X02)	Tethered: Pinnacle Technology Inc. (8401-HS)	Wireless: TaiNi (20/16/72/LOA, TainiTec LTD)	Tethered
Recording software	N/A (data recorded directly onto device)	Ponemah (DSI, v. 6.41)	Ponemah (DSI, v. 5.2.0)	Sirenia (Pinnacle Technology, v. 2)	TAINILIVE (TainiTec LTD)	Biosemi Active Two (Amsterdam, NL)
Sampling Rate	200 Hz	500 Hz	1 kHz	1 kHz	1 kHz	512 Hz
Sampling Filters	None	None	1.5 - 80 Hz	0.5 Hz - 1 kHz	None	None
Sampling Gain	2 mV	Not specified by manufacturer	Not specified by manufacturer	10 x	Not specified by manufacturer	Not specified by manufacturer
STUDY						
Recording environment (home cage/cylinder)	Phenotyper homecages	Recording cage	New cage	Cylinder	Home cage	Home cage
Single/social housing (number of animals/cage) during recordings	Single	Single	Single	Single	Social housing (n= 2)	Single
Test duration	5 hrs per recording day	5 hrs per recording day	5 hrs per recording day	5 hrs per recording day	5 hrs per recording day	5 hrs per recording day
Sample size rationale used	Power calculation	Based on historical data	Power calculation	Based on historical data	Based on historical data	Based on historical data
Randomisation to test group method	Random number generator	Yes	Randomly; R-script	Yes	Yes	Yes
Blinding	No	No	Yes	No	Yes	Yes
ANALYSIS						
Analysis software	Matlab	Neuroscore (DSI, v. 3.3.9317-1)	Neuroscore (DSI)	Matlab	Neuroscore (DSI, v. 3.3.9317-1)	Custom
Was a log transform used?	Yes	No	No	No	No	NO
Windowing method	Hanning	Hamming	Hamming	Welch periodogram, Hamming	Hamming	Hanning
Epoch size	10 sec	10 sec	10 sec	10 sec	10 sec	4 sec (local), 10 sec (central)
Filters used (specify which kind of filter and the order or dB)	Bandpass (0.75 - 48 Hz)	None	None	Bandpass (0.5 Hz - 1 kHz)	Lowpass 250 Hz	Bessel (3rd order), high pass 0.5 Hz

Describe briefly the performance of localised analysis in a few sentences (artifact removal, normalization, calculation of relative power etc.)	Data were bandpass filtered and epoched. Epochs containing artifacts were excluded semi automatically. Remaining epochs had power spectra derived via autoregressive methods. Total power was calculated for each epoch in the 0-45 Hz range. Average baseline band powers calculated for 30-60 minutes before dosing. Average band powers calculated for 30-60 minutes after dose. Spectra normalised to total power.	Raw data was imported into NeuroScore and 30 min. pre-dose and 30-60 min post-dose were selected per animal after visual inspection of the signal quality. The power spectra per 10s epoch were exported to Excel with total power and power values per frequency band. Relative gamma power (32-48Hz) per 10 sec epoch was normalized to total power and was aggregated in 30 min. bins. Artifacts were removed automatically based on an internal script.	Gamma power (32-48 Hz) for 30-60 minutes before and after the treatment, as well as the total power (0.5-48 Hz) for 30-60 minutes after the treatment were extracted with Neuroscore software. Artifact analysis was performed by applying an amplitude detector. All epochs with the signal´s amplitude over 1 mV were considered artifacts. For each animal, the sum of absolute gamma power and absolute total power per 30-minute time point was calculated. Relative gamma power was calculated by dividing absolute gamma power 30-60 minutes post dose by absolute total power. Two-tailed paired t-tests were used for comparison between the vehicle and drug treated groups.	Manual artifact removal based on signal amplitude; Power spectral density plots consisting of standardized frequency band subdivisions were created for each 10 second bin and averaged for each 1-hour epoch for the recording and represented as a mean and SEM by group.	Raw data was imported into NeuroScore and 30 min. pre-dose and 30-60 min post-dose were selected per animal after visual inspection of the signal quality. The power spectra per 10s epoch were exported to Excel with total power and power values per frequency band. Relative gamma power per 10 sec epoch was normalized to total power and was aggregated in 30 min. bins. Artifacts were removed manually (epochs with artifacts were excluded from analysis).	Raw EEG signals were filtered between 1 - 100Hz, Bessel 3rd order and high-pass filter 0.5 Hz were applied. Artifacts detection and removal was performed automatically. Data analysis was performed as required by the protocol: signals were subjected to FFT, power was calculated for 10 sec epochs. Relative gamma power (32-48Hz) per 10 sec epoch was normalized to total power and was aggregated in 30 min. bins.
Exclusion of animals: number and reason	None	n= 1: poor signal quality	n= 1: stress-related behavior	None	None	None
DRUG TESTING						
Compound	MK-801	MK-801	MK-801	MK-801	MK-801	MK-801
Dose (mg/kg), i.p.	0.2	0, 0.05, 0.2	0.2	0, 0.05, 0.2, 0.8	0, 0.05, 0.2	0, 0.05, 0.2, 0.8
Dosing regimen	ZT2 on day 2 & 4	Single dose i.p. 1h after the baseline recordings followed by 4h of the post-dosing recordings	ZT2 on day 2 & 5	Single dose i.p. 1h after the baseline recordings followed by 4h of the post-dosing recordings	Single dose i.p. 1h after the baseline recordings followed by 4h of the post-dosing recordings	Single dose i.p. 1h after the baseline recordings followed by 4h of the post-dosing recordings
Time of dosing (ZT time)	9am (ZT2)	ZT3 - 3.5	ZT4	ZT3	ZT3.5	ZT3.5
Vehicle	Saline	Saline	Saline	Saline	Saline	Saline
Vehicle volume (ml/kg)	5	10	10	5	10	10
Food restricted during experiment	No	No	No	No	No	Yes
Water restricted during experiment	No	No	No	No	No	No

S3 Table. Experimental parameters across laboratories during the Ring Testing Phase. The table provides information regarding animals and the facility, husbandry and housing, surgical procedures and care, EEG recording conditions and parameters, hardware and software, data analysis methods and experimental procedures.
