# Supplementary material for: Evaluation of variation in preclinical electroencephalographic (EEG) spectral power across multiple laboratories and experiments: An EQIPD study
Source: PLoS One. 2024 Oct 29;19(10):e0309521. doi: 10.1371/journal.pone.0309521 (PMC11521305; doi:10.1371/journal.pone.0309521)
Supplement: S5 Table — The table shows estimated means, standard error, lower confidence limit (CL), and upper confidence limit (CL) of WT and TG groups, as well as their contrasts (TG-WT). The p-value was derived from the statistical models run per laboratory on log10 relative theta power data. Note that p-values are not provided for individual means as this was not of interest in this study. (PDF) [file pone.0309521.s005.pdf]

S5 Table

Contributor ID	Test group ID	mean	SE	lower CL	upper CL	p value
Lab 1	TG	-0.54	0.0074	-0.56	-0.53	-
Lab 1	WT	-0.53	0.0084	-0.55	-0.52	-
Lab 1	TG - WT	-0.01	0.0112	-0.03	0.01	0.4142
Lab 2	TG	-0.59	0.0105	-0.61	-0.57	-
Lab 2	WT	-0.67	0.0101	-0.69	-0.65	-
Lab 2	TG - WT	0.08	0.0146	0.05	0.11	p < 0.001
Lab 3	TG	-0.68	0.0149	-0.71	-0.65	-
Lab 3	WT	-0.7	0.0137	-0.72	-0.67	-
Lab 3	TG - WT	0.02	0.0202	-0.02	0.06	0.3747
Lab 4	TG	-0.58	0.006	-0.59	-0.57	-
Lab 4	WT	-0.58	0.0056	-0.59	-0.57	-
Lab 4	TG - WT	0	0.0082	-0.02	0.02	0.938
Lab 5	TG	-0.66	0.0291	-0.72	-0.6	-
Lab 5	WT	-0.7	0.0291	-0.76	-0.64	-
Lab 5	TG - WT	0.04	0.0411	-0.05	0.12	0.3995

S5 Table. Localisation phase relative theta power analysed locally by the partners. The table shows estimated means, standard error, lower confidence limit (CL), and upper confidence limit (CL) of WT and TG groups, as well as their contrasts (TG-WT). The p-value was derived from the statistical models run per laboratory on log10 relative theta power data. Note that p-values are not provided for individual means as this was not of interest in this study.
