# Supplementary material for: Evaluation of variation in preclinical electroencephalographic (EEG) spectral power across multiple laboratories and experiments: An EQIPD study
Source: PLoS One. 2024 Oct 29;19(10):e0309521. doi: 10.1371/journal.pone.0309521 (PMC11521305; doi:10.1371/journal.pone.0309521)
Supplement: S6 Table — The table shows estimated means, standard error, lower confidence limit (CL), and upper confidence limit (CL) of WT and TG groups, as well as their contrasts (TG-WT). The p-value was derived from the statistical models run per laboratory on log10 total power data. Note that p-values are not provided for individual means as this was not of interest in this study. (PDF) [file pone.0309521.s006.pdf]

S6 Table

Contributor ID	Test group ID	mean	SE	lower CL	upper CL	p value
Lab 1	TG	-1.04	0.1022	-1.27	-0.81	-
Lab 1	WT	-1.05	0.1209	-1.32	-0.78	-
Lab 1	TG – WT	0.01	0.1583	-0.35	0.36	0.9648
Lab 2	TG	-0.7	0.0907	-0.89	-0.51	-
Lab 2	WT	-0.28	0.0907	-0.47	-0.09	-
Lab 2	TG – WT	-0.42	0.1283	-0.69	-0.15	0.005
Lab 3	TG	-0.55	0.0506	-0.65	-0.44	-
Lab 3	WT	-0.31	0.0506	-0.42	-0.21	-
Lab 3	TG – WT	-0.23	0.0716	-0.38	-0.09	0.0035
Lab 4	TG	-0.8	0.128	-1.07	-0.54	-
Lab 4	WT	-0.57	0.128	-0.84	-0.31	-
Lab 4	TG – WT	-0.23	0.181	-0.6	0.15	0.2229
Lab 5	TG	-0.87	0.1637	-1.24	-0.5	-
Lab 5	WT	-0.96	0.1494	-1.3	-0.63	-
Lab 5	TG – WT	0.1	0.2217	-0.4	0.6	0.6677

S6 Table. **Harmonisation phase total power analysed locally by the partners.** The table shows estimated means, standard error, lower confidence limit (CL), and upper confidence limit (CL) of WT and TG groups, as well as their contrasts (TG-WT). The p-value was derived from the statistical models run per laboratory on log_{10} total power data. Note that p-values are not provided for individual means as this was not of interest in this study.
