# Supplementary material for: Evaluation of variation in preclinical electroencephalographic (EEG) spectral power across multiple laboratories and experiments: An EQIPD study
Source: PLoS One. 2024 Oct 29;19(10):e0309521. doi: 10.1371/journal.pone.0309521 (PMC11521305; doi:10.1371/journal.pone.0309521)
Supplement: S7 Table — The table shows estimated means of WT and TG groups as well as their contrast, with each the standard error, lower confidence limit (CL), upper confidence limit (CL), and p-value derived from the statistical models run per laboratory on log10 relative theta power data. Note that p-values are not provided for individual means as not of interest in this study. (PDF) [file pone.0309521.s007.pdf]

S7 Table

Contributor ID	Test group ID	mean	SE	lower CL	upper CL	p value
Lab 1	TG	-0.62	0.0202	-0.67	-0.58	-
Lab 1	WT	-0.59	0.0239	-0.64	-0.53	-
Lab 1	TG – WT	-0.04	0.0313	-0.11	0.03	0.2352
Lab 2	TG	-0.66	0.0166	-0.69	-0.62	-
Lab 2	WT	-0.66	0.0166	-0.7	-0.63	-
Lab 2	TG – WT	0	0.0235	-0.05	0.05	0.8905
Lab 3	TG	-0.68	0.0238	-0.73	-0.63	-
Lab 3	WT	-0.65	0.0238	-0.7	-0.6	-
Lab 3	TG – WT	-0.03	0.0336	-0.1	0.04	0.39
Lab 4	TG	-0.6	0.0213	-0.65	-0.56	-
Lab 4	WT	-0.55	0.0213	-0.6	-0.51	-
Lab 4	TG – WT	-0.05	0.0302	-0.11	0.02	0.1339
Lab 5	TG	-0.78	0.0544	-0.9	-0.66	-
Lab 5	WT	-0.83	0.0497	-0.94	-0.72	-
Lab 5	TG – WT	0.05	0.0737	-0.12	0.22	0.5128

S7 Table. Harmonisation phase relative theta power analysed centrally. The table shows estimated means of WT and TG groups as well as their contrast, with each the standard error, lower confidence limit (CL), upper confidence limit (CL), and p-value derived from the statistical models run per laboratory on $\log_{10}$ relative theta power data. Note that p-values are not provided for individual means as not of interest in this study.
