# Supplementary material for: Evaluation of variation in preclinical electroencephalographic (EEG) spectral power across multiple laboratories and experiments: An EQIPD study
Source: PLoS One. 2024 Oct 29;19(10):e0309521. doi: 10.1371/journal.pone.0309521 (PMC11521305; doi:10.1371/journal.pone.0309521)
Supplement: S8 Table — The table shows estimated means, standard error, lower confidence limit (CL), and upper confidence limit (CL) of pharmacological interventions and their contrasts. The p-value was derived from the statistical models run per laboratory on log10 relative gamma power data. Note that p-values are not provided for individual means as this was not of interest in this study. (PDF) [file pone.0309521.s008.pdf]

S8 Table

Contributor ID	Test group ID	mean	SE	lower CL	upper CL	p value
Lab 1	MK-801 0.2 mg/kg	-0.95	0.031	-1.02	-0.89	-
Lab 1	Vehicle	-1.36	0.031	-1.42	-1.29	-
**Lab 1**	**Vehicle – (MK-801 0.2 mg/kg)**	**-0.41**	**0.044**	**-0.5**	**-0.32**	**p < 0.001**
Lab 2	(MK-801 0.05 mg/kg) – (MK-801 0.2 mg/kg)	-0.05	0.074	-0.2	0.1	0.5293
Lab 2	MK-801 0.05 mg/kg	0.62	0.052	0.51	0.73	-
Lab 2	MK-801 0.2 mg/kg	0.67	0.052	0.56	0.77	-
Lab 2	Vehicle	0.62	0.052	0.51	0.73	-
Lab 2	Vehicle – (MK-801 0.05 mg/kg)	0	0.074	-0.15	0.15	0.9934
**Lab 2**	**Vehicle – (MK-801 0.2 mg/kg)**	**-0.05**	**0.074**	**-0.2**	**0.1**	**0.5239**
Lab 3	MK-801 0.2 mg/kg	-1.27	0.062	-1.4	-1.14	-
Lab 3	Vehicle	-1.59	0.062	-1.72	-1.47	-
**Lab 3**	**Vehicle – (MK-801 0.2 mg/kg)**	**-0.33**	**0.088**	**-0.51**	**-0.14**	**0.0011**
Lab 4	(MK-801 0.05 mg/kg) – (MK-801 0.2 mg/kg)	-0.02	0.054	-0.13	0.09	0.6856
Lab 4	MK-801 0.05 mg/kg	-2.79	0.039	-2.87	-2.71	-
Lab 4	MK-801 0.2 mg/kg	-2.77	0.038	-2.84	-2.69	-
Lab 4	Vehicle	-2.83	0.038	-2.91	-2.75	-
Lab 4	Vehicle – (MK-801 0.05 mg/kg)	-0.04	0.054	-0.15	0.07	0.4605
**Lab 4**	**Vehicle – (MK-801 0.2 mg/kg)**	**-0.06**	**0.053**	**-0.17**	**0.05**	**0.2455**
Lab 5	(MK-801 0.05 mg/kg) – (MK-801 0.2 mg/kg)	-0.25	0.046	-0.35	-0.16	p < 0.001
Lab 5	MK-801 0.05 mg/kg	-1.72	0.033	-1.79	-1.66	-
Lab 5	MK-801 0.2 mg/kg	-1.47	0.033	-1.54	-1.4	-
Lab 5	Vehicle	-1.66	0.033	-1.73	-1.6	-
Lab 5	Vehicle – (MK-801 0.05 mg/kg)	0.06	0.046	-0.03	0.15	0.2077
**Lab 5**	**Vehicle – (MK-801 0.2 mg/kg)**	**-0.19**	**0.046**	**-0.29**	**-0.1**	**p < 0.001**
Lab 6	(MK-801 0.05 mg/kg) – (MK-801 0.2 mg/kg)	-0.17	0.06	-0.3	-0.05	0.0061
Lab 6	MK-801 0.05 mg/kg	-4.12	0.042	-4.21	-4.04	-
Lab 6	MK-801 0.2 mg/kg	-3.95	0.042	-4.03	-3.86	-

Lab 6	Vehicle	-4.17	0.042	-4.26	-4.08	-
Lab 6	Vehicle – (MK-801 0.05 mg/kg)	-0.05	0.06	-0.17	0.07	0.4258
**Lab 6**	**Vehicle – (MK-801 0.2 mg/kg)**	**-0.22**	**0.06**	**-0.34**	**-0.1**	**p < 0.001**

S8 Table. Ring-testing phase relative gamma power analysed locally by the partners. The table shows estimated means, standard error, lower confidence limit (CL), and upper confidence limit (CL) of pharmacological interventions and their contrasts. The p-value was derived from the statistical models run per laboratory on log_{10} relative gamma power data. Note that p-values are not provided for individual means as this was not of interest in this study.
