# Supplementary material for: Evaluation of variation in preclinical electroencephalographic (EEG) spectral power across multiple laboratories and experiments: An EQIPD study
Source: PLoS One. 2024 Oct 29;19(10):e0309521. doi: 10.1371/journal.pone.0309521 (PMC11521305; doi:10.1371/journal.pone.0309521)
Supplement: S10 Table — The table shows estimated means, standard error, lower confidence limit (CL), and upper confidence limit (CL) of pharmacological interventions and their contrasts. The p-value was derived from the statistical models run per laboratory on log10 relative gamma power data. Note that p-values are not provided for individual means as this was not of interest in this study. (PDF) [file pone.0309521.s010.pdf]

S10 Table

Contributor ID	Test group ID	mean	SE	lower CL	upper CL	p value
Lab 1	MK-801 0.2 mg/kg	-1.09	0.049	-1.19	-0.99	-
Lab 1	Vehicle	-1.26	0.049	-1.36	-1.16	-
**Lab 1**	**Vehicle - (MK-801 0.2 mg/kg)**	**-0.17**	**0.0693**	**-0.31**	**-0.03**	**0.0229**
Lab 2	(MK-801 0.05 mg/kg) - (MK-801 0.2 mg/kg)	-0.21	0.0456	-0.3	-0.12	$p < 0.001$
Lab 2	MK-801 0.05 mg/kg	-1.57	0.0323	-1.64	-1.51	-
Lab 2	MK-801 0.2 mg/kg	-1.36	0.0323	-1.43	-1.3	-
Lab 2	Vehicle	-1.62	0.0323	-1.69	-1.56	-
Lab 2	Vehicle - (MK-801 0.05 mg/kg)	-0.05	0.0456	-0.14	0.04	0.2802
**Lab 2**	**Vehicle - (MK-801 0.2 mg/kg)**	**-0.26**	**0.0456**	**-0.35**	**-0.17**	**$p < 0.001$**
Lab 3	MK-801 0.2 mg/kg	-1.23	0.0264	-1.29	-1.18	-
Lab 3	Vehicle	-1.26	0.0264	-1.32	-1.21	-
**Lab 3**	**Vehicle - (MK-801 0.2 mg/kg)**	**-0.03**	**0.0374**	**-0.11**	**0.05**	**0.4474**
Lab 4	(MK-801 0.05 mg/kg) - (MK-801 0.2 mg/kg)	-0.03	0.0933	-0.22	0.16	0.7721
Lab 4	MK-801 0.05 mg/kg	-1.31	0.0675	-1.45	-1.17	-
Lab 4	MK-801 0.2 mg/kg	-1.28	0.0644	-1.41	-1.15	-
Lab 4	Vehicle	-1.29	0.0675	-1.42	-1.15	-
Lab 4	Vehicle - (MK-801 0.05 mg/kg)	0.02	0.0955	-0.17	0.22	0.8215
**Lab 4**	**Vehicle - (MK-801 0.2 mg/kg)**	**-0.01**	**0.0933**	**-0.2**	**0.19**	**0.9531**
Lab 5	(MK-801 0.05 mg/kg) - (MK-801 0.2 mg/kg)	-0.24	0.0653	-0.37	-0.1	0.001
Lab 5	MK-801 0.05 mg/kg	-1.82	0.0462	-1.92	-1.73	-
Lab 5	MK-801 0.2 mg/kg	-1.59	0.0462	-1.68	-1.49	-
Lab 5	Vehicle	-1.76	0.0462	-1.85	-1.66	-
Lab 5	Vehicle - (MK-801 0.05 mg/kg)	0.07	0.0653	-0.06	0.2	0.2958
**Lab 5**	**Vehicle - (MK-801 0.2 mg/kg)**	**-0.17**	**0.0653**	**-0.3**	**-0.03**	**0.0154**
Lab 6	(MK-801 0.05 mg/kg) - (MK-801 0.2 mg/kg)	-0.46	0.089	-0.64	-0.28	$p < 0.001$
Lab 6	MK-801 0.05 mg/kg	-1.39	0.063	-1.51	-1.26	-
Lab 6	MK-801 0.2 mg/kg	-0.93	0.063	-1.05	-0.8	-
Lab 6	Vehicle	-1.35	0.063	-1.48	-1.23	-
Lab 6	Vehicle - (MK-801 0.05 mg/kg)	0.03	0.089	-0.15	0.21	0.7267
**Lab 6**	**Vehicle - (MK-801 0.2 mg/kg)**	**-0.43**	**0.089**	**-0.61**	**-0.25**	**$p < 0.001$**

S10 Table. Ring-testing phase relative gamma power analysed centrally. The table shows estimated means, standard error, lower confidence limit (CL), and upper confidence limit (CL) of pharmacological interventions and their contrasts. The p-value was derived from the statistical models run per laboratory on $\log_{10}$ relative gamma power data. Note that p-values are not provided for individual means as this was not of interest in this study.
