# Supplementary material for: Evaluation of variation in preclinical electroencephalographic (EEG) spectral power across multiple laboratories and experiments: An EQIPD study
Source: PLoS One. 2024 Oct 29;19(10):e0309521. doi: 10.1371/journal.pone.0309521 (PMC11521305; doi:10.1371/journal.pone.0309521)
Supplement: S11 Table — The table shows estimated means, standard error, lower confidence limit (CL), and upper confidence limit (CL) of pharmacological interventions and their contrasts. The p-value was derived from the statistical models run per laboratory on raw gamma power percent change data. Note that p-values are not provided for individual means as this was not of interest in this study. (PDF) [file pone.0309521.s011.pdf]

S11 Table

Contributor ID	Test group ID	mean	SE	lower CL	upper CL	p value
Lab 1	MK-801 0.2 mg/kg	0.27	0.0926	0.08	0.46	-
Lab 1	Vehicle	0.16	0.0926	-0.03	0.35	-
**Lab 1**	**Vehicle - (MK-801 0.2 mg/kg)**	**-0.11**	**0.131**	**-0.38**	**0.16**	**0.398**
Lab 2	(MK-801 0.05 mg/kg) - (MK-801 0.2 mg/kg)	-0.12	0.0595	-0.25	0	0.0461
Lab 2	MK-801 0.05 mg/kg	0.04	0.0421	-0.04	0.13	-
Lab 2	MK-801 0.2 mg/kg	0.17	0.0421	0.08	0.25	-
Lab 2	Vehicle	0.05	0.0421	-0.04	0.13	-
Lab 2	Vehicle - (MK-801 0.05 mg/kg)	0	0.0595	-0.12	0.12	0.9624
**Lab 2**	**Vehicle - (MK-801 0.2 mg/kg)**	**-0.12**	**0.0595**	**-0.24**	**0**	**0.051**
Lab 3	MK-801 0.2 mg/kg	-0.03	0.0264	-0.08	0.03	-
Lab 3	Vehicle	-0.04	0.0274	-0.1	0.01	-
**Lab 3**	**Vehicle - (MK-801 0.2 mg/kg)**	**-0.02**	**0.0381**	**-0.1**	**0.06**	**0.6481**
Lab 4	(MK-801 0.05 mg/kg) - (MK-801 0.2 mg/kg)	0.02	0.0641	-0.11	0.15	0.7867
Lab 4	MK-801 0.05 mg/kg	0.05	0.0464	-0.04	0.15	-
Lab 4	MK-801 0.2 mg/kg	0.03	0.0442	-0.06	0.13	-
Lab 4	Vehicle	-0.01	0.0464	-0.1	0.09	-
Lab 4	Vehicle - (MK-801 0.05 mg/kg)	-0.06	0.0656	-0.19	0.07	0.3728
**Lab 4**	**Vehicle - (MK-801 0.2 mg/kg)**	**-0.04**	**0.0641**	**-0.17**	**0.09**	**0.5186**
Lab 5	(MK-801 0.05 mg/kg) - (MK-801 0.2 mg/kg)	0.08	0.1092	-0.14	0.3	0.4627
Lab 5	MK-801 0.05 mg/kg	0.09	0.0772	-0.07	0.24	-
Lab 5	MK-801 0.2 mg/kg	0	0.0772	-0.15	0.16	-
Lab 5	Vehicle	0.05	0.0772	-0.1	0.21	-
Lab 5	Vehicle - (MK-801 0.05 mg/kg)	-0.03	0.1092	-0.25	0.19	0.7684
**Lab 5**	**Vehicle - (MK-801 0.2 mg/kg)**	**0.05**	**0.1092**	**-0.17**	**0.27**	**0.6584**
Lab 6	(MK-801 0.05 mg/kg) - (MK-801 0.2 mg/kg)	-0.55	0.0691	-0.69	-0.41	p < 0.001
Lab 6	MK-801 0.05 mg/kg	0.03	0.0489	-0.07	0.13	-
Lab 6	MK-801 0.2 mg/kg	0.58	0.0489	0.48	0.68	-
Lab 6	Vehicle	-0.08	0.0489	-0.18	0.02	-
Lab 6	Vehicle - (MK-801 0.05 mg/kg)	-0.11	0.0691	-0.25	0.03	0.1241
**Lab 6**	**Vehicle - (MK-801 0.2 mg/kg)**	**-0.66**	**0.0691**	**-0.8**	**-0.52**	**p < 0.001**

S11 Table. Ring-testing phase gamma power as percent change from baseline using raw power analysed centrally. The table shows estimated means, standard error, lower confidence limit (CL), and upper confidence limit (CL) of pharmacological interventions and their contrasts. The p-value was derived from the statistical models run per laboratory on raw gamma power percent change data. Note that p-values are not provided for individual means as this was not of interest in this study.
