# Supplementary material for: Evaluation of variation in preclinical electroencephalographic (EEG) spectral power across multiple laboratories and experiments: An EQIPD study
Source: PLoS One. 2024 Oct 29;19(10):e0309521. doi: 10.1371/journal.pone.0309521 (PMC11521305; doi:10.1371/journal.pone.0309521)

Absolute Delta (1-3.9 Hz) Power

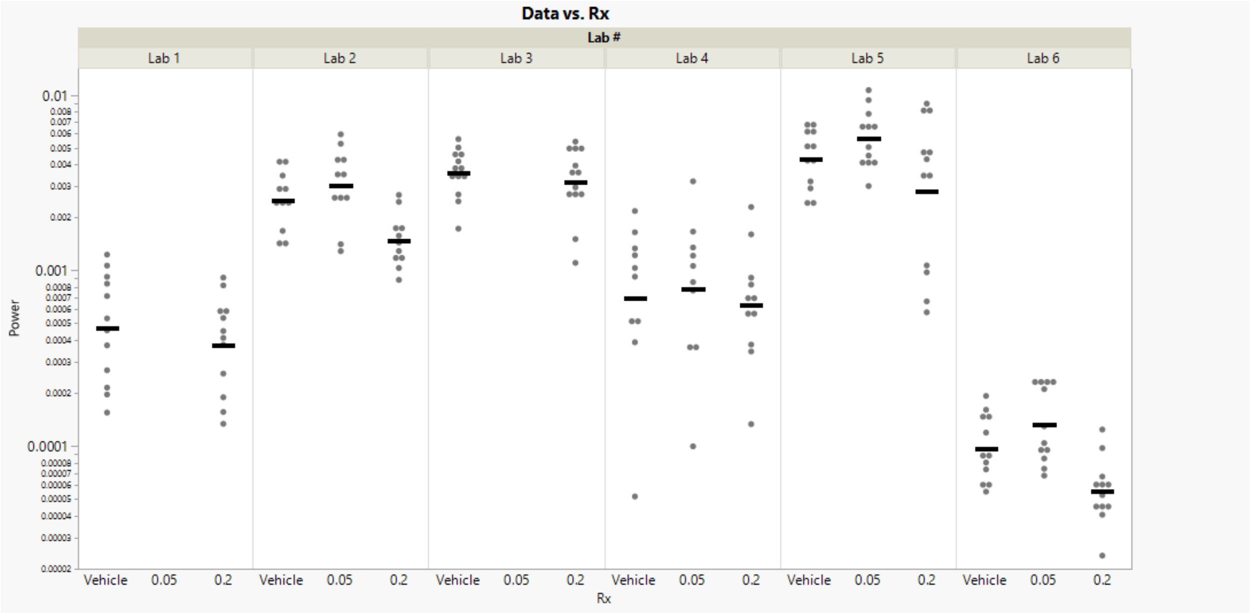

Absolute Theta-1 (4-5.9 Hz) Power

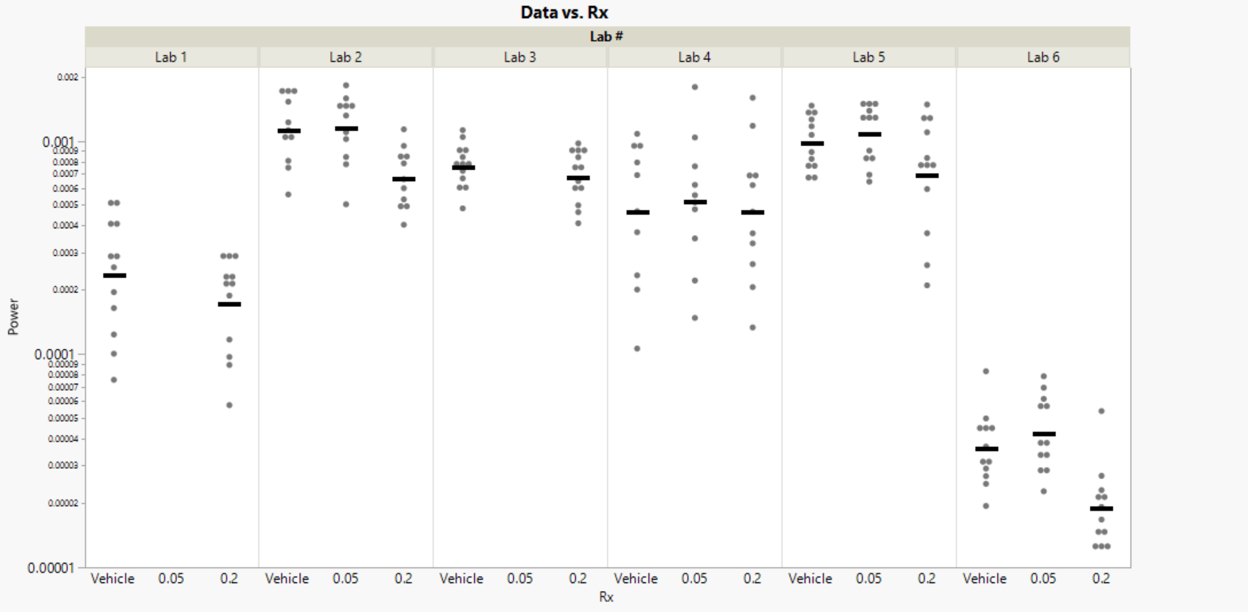

Absolute Theta-2 (6-7.9 Hz) Power

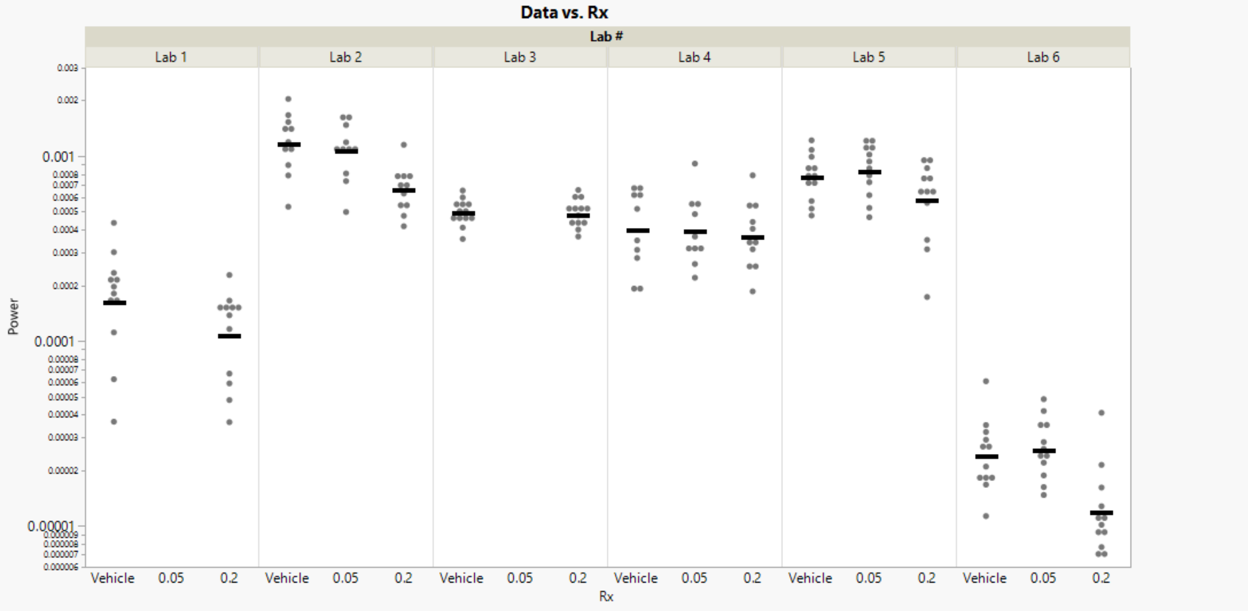

Absolute Alpha-1 (8-10.9 Hz) Power

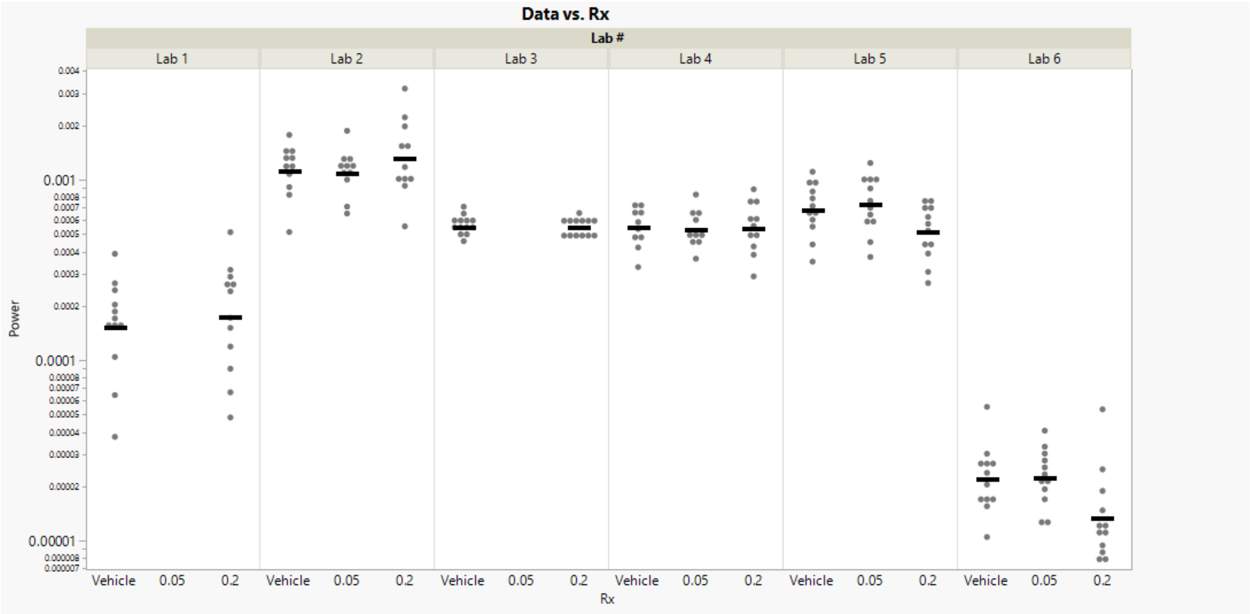

Absolute Alpha-2 (11-13.9 Hz) Power

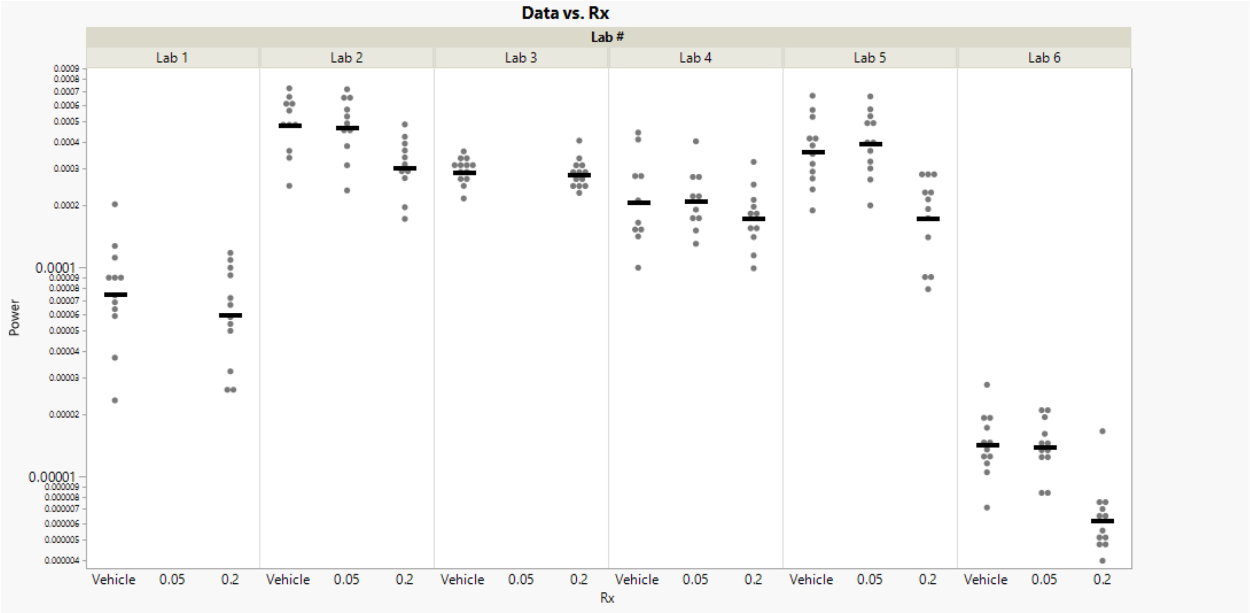

Absolute Beta-1 (14-17.9 Hz) Power

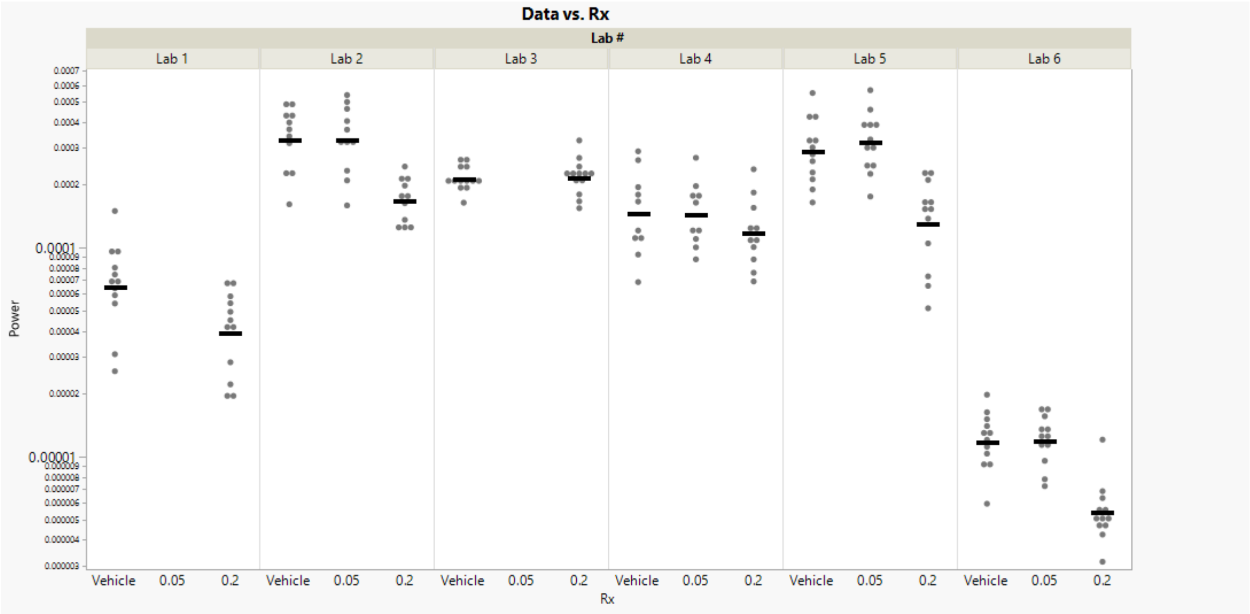

Absolute Beta-2 (18-31.9 Hz) Power

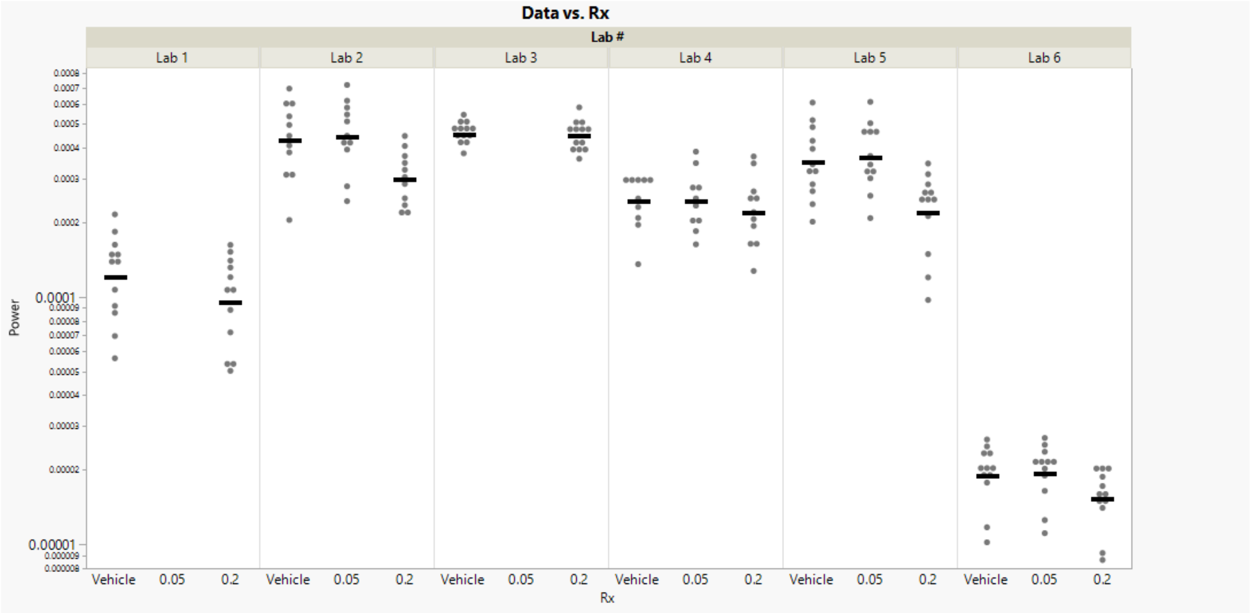

Absolute Gamma-1 (32-48 Hz) Power

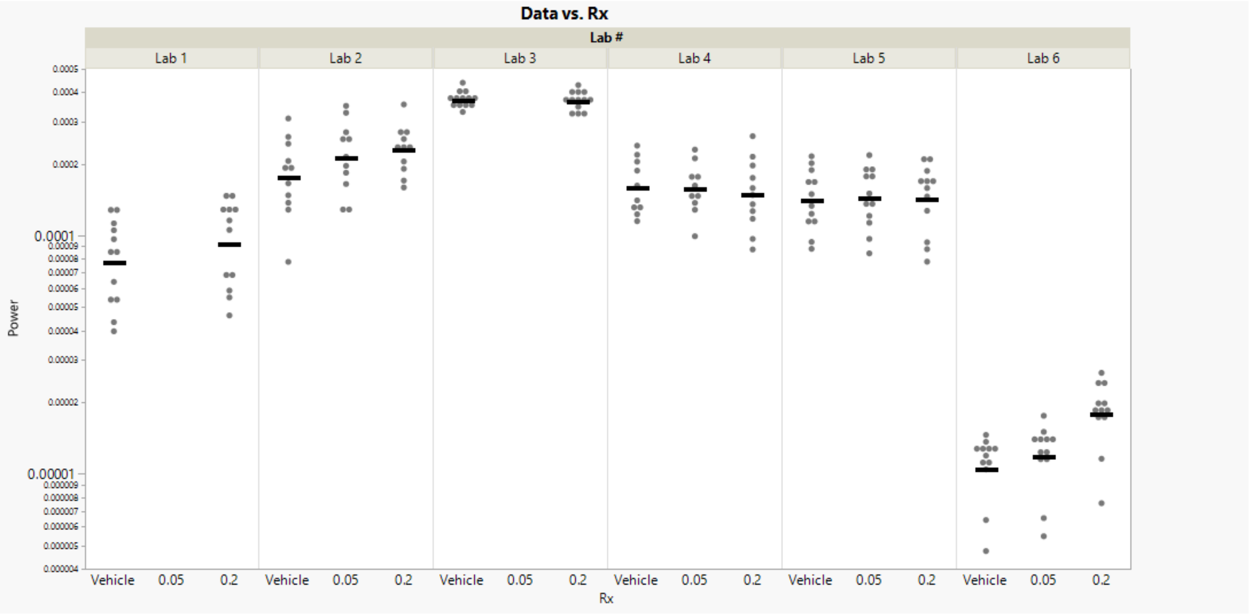

Relative Delta (1-3.9 Hz) Power

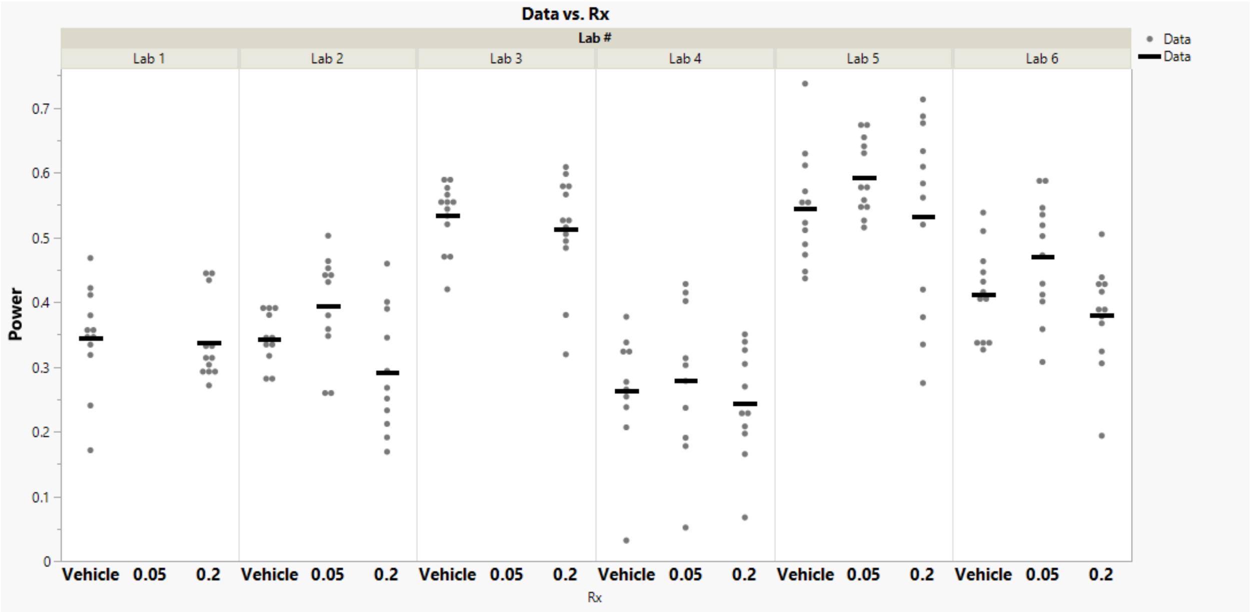

Relative Theta-1 (4-5.9 Hz) Power

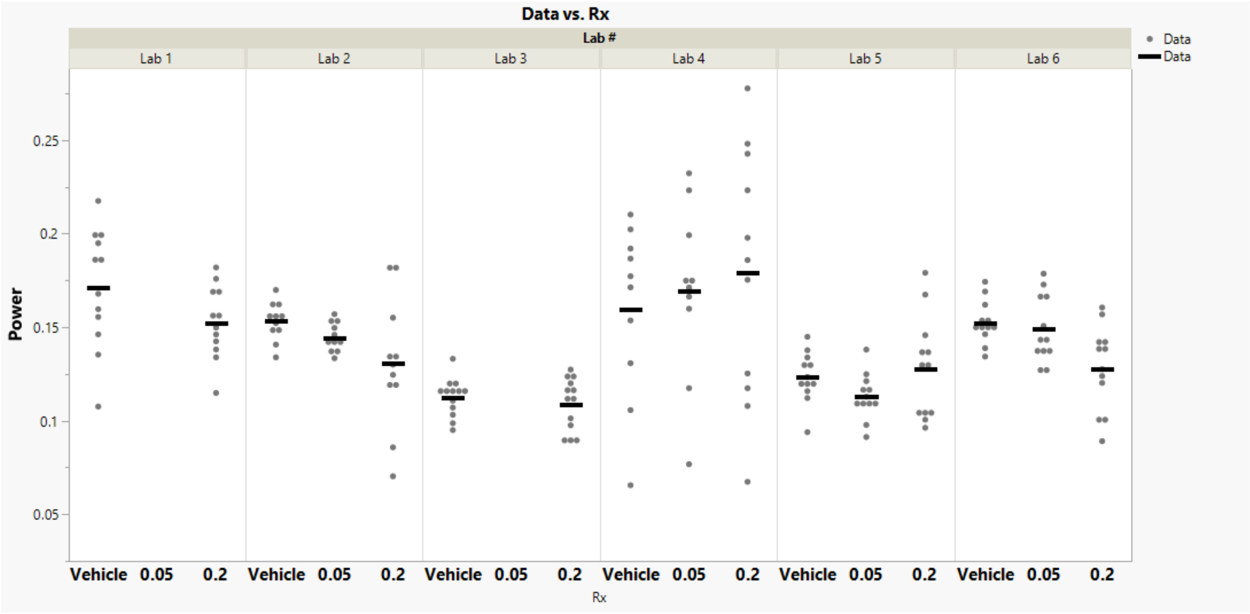

Relative Theta-2 (6-7.9 Hz) Power

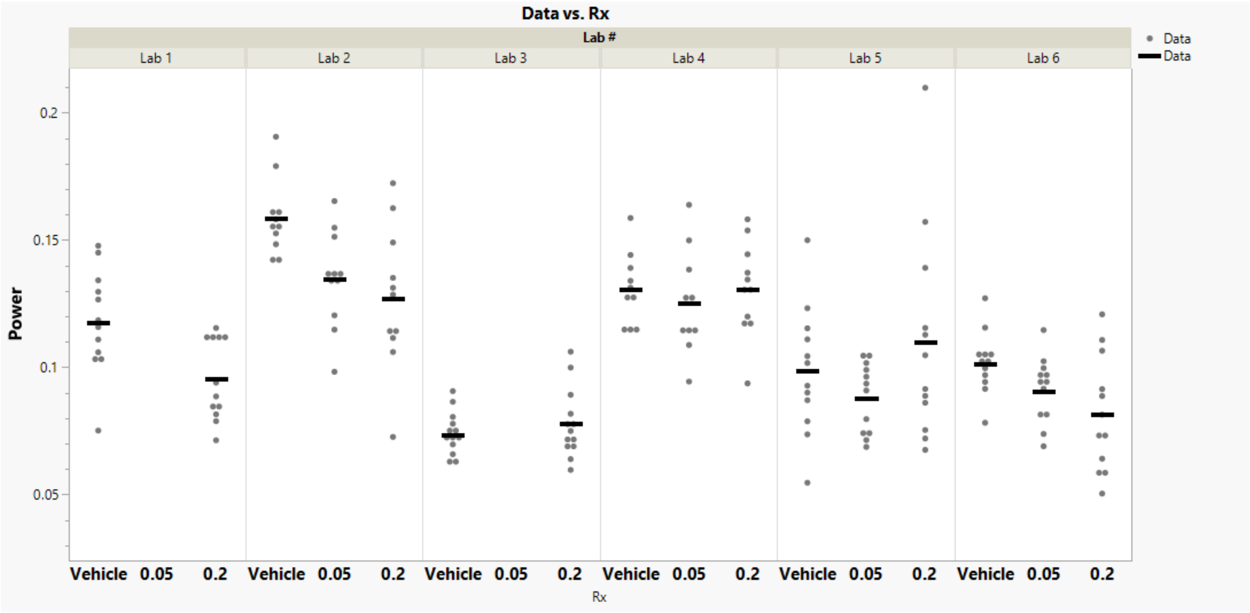

Relative Alpha-1 (8-10.9 Hz) Power

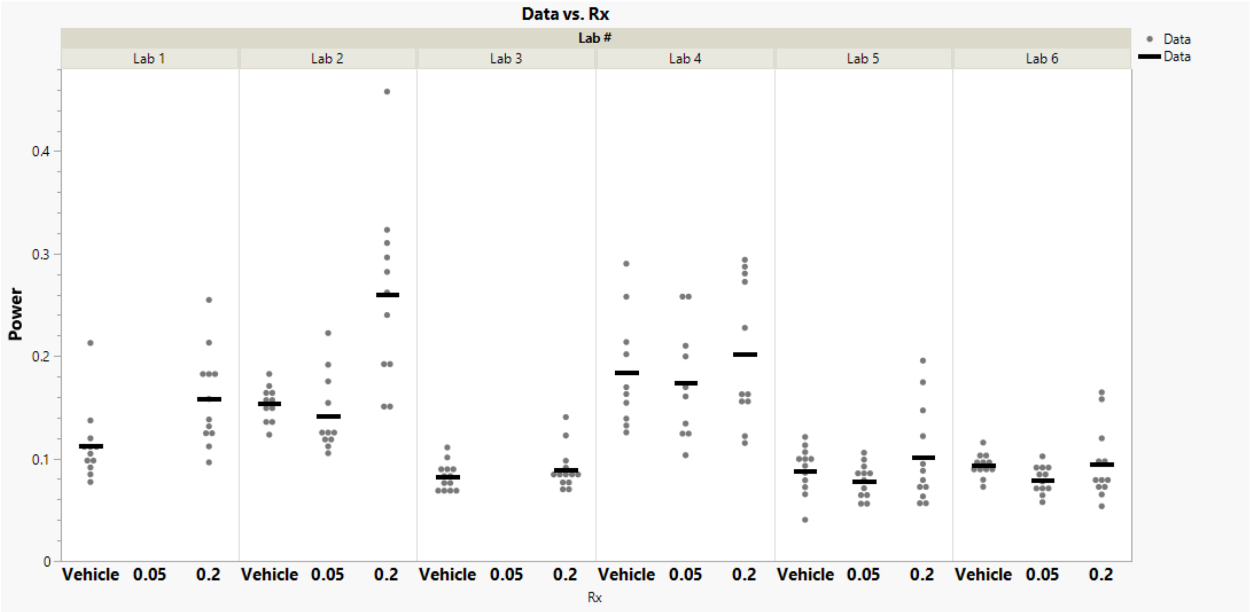

Relative Alpha-2 (11-13.9 Hz) Power

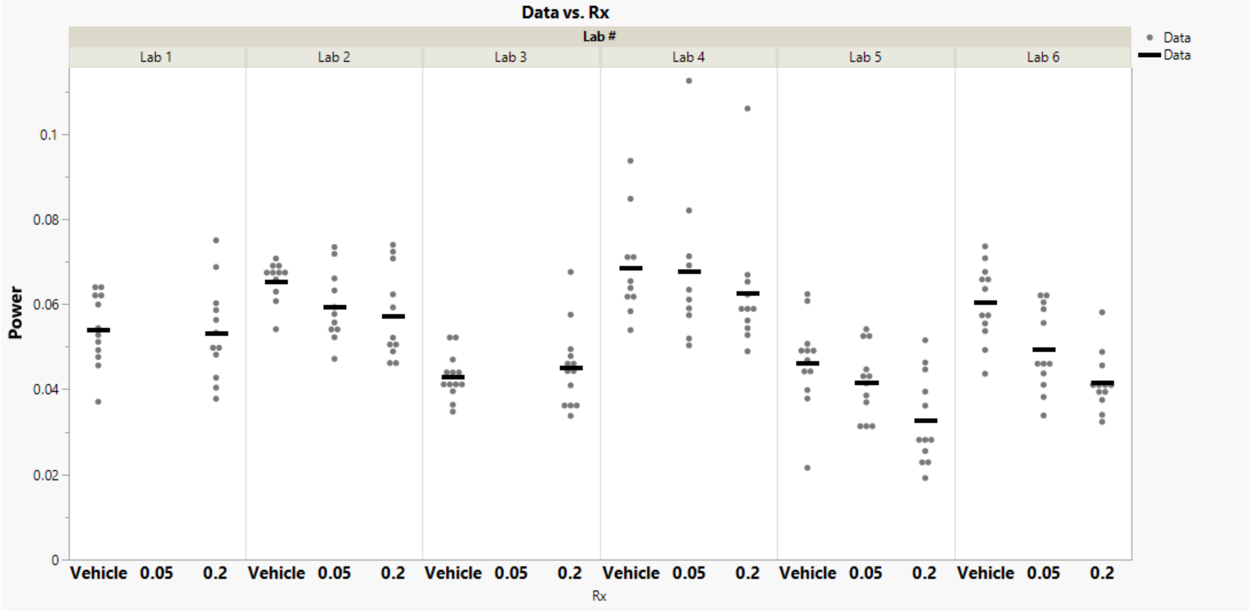

Relative Beta-1 (14-17.9 Hz) Power

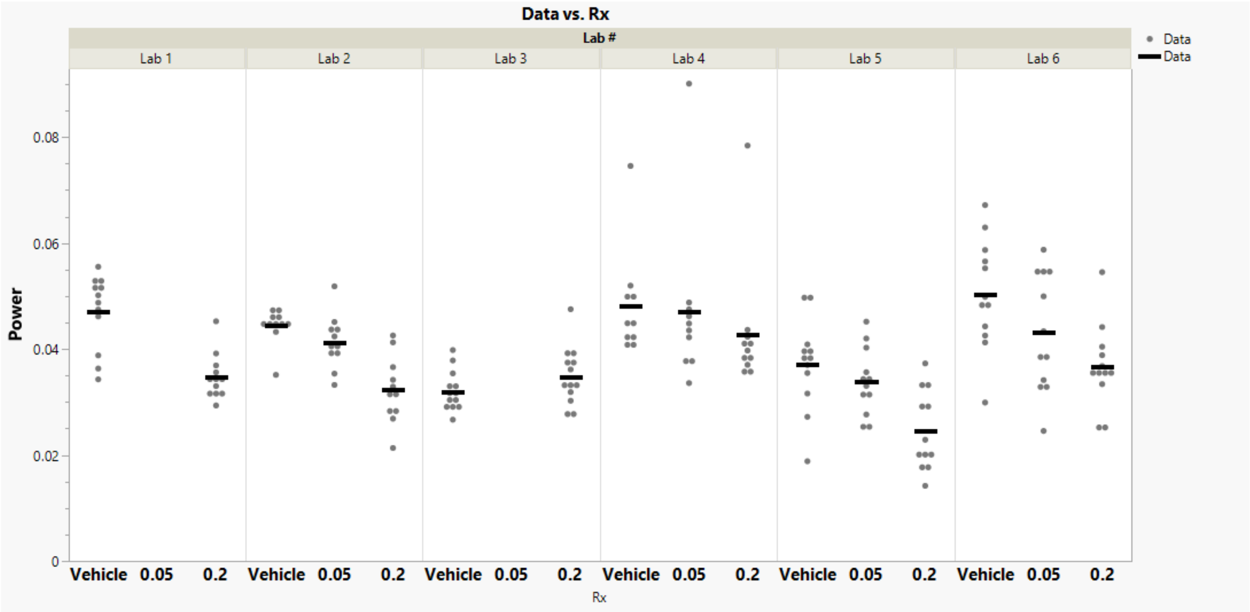

Relative Beta-2 (18-31.9 Hz) Power

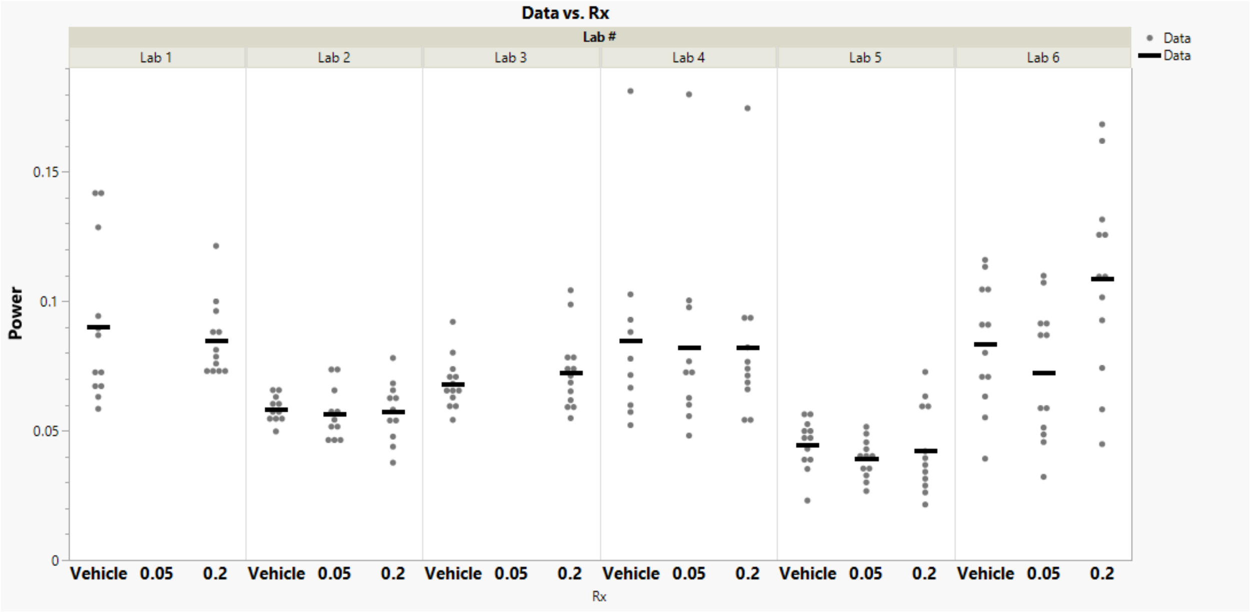

Relative Gamma (32-48 Hz) Power

Note this is the same as Figure 16.

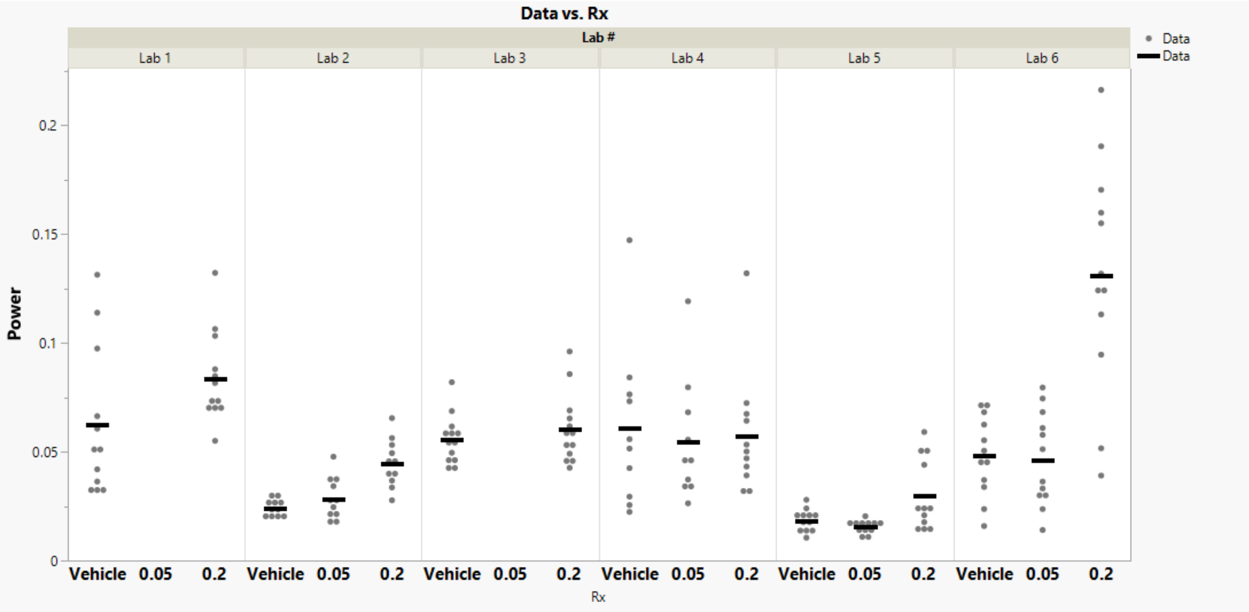

Percent Change from Baseline using Absolute Delta (1-3.9 Hz) Power

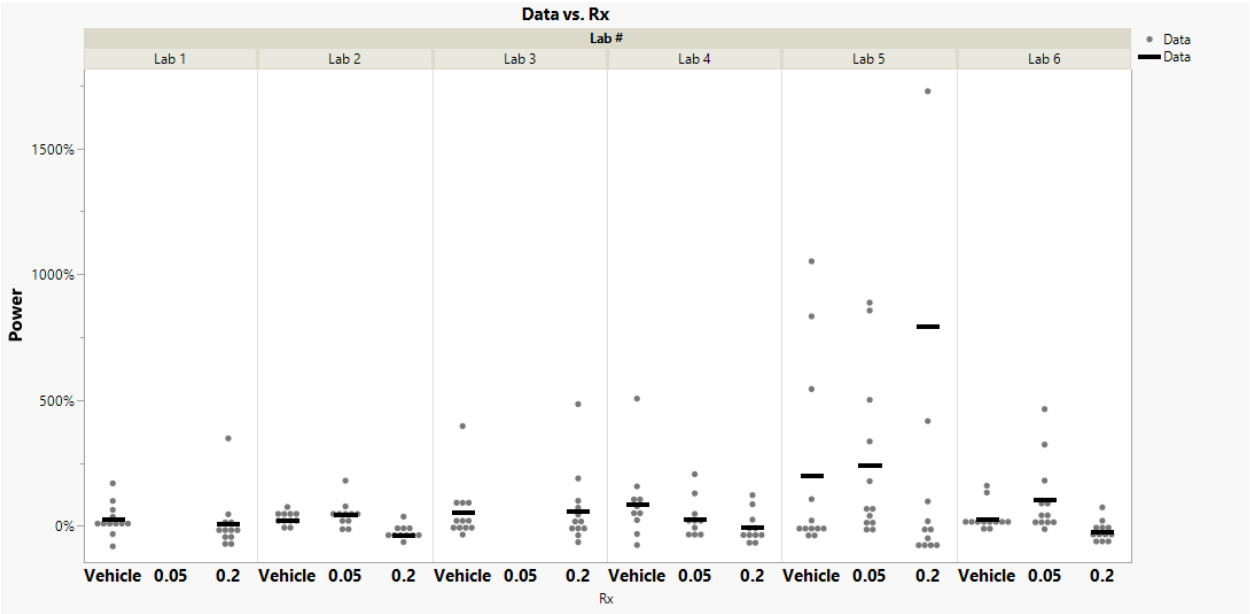

Percent Change from Baseline using Absolute Theta-1 (4-5.9 Hz) Power

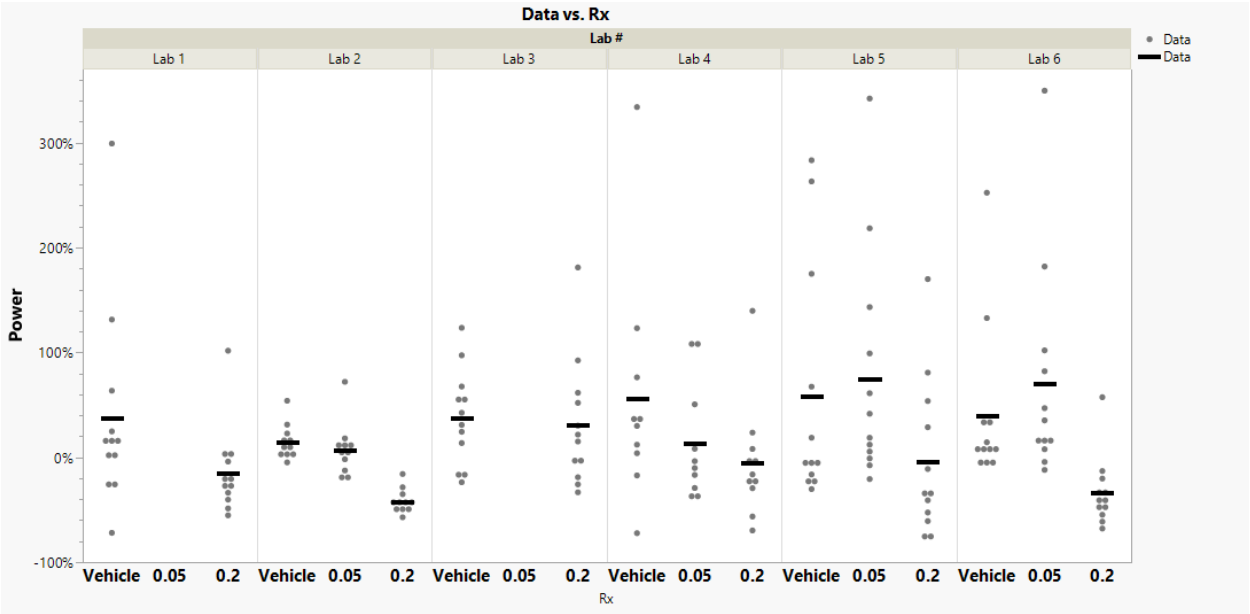

Percent Change from Baseline using Absolute Theta-2 (6-7.9 Hz) Power

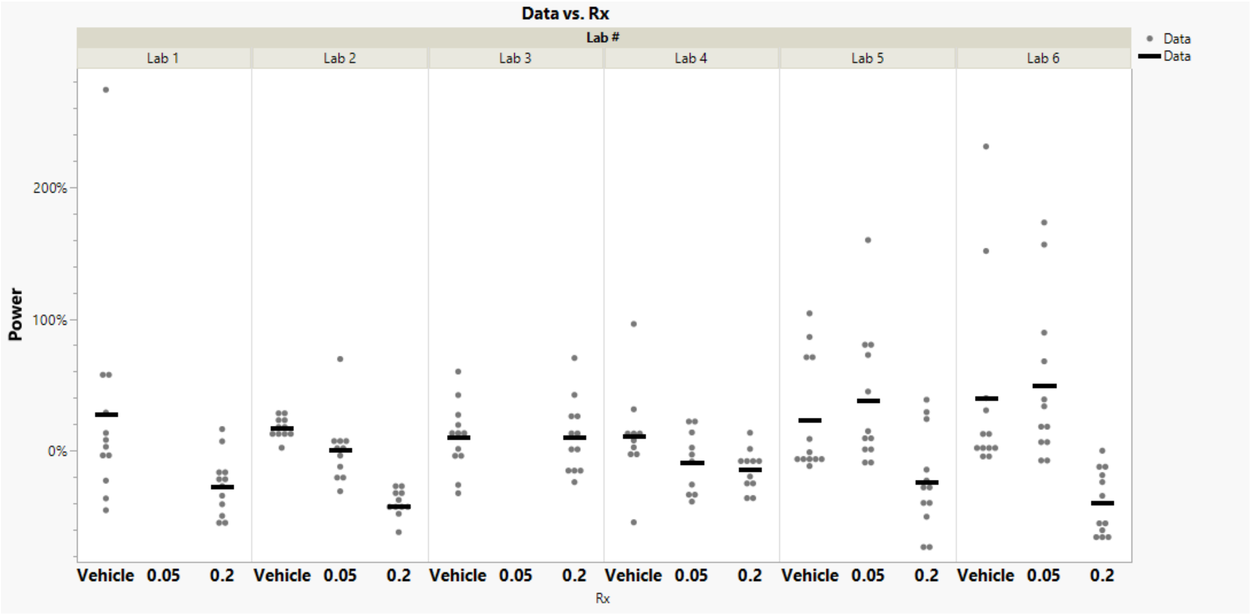

Percent Change from Baseline using Absolute Alpha-1 (8-10.9 Hz) Power

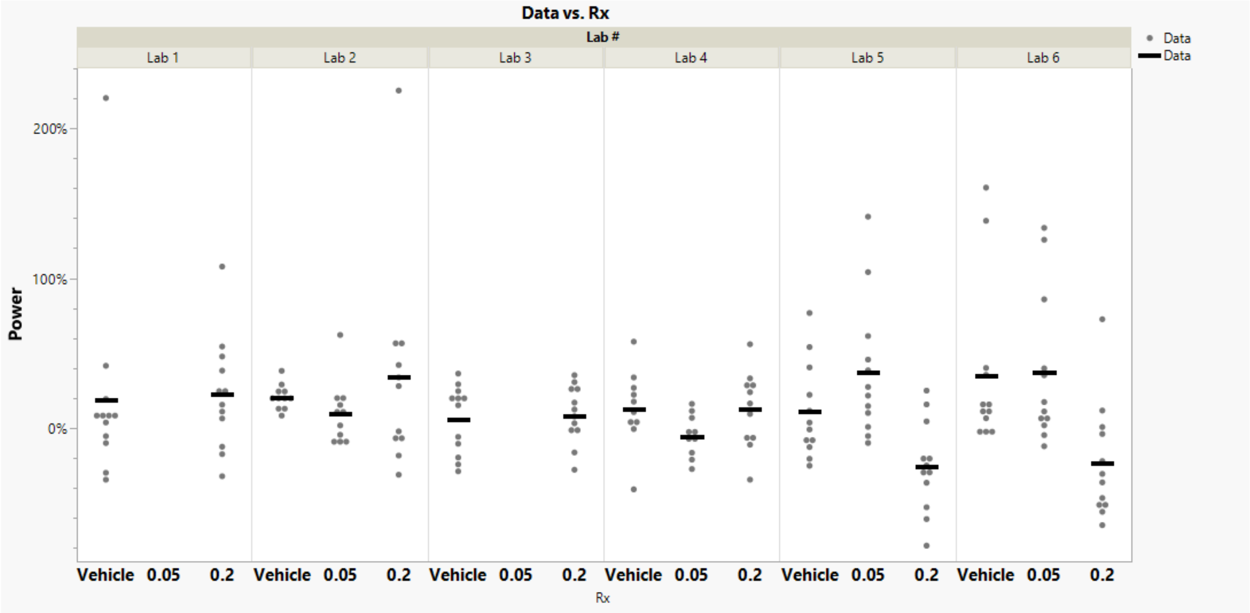

Percent Change from Baseline using Absolute Alpha-2 (11-13.9 Hz) Power

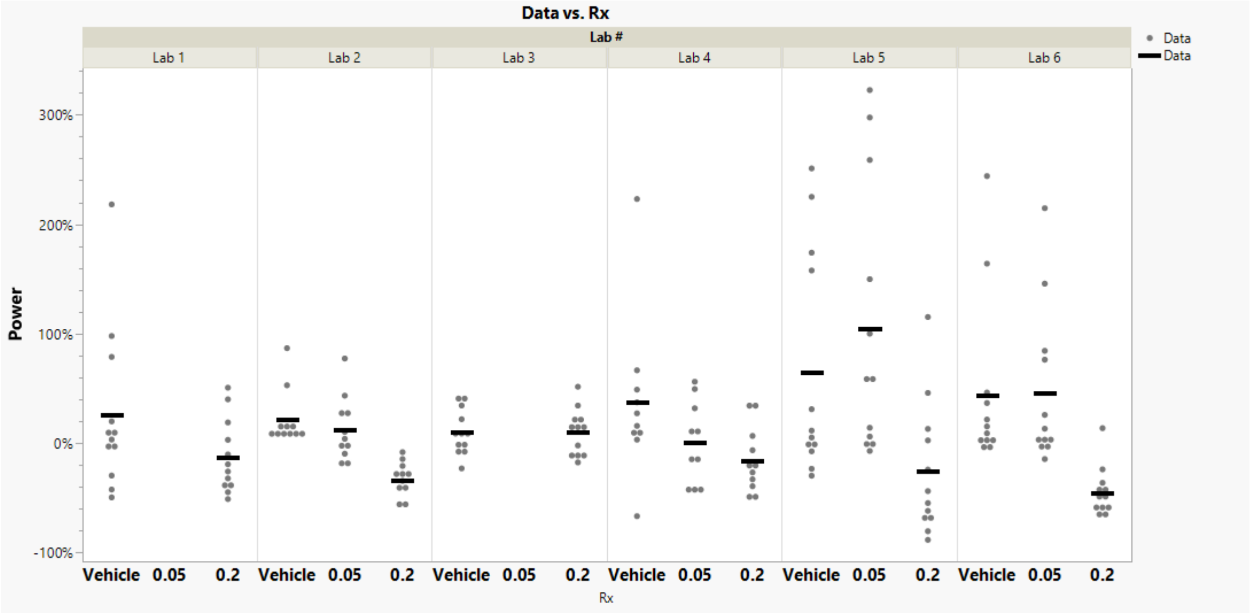

Percent Change from Baseline using Absolute Beta-1 (14-17.9 Hz) Power

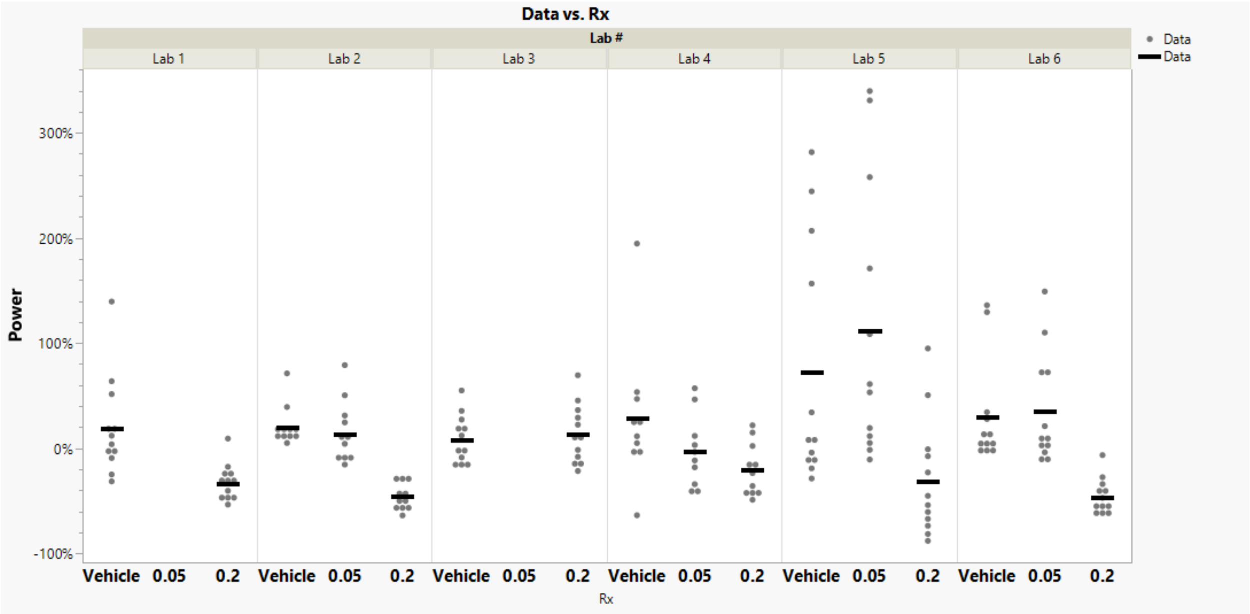

Percent Change from Baseline using Absolute Beta-2 (18-31.9 Hz) Power

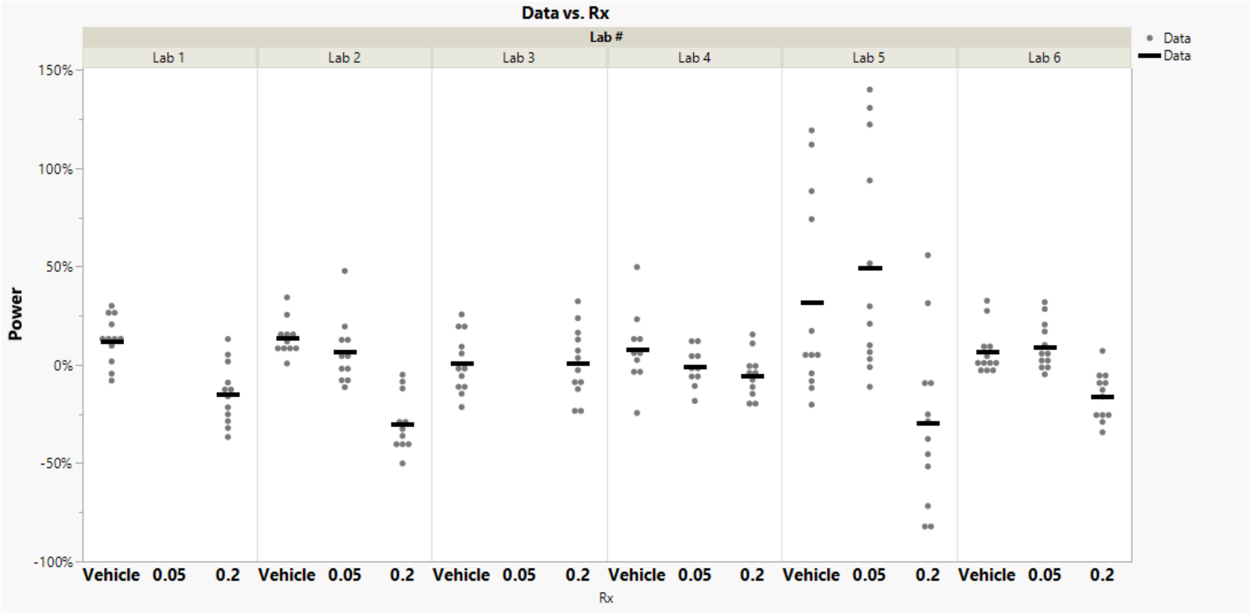

Percent Change from Baseline using Absolute Gamma (32-48 Hz) Power

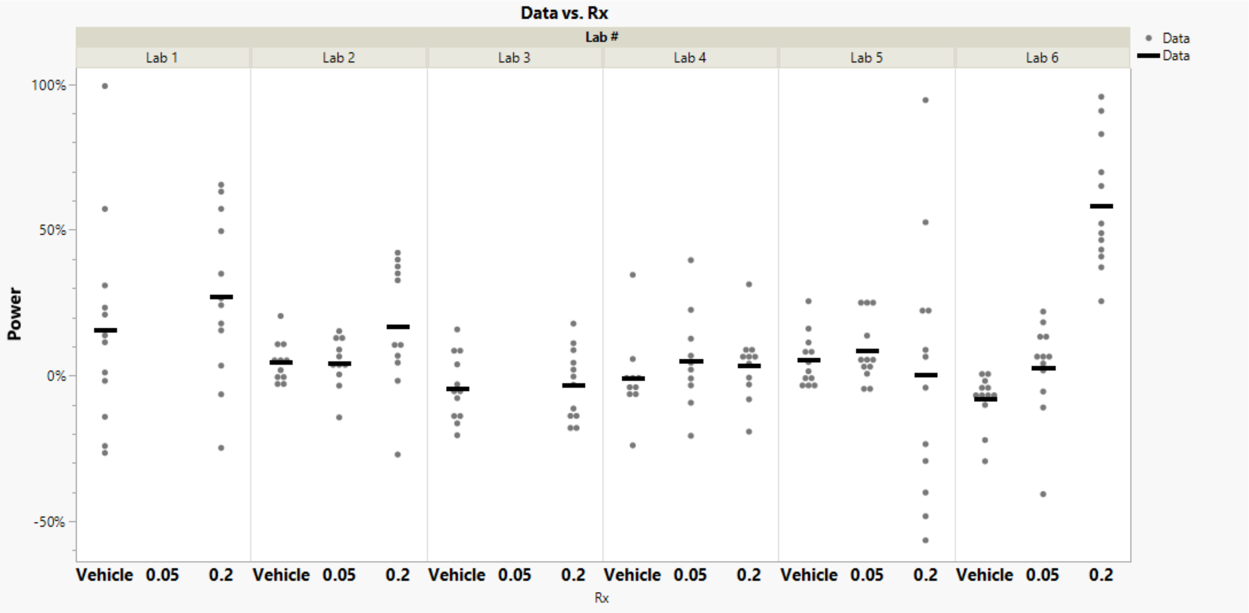

Supplement: S1 Fig — The figures show estimated means (bars) and individual data points for absolute power, relative power, or percent change from baseline calculated using absolute power across all frequency bands for each treatment group (vehicle, MK-801 at 0.05 and 0.2 mg/kg) when tested during the Ring-Testing Phase by Laboratory. All data were analysed centrally. No statistical comparisons were performed on these data. (PDF) [file pone.0309521.s012.pdf]
